# Performance evaluation of Vietnamese industrial goods and services during and post-COVID-19 era based on multi-criteria decision-making methods

**DOI:** 10.1371/journal.pone.0323764

**Published:** 2025-05-28

**Authors:** Chia-Nan Wang, Tram Thi Mai Nguyen, Nhat-Luong Nhieu, Yu-Chi Chung

**Affiliations:** 1 Department of Industrial Engineering and Management, National Kaohsiung University of Science and Technology, Kaohsiung, Taiwan; 2 Department of Business Administration, Faculty of Economics, Ho Chi Minh City University of Technology and Education, Ho Chi Minh City, Vietnam; 3 College of Technology and Design, University of Economics Ho Chi Minh City, Ho Chi Minh City, Vietnam; BRAC Business School, BRAC University, BANGLADESH

## Abstract

The industrial goods and services sector is crucial for the advancement of the Vietnamese economy in terms of its substantial economic contribution and positive impact on employment. Performance evaluation has become critical in this industry, which has constantly developed and had an intensive rivalry. This paper aims to analyze the performance of industrial goods and services firms during and after COVID-19 using an objective integrated multi-criteria decision-making technique. This study suggests a three-phase model. Criteria Importance Through Intercriteria Correlation (CRITIC) eliminates human judgment errors, increases accuracy, and maintains objectivity in the evaluation variable weighting phase. Then, Evaluation based on Distance from Average Solution (EDAS) and Technique of Order Preference Similarity to the Ideal Solution (TOPSIS) are used as effective cross-validation techniques to evaluate and rank forty-five Vietnam Stock Exchanges-listed firms for each year from 2020 to 2022. The reliability of the CRITIC-based weights is verified by the Statistical Variance Procedure. The research results reveal that the debt term structure is the most vital among the fifteen financial research indicators reflecting a business’s solvency, profitability, growth, operating efficiency, and capital structure. Additionally, the research findings indicate discrepancies in the rankings produced by EDAS and TOPSIS. However, the disparities are not grave, and the top and bottom positions, in particular, remain consistent between the two approaches. PDN was the best firm during COVID-19 and was succeeded by CIA after the pandemic. Pursuing digital transformation, sustainable development, and keeping inventory turnover at high levels are common characteristics of successful businesses in this industry. For the first time, the article provides a performance analysis of Vietnamese industrial goods and services firms. It is a significant reference for domestic and international investors in portfolio selection, financial institutions in loan approval, managers and policymakers in planning and policy development, and researchers conducting investigations within this domain.

## 1. Introduction

The COVID-19 pandemic has seriously influenced the performance of various industries globally [1]. The industrial goods and services sector is among Vietnam’s most adversely affected fields [2]. This sector is one of the essential pillars of the national economy and a significant contributor to Vietnam’s GDP structure, accounting for roughly 40% annually [3]. Moreover, it generates numerous job opportunities, mitigating unemployment and enhancing residents’ living standards. The labor force in this industry constituted 16.8% of total employment across all fields in the first half of 2022 [4]. In addition, this field also contributes considerably to Vietnam’s trade balance, representing 86% of total export turnover in 2022 [5]. Besides, Vietnam has drawn significant foreign investment capital into this area, accounting for 61% of total FDI by 2022 [6]. The pandemic spread globally, causing the supply chain to break, the domestic market demand to decrease significantly, and limited exports, making it difficult for the whole industry, especially the industrial goods and services sector. However, thanks to the Vietnamese government’s stringent control of the COVID outbreak, the EU-Vietnam Free Trade Agreements (EVFTA) were signed in 2020. Therefore, the industrial products and services sector continued to experience growth, with the index of industrial production rising by 3.36% relative to 2019 [7]. Each enterprise in this industry possesses varying capacities for recovery and growth post- pandemic. Consequently, COVID- 19 tests the companies’ ability to react to unforeseeable shocks, revealing their actual health.

The enterprise’s health is reflected in its performance assessment results. The process of collecting and analyzing data to control resource utilization and the output of production or services is called corporate performance evaluation. A performance assessment is critical in the decision-making processes of various entities, including banks and credit institutions, who use the data to forecast risks and decide whether or not to give loan reliability. Additionally, investors can choose effective investment portfolios, while analysts within organizations can accurately comprehend their position within the sector and construct appropriate strategies.

According to the literature, two commonly used methods for evaluating enterprise performance are assessing based on financial ratios derived from financial statements and non-financial indexes. There are numerous approaches for measuring based on financial information, and the most often used among non-parametric methods is the Data Envelopment Analysis (DEA), which is applied individually or in conjunction with different techniques for improvement [8]. Hassan et al. combine the DEA and Decorate Ensemble Method to classify 53 listed companies on the Amman Stock Exchange based on 11 financial variables [9]. Another research uses the Grey Relational Analysis integrated with DEA to analyze and rank 35 urban water and sewage firms in Iran [10]. In addition, the scholars use a variety of methodologies for determining and analyzing the health of organizations, such as A Knowledge-Based Decision Support System (KDSS) and Formulate Dynamic Unsupervised Machine Learning Algorithm [11].

Among these techniques, Multi-Criteria Decision-Making (MCDM) methods are known as valuable tools for evaluating and rating numerous alternatives following multiple established criteria [12]. There are several MCDM approaches providing satisfactory results in a variety of sectors [13]. The Criteria Importance Through Intercriteria Correlation (CRITIC) emerged as a widely used MCDM method [14]. This approach considers the intercorrelations between criteria to determine their relative importance [15]. CRITIC provides a more nuanced assessment of the criteria’s significance by constructing a correlation matrix and deriving criteria weights from eigenvalues. This method is beneficial when dealing with correlated criteria, avoiding redundant information. However, CRITIC assumes linear relationships and requires subjective judgment in determining the number of eigenvalues to use, which should be considered during its application. In addition, Distance-based MCDM methods are a family of decision-making techniques that help individuals or organizations evaluate and prioritize alternatives based on multiple criteria or attributes [16]. These methods are commonly employed in various fields, such as business, engineering, finance, and environmental sciences, where complex decisions must be made considering multiple conflicting factors [17,18]. The fundamental idea behind distance-based MCDM methods is to measure the similarity or dissimilarity between alternatives and a reference point (ideal or nadir) based on the criteria used for evaluation. By calculating distances, these methods aim to identify the best alternative [19] that closely aligns with the reference point and, therefore, achieves the most favorable outcomes [20]. Among them, the Evaluation based on Distance from Average Solution (EDAS) and Technique of Order Preference Similarity to the Ideal Solution (TOPSIS) developed as prevalent distance-based techniques for decision-making challenges. The EDAS is a straightforward distance-based multiple-criteria decision-making method that ranks alternatives by measuring their distances from the average performance across all criteria [21,22]. By normalizing criterion values and calculating Euclidean distances, EDAS identifies alternatives that closely match the average solution, making it suitable for situations where decision-makers seek balanced performance. Besides, TOPSIS has also been proven to be an efficient approach widely utilized throughout multiple domains. In 1981, Hwang and Yoon presented TOPSIS as a simple method for ranking that could be applied theoretically and practically [23]. Unlike EDAS, based on distance from the average solution, the critical principle of TOPSIS is that the best option must have the smallest distance from the positive ideal solution and the most considerable distance from the negative ideal solution. Despite TOPSIS’s straightforwardness, it can effectively address complex problems, such as evaluating and ranking with interval data [24]. Because of the above-mentioned notable benefits, numerous scholars have employed TOPSIS independently or in conjunction with other methods for assessing and rating. Several investigations employ various weighting techniques, such as the Analytic Hierarchy Process (AHP) [25], Fuzzy Analytic Hierarchy Process (FAHP) [26,27], CRITIC [28], entropy [29] and the Fuzzy-Based Decision-Making Trial And Evaluation Laboratory (DEMATEL) [30], etc., in combination with TOPSIS to assess efficiency. Moreover, numerous publications compare it to other approaches, such as Multiple Attribute Utility Theory (MAUT) [31], and Vlse Kriterijumska Optimizacija Kompromisno Resenje (VIKOR) [13]. Additionally, fuzzy MCDM approaches are employed in many studies to remove bias by human subjective judgments [32–35]. However, these fuzzy techniques merely reduce the subjective influence in calculating rather than entirely eliminate. Inspired by this point and the above-mentioned Distance-based MCDM approaches, a three-phase integrated methodology is proposed for the decision-making problem in this study.

The above analysis indicates that the Vietnamese industrial goods and services sector is crucial to the economy, highlighted by the following significant points: (i) Significantly contributing to GDP. (ii) Constituting a significant portion of total export revenue. (iii) Creating numerous employment opportunities to address the unemployment situation. (iv) Capturing above fifty percent of the total foreign direct investment entering Vietnam. Furthermore, this is a critical industry that the government prioritizes for developmental support. Consequently, the performance of firms in this domain impacts individual enterprises and the Vietnamese economy. So, assessing enterprises’ performances and lessons gained from typical firms serve as valuable references for policymakers in developing supportive policies for this sector, particularly in the aftermath of the epidemic.

Additionally, Vietnam is a rising star in Southeast Asia, predicted by the World Bank and HSBC as the second fastest-growing economy in the area. In 2024, the Vietnamese stock market sustained a growth rate of 12% for the VN-Index and aimed to become an emerging market in 2025 as the government’s development strategy [36]. As a result, this stock market captivates investors’ interest. Investors mostly base their decisions on economic metrics, company-specific information, and historical stock data. Precisely assessing enterprises within the same industry can reflect their position compared to rival companies, showing their advantages, drawbacks, opportunities, and challenges [37]. Therefore, the evaluation results have significant value for investors in their analysis and distinguishing between successful and unsuccessful businesses, aiding their decisions in investment portfolio selection.

Furthermore, to thrive in the challenging competitive landscape, Vietnamese industrial goods and services enterprises must continually scrutinize their competitors’ operations and financial performance to strengthen their market position. Businesses need to review their financial information and analyze the fiscal condition of the leading company in the same industry to generate successful strategies for future growth. Thus, assessing the performance of enterprises within the same sector is crucial for both the firms and the industry. Besides, companies in this sector have capital demands for their operations and investments, with loans being one of the prominent sources of capital. Financial performance indicates an organization’s stability and fiscal health and is an appropriate metric for assessing its prestige and capacity for debt repayment. So, evaluating the financial performance of companies in this sector is also crucial for financial institutions when determining loan approvals and credit policies. Further, this performance evaluation is especially significant when carried out during and after COVID-19, as it accurately reflects the actual ability of firms.

Although it is vital, as stated in the above analysis, the topic of this study has never been investigated previously in other research works. This situation motivates the authors to undertake this research. The study attempts to fill the gap in the literature by presenting for the first time a three-phase integrated MCDM approach to analyze the performance of firms in the Vietnamese industrial goods and services sector during and after the pandemic, which is a previously unexplored research area. In this article, three multi-criteria decision-making methods are deliberately chosen to enhance both objectivity and comprehensiveness. First, the CRITIC technique is employed to determine indicator weights using only data-driven variability and intercorrelations. This eliminates the need for subjective expert input, thereby reducing bias. Second, EDAS is used to rank alternatives based on their distance to the average solution, offering insights into how each firm’s performance compares to the overall midpoint. Third, TOPSIS is applied to rank alternatives by measuring proximity to positive and negative ideal solutions, thus providing a complementary viewpoint on optimal performance.

The highlighted contributions of the research are described as follows. First, this research contributes to the industrial goods and services sector and literature by analyzing firms with multi-criteria decision-making methods systematically integrated across three stages. It provides domestic and international stakeholders with the opportunity to determine the performance of industrial goods and services companies to make more effective strategic decisions. This study also presents statistical information on industrial goods and services firms to enhance industry knowledge. Second, the results obtained from EDAS and TOPSIS are analyzed in parallel to observe any changes or inconsistencies. While slight differences in firm rankings arise due to the methods’ distinct distance-based principles, significant disparities do not emerge. In particular, top and bottom-ranked companies remain relatively consistent across both approaches. This dual-ranking process reveals subtleties in each firm’s performance, validates the robustness of the outcomes, and minimizes dependence on a single ranking concept. By synthesizing objective weighting from CRITIC with dual distance-based methods for evaluation, a more reliable and well-rounded assessment is achieved. Consequently, the suggested method can be extended to evaluate corporate performance in other domains as an effective metric. Finally, an analysis of business performance over the years, particularly during and after COVID-19, deeply reflects the proper health of enterprises. Subsequently, identifying and analyzing several typical enterprises to derive features and lessons from these organizations. These are valuable references for corporations and policymakers in strategizing and formulating future policies.

The rest of the paper is organized as follows. In the next section, a three-phase integrated methodology is presented. Section 3 describes the financial variables and the data collection process. Subsequently, the computation results and relevant discussions are introduced in section 4. The final section summarizes the study’s key findings, contributions, and future research directions.

## 2. Methodology

**Fig 1:**
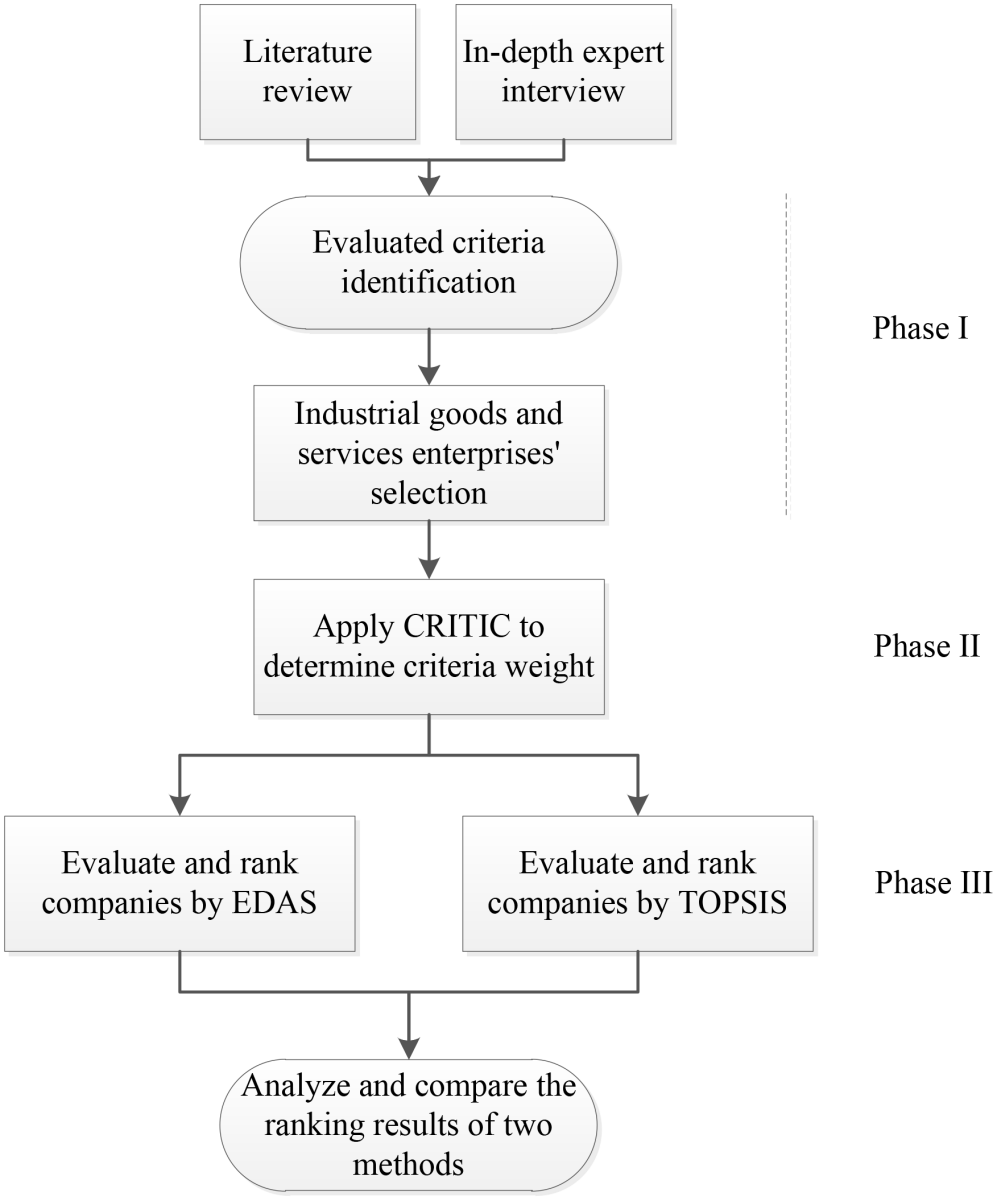
Proposed methodology.

As shown in Fig 1, this study’s proposed methodology comprises three comprehensive phases to facilitate effective decision-making processes. In Phase I, the methodology begins with indicator identification, where a two-fold approach is adopted. Firstly, an extensive literature review is conducted to identify relevant indicators and gain insights into their significance. Secondly, expert deep interviews are conducted to gather domain-specific knowledge and ensure a comprehensive set of indicators. Subsequently, the industrial goods and services company selection phase narrows down the potential companies that will be evaluated based on the identified indicators. The data collection follows, where relevant data related to the selected companies and indicators are gathered from various reliable sources. The decision matrix construction step involves integrating the collected data into a structured matrix, facilitating comparative analysis of the companies across the identified indicators. In Phase II, the methodology delves into a more profound analysis. The first step, indicator correlation computation, aims to assess the interrelationships among the indicators, offering valuable insights into their interconnections. Information content estimation follows, wherein the importance and relevance of each indicator are determined, aiding in establishing the significance of individual indicators in the decision-making process. The weighting step allocates appropriate weights to each indicator based on their importance, ensuring a fair representation of their contributions. Consequently, in Phase III, the methodology moves towards reaching a conclusive decision. At this stage, EDAS and TOPSIS are utilized separately to evaluate enterprises’ performance from different perspectives. According to EDAS, the average solution is identified for each indicator. The methodology computes the weighted positive and negative distance from the average solution to refine the evaluation further. Each company’s appraisal score is calculated, reflecting its overall performance and competitiveness concerning the identified indicators. Ultimately, the TOPSIS approach assesses performance and classifies the evaluated organizations. The decision matrix is first normalized to remove the varying dimensions of the indicators, hence facilitating the evaluation process. A weighted decision matrix is established to identify the positive ideal solution_ PIS and negative ideal solution_ NIS, followed by the sequential calculation of the distance from each alternative to both PIS and NIS. Eventually, the score and rating of each alternative are determined by the principle that the best alternative is not only nearest to the PIS but also farthest from the NIS. The procedure of the proposed methodology is visualized in Fig 1.

Although CRITIC, EDAS, and TOPSIS have each been employed in prior studies, their integration in this research differs from existing approaches. In most traditional applications, expert judgments or subjective inputs are relied upon to assign weights to each indicator. In contrast, the CRITIC technique is utilized in this study to determine these weights strictly based on data variability and intercorrelations, thereby reducing subjectivity. Furthermore, two distance-based MCDM methods—EDAS and TOPSIS—are employed consecutively to cross-validate and enrich the decision-making results. This dual-perspective analysis ensures that conclusions are not biased by a single evaluation principle, thus yielding a more robust and comprehensive assessment of company performance. By combining objective weighting and distance-based techniques, the proposed methodology minimizes human bias and enhances the reliability of the final rankings, thereby distinguishing it from other commonly used MCDM frameworks.

**Phase I**

Step 1: Based on references and in-depth interviews with experts, a group of evaluation indicators is determined.

Step 2: A shortlist of industrial goods and services companies (Iis established based on their conditions.

Step 3: The data of the selected companies corresponding to the evaluation indicators are collected.

Step 4: After data processing, a decision matrix (X) is constructed, as shown in Equation (1).


$$X=\;{\left[x_{ij}\right]}_{IXJ}$$ (1)

where $x_{ij}$ expressed the performance of ith company corresponding to jth indicator.

**Phase II: CRITIC**

Step 1: Based on the concept of the ideal point, the decision matrix is normalized as Equation (2)- (3).


$$Y=\;{\left[y_{ij}\right]}_{IXJ}$$ (2)

$$y_{ij}=\frac{x_{ij}-x_{j}^{*}}{x_{j}^{**}-x_{j}^{*}},\;j=1\ldots J$$ (3)

where $x_{j}^{**}$ and $x_{j}^{*}$ expressed the best and worst performance of companies corresponding to jth indicator, respectively.

Step 2: To examine the jth indicator in isolation, the performance vector $\left(z_{j}\right)$ of all companies is expressed as Equation (4). Then, the standard deviation $\left(\sigma_{j}\right)$ of $z_{j}$ is computed as Equation (5).


$$Z_{j}=\;\left[y_{1j},y_{2j},\ldots ,y_{ij},\ldots ,y_{Ij}\right]$$ (4)

$$\sigma_{j}=\;\sqrt{\frac{\sum_{i=1}^{I}{\left|y_{ij}-{\overset{―}{y}}_{j}\right|}^{2}}{I}}\;where\;{\overset{―}{y}}_{j}=\frac{\sum_{i=1}^{I}y_{ij}}{I}$$ (5)

Step 3: The symmetric linear correlation coefficient matrix is constructed as Equation (6)


$$T={\left[t_{jk}\right]}_{JxJ}$$ (6)

where $t_{jk}$ represents the linear correlation coefficient between the vectors $Z_{j}$ and $Z_{k}$.

Step 4: The amount of information content of jth indicator is estimated as Equation (7).


$$C_{j}=\sigma_{j}\sum_{k=1}^{J}\left(1-t_{jk}\right),\;j=1\ldots J$$ (7)

Step 5: The higher information content implies the higher weight of the indicator.


$$w_{j}=\;\frac{C_{j}}{\sum_{j=1}^{J}C_{j}}$$ (8)

**Phase III (a): EDAS**

Step 1: Based on the normalized decision matrix, determine the average solution according to all criteria.


$$AV={\left[{av}_{j}\right]}_{1xJ}\;where\;{av}_{j}=\frac{\sum_{i=1}^{I}y_{ij}}{I}$$ (9)

Step 2: The weighted positive and negative distance from average solution matrices are constructed as Equations (10)-(11).


$$WPD={\left[{wpd}_{ij}\right]}_{IxJ}$$ (10)

$$WND={\left[{wnd}_{ij}\right]}_{IxJ}$$ (11)

If jth indicator is the benefit indicator.


$${wpd}_{ij}=w_{j}\left(\frac{\max\left(0,\left(y_{ij}-{av}_{j}\right)\right)}{{av}_{j}}\right)$$ (12)

$${wnd}_{ij}=w_{j}\left(\frac{\max\left(0,\left({av}_{j}-y_{ij}\right)\right)}{{av}_{j}}\right)$$ (13)

If jth indicator is the non-benefit indicator.


$${wpd}_{ij}=w_{j}\left(\frac{\max\left(0,\left({av}_{j}-y_{ij}\right)\right)}{{av}_{j}}\right)$$ (14)

$${wnd}_{ij}=w_{j}\left(\frac{\max\left(0,\left(y_{ij}-{av}_{j}\right)\right)}{{av}_{j}}\right)$$ (15)

Step 3: The normalized sum of WPD and WND are computed as Equations (16)-(17).


$${NSP}_{i}=\frac{\sum_{j=1}^{J}{wpd}_{ij}}{\max_{i}\left(\sum_{j=1}^{J}{wpd}_{ij}\right)}$$ (16)

$${NSN}_{i}=1-\frac{\sum_{j=1}^{J}{wnd}_{ij}}{\max_{i}\left(\sum_{j=1}^{J}{wnd}_{ij}\right)}$$ (17)

Step 4: The appraisal score of companies is estimated as Equation (18). The higher the value of the appraisal score, the higher the rank.


$${AS}_{i}=\frac{{NSP}_{i}+{NSN}_{i}}{2}$$ (18)

**Phase III (b): TOPSIS**

Step 1: Based on the constructed decision matrix, establish a normalized matrix following Equation (19)


$$y_{ij}=\frac{x_{ij}}{\sqrt[{2}]{\sum_{i=1}^{m}{x_{ij}}^{2}}}$$ (19)

Step 2: A weighted decision matrix is created using Equation (20)


$$\upsilon_{ij}=\omega_{j}*\;y_{ij}$$ (20)

Where $\omega_{j}$: is the weight of the associated criteria estimated by CRITIC.

Step 3: The positive ideal solution_ PIS and the negative ideal solution_ NIS are calculated through Equations (21)- (22)


$$PIS=\left\{\upsilon_{j}^{*}\right\};\;j=1,\ldots ,n$$ (21)

$$NIS=\left\{\upsilon_{j}^{-}\right\};\;j=1,\ldots ,n$$ (22)

While,


$$\upsilon_{j}^{*}=\left\{\max\upsilon_{ij}\;if\;j\epsilon J,\min\nu_{ij}\;if\;j\epsilon J^{-}\right\}$$

$$\upsilon_{j}^{-}=\left\{\min\upsilon_{ij}\;if\;j\epsilon J,\max\nu_{ij}\;if\;j\epsilon J^{-}\right\}$$

J: is the desirable attribute

J^-^: is the undesirable attribute.

Step 4: Determine the distance between each alternative and PIS (S_i*_) via Equation (23) and NIS (Si-) via Equation (24).


$$S_{i}^{*}=\sqrt[{2}]{\sum_{j=1}^{n}({\upsilon_{ij}-\upsilon_{j}^{*})}^{2}}$$ (23)

$$S_{i}^{-}=\sqrt[{2}]{\sum_{j=1}^{n}({\upsilon_{ij}-\upsilon_{j}^{-})}^{2}}$$ (24)

Step 5: The proximity of each alternative to the ideal solution Ci* is computed in Equation (25). The proximity of Ci* to 1 indicates a higher ranking of the alternative.


$$C_{i}^{*}=\;\frac{S_{i}^{-}}{S_{i}^{-}+S_{i}^{*}};\;0{\leq \;C}_{i}^{*}\leq \;1$$ (25)

## 3. Evaluation criteria and collected data

The study samples are industrial goods and services businesses listed on the two stock exchanges in Vietnam, including HOSE and HNX, whose annual reports are published in compliance with the regulations of the State Securities Commission of Vietnam. In this study, selected firms must have accomplished information regarding their audited annual reports from 2020 to 2022, corresponding to the period during and after COVID-19. As of December 31, 2022, 45 companies that matched the requirements for accurate and completed data have been chosen as alternatives.

The financial ratios effectively reflect an organization’s performance in the market and its long-term growth potential [38]. So, financial indexes are not only valuable for assessing the overall achievement of a business [39] but also are helpful in predicting its future development [40]. Experts can analyze a comprehensive collection of financial variables from the literature [41] to choose the essential indicators. The experts consulting in this study are five financial managers with extensive expertise in credit institutions. They select the most significant criteria for measuring company performance based on their knowledge and experiences. Consequently, fifteen criteria from five groups are collected and given in Table 1, which presents companies’ profitability, growth rate, liquidity, efficiency, and leverage. The financial data is derived from the annual public financial reports of enterprises. In the study, this data was collected and supplied by Fiingroup Joint Stock Company, a prominent entity in Vietnam that provides financial information, business information, marketing research, consulting, and credit rating services.

**Table 1:** Proposed evaluation indicators.

Factor	Notation	Indicators	Calculating formula	Reference
Profitability ratios	I1	ROE	Net income/ Average total equity	[12,41,42]
	I2	ROA	Net income/ Average total assets	[12,41–43]
	I3	EPS	Net Income/ Number of Shares Outstanding	[42]
Growth ratios	I4	Sale growth rate	(Current period sales – Prior period sales)/ Prior period sales	[12,42]
	I5	Net profit margin (NPM) growth rate	(Current period NPM- Prior period NPM)/ Prior NPM *With NPM = Net Income/ Revenue*	[42]
Liquidity ratios	I6	Current ratio	Current assets/ Current liabilities	[12,41,44]
	I7	Quick ratio	(Current assets – Inventories)/ Current liabilities	[12,41,43,44]
	I8	Interest coverage	EBIT/ Interest payments	[13,43]
Efficiency ratios	I9	Inventory turnover	Cost of goods sold/ Average inventory	[12,43,45]
	I10	Payables turnover	Total purchases/ Average accounts payables	[43]
	I11	Receivable turnover	Net credit sales/ Average accounts receivable	[12,46]
	I12	Cost of goods sold/ Net revenue	Cost of goods sold/ Net revenue	[47]
	I13	Selling, General &Administrative Expenses/ Net revenue	Selling, General & Administrative Expenses/ Net revenue	[47]
Leverage ratios	I14	Debt-to-equity ratio	Total debt/ total shareholders’ equity	[42,43]
	I15	Short-term debt to total debt	Short-term debt/ total debt	[48]

Although factual metrics were extracted directly from audited financial statements during data collection, optimization concepts were inherently embedded into the methodology through the CRITIC, EDAS, and TOPSIS frameworks. Within the CRITIC method, the weighting procedure was designed to maximize the total information content of each indicator while minimizing redundancy arising from correlations. Consequently, the importance of each criterion was optimized in the final evaluation. In EDAS and TOPSIS, reference points—namely, the average solution (EDAS) and the positive/negative ideal solutions (TOPSIS)—were defined, and each firm’s performance was measured according to its distance from these benchmarks. Through this process, distance-based optimization principles were leveraged to determine how effectively alternatives aligned with optimal performance outcomes. Even though no dedicated mathematical optimization algorithm was applied for data input, the overall multi-criteria decision-making framework ensured that data and metrics were arranged and evaluated to provide robust, objective insights into the firm’s performance.

## 4. Numerical results and discussion

### 4.1. Indicator weighting

According to the proposed method, the CRITIC approach estimates the weights of the indicators deployed to evaluate firm performance. Firstly, using Equation 1, construct a decision matrix (15x45) for 2020, 2021, and 2022. Then, Equations 2 and 3 are used to normalize the matrix, as depicted in Table A1. After normalization, the symmetric matrix reflecting the linear correlation coefficient between the indices is organized following Equation 6, shown in Table A2. Each indicator’s information content can be calculated using Equations 4–7, and the corresponding weight is obtained by applying Equation 8. The same procedure is followed in 2021 and 2022 to determine the relevant weights, as summarized in Table 2.

**Table 2:** The indicators’ weight over the years.

Indicators	CRITIC method			Statistical Variance Procedure		
2020	2021	2022	2020	2021	2022
I1	0.048	0.048	0.041	0.046	0.047	0.037
I2	0.047	0.042	0.043	0.055	0.046	0.046
I3	0.060	0.065	0.046	0.064	0.092	0.043
I4	0.054	0.076	0.069	0.042	0.073	0.071
I5	0.055	0.052	0.054	0.048	0.038	0.050
I6	0.062	0.077	0.056	0.052	0.081	0.048
I7	0.079	0.059	0.055	0.068	0.054	0.046
I8	0.061	0.081	0.077	0.053	0.076	0.082
I9	0.062	0.067	0.064	0.058	0.060	0.056
I10	0.061	0.094	0.086	0.037	0.088	0.065
I11	0.090	0.078	0.067	0.088	0.063	0.049
I12	0.061	0.040	0.097	0.067	0.032	0.122
I13	0.080	0.064	0.083	0.073	0.046	0.076
I14	0.060	0.062	0.058	0.056	0.055	0.056
I15	0.119	0.095	0.104	0.191	0.149	0.153

Additionally, the Statistical Variance Procedure (SVP) is employed to verify the consistency of the CRITIC-based weights. SVP is another objective weighting technique based on the statistical variance of information, frequently utilized due to its simplicity and efficacy [49]. The comparison results in Table 2 indicate that the weights of the indicators calculated by the two approaches exhibit similarity over the years, with the mean absolute error between them of 0.01199, 0.01186, and 0.01128 for 2020, 2021, and 2022, respectively. In particular, both methods consistently point out that short-term debt to total debt (I15) is the most significant metric for assessing company performance over the years. It implies that the term structure of debt plays the most important role in reflecting the financial performance of enterprises in the Vietnamese industrial goods and services sector.

### 4.2. Companies ranking

#### 4.2.1 Evaluation based on the EDAS method.

**Fig 2:**
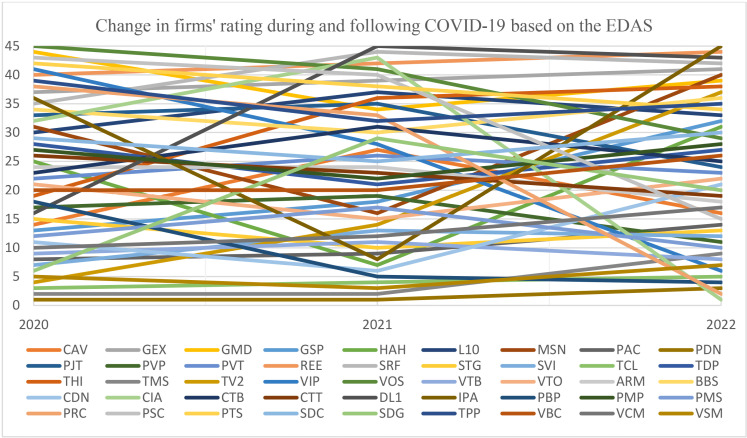
Changes in firms’ ratings while and following COVID-19 based on EDAS.

Based on the normalized decision matrix, the average solutions for all indicators are derived using Equation 9. The weighted positive and negative distance from average solution matrices are then determined by Equations 10–15, as shown in Tables A3 and A4. The normalized sum of weighted positive and negative distance is then calculated following Equations 16 and 17. Subsequently, each firm’s evaluation score and rank are determined via Equation 18. Following the same procedure as in 2020, the ranking results of 45 enterprises in the Vietnamese industrial goods and services sector during and after COVID-19 are determined, as visualized in Fig 2.

The data indicated that during the pandemic, from 2020 to 2021, PDN and TMS were the best and second companies, while VOS and DL1 were rated as the worst firms, corresponding to each year. In 2022, following COVID-19, CIA and PRC rapidly ascended from the 43rd and 33rd to the top two ranks, with CIA obtaining the highest, while IPA suddenly fell to the lowest. The fluctuations in evaluated firms’ rank under analysis years differ, with TCL, PDN, and VTB experiencing little change. In contrast, CIA, PRC, and IPA emerged as the most considerable volatility. Furthermore, comparing ranking fluctuations between 2020 and 2021 with those from 2021 to 2022 reveals that the latter has a more significant change, with an average standard deviation of 6.73 compared to 5.06 for the former. These results demonstrate that ranking fluctuations are more prominent following COVID-19 than throughout the epidemic. This shows that more extended periods more effectively reflect organizations’ genuine capabilities in response to unexpected shocks than shorter periods.

#### 4.2.2. Evaluation based on the TOPSIS method.

**Fig 3:**
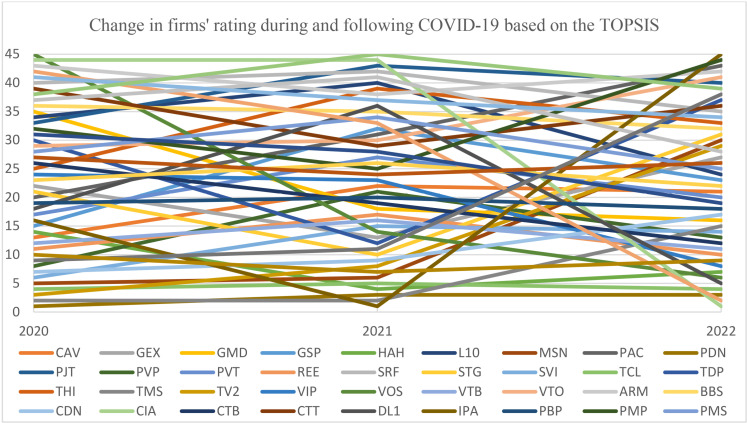
Changes in firms’ ratings while and following COVID-19 based on TOPSIS.

The first step in the ranking procedure, following TOPSIS, is generating a normalized matrix using Equation 19, as illustrated in Table A5. A weighted normalized matrix is subsequently constructed by applying the weights determined in phase II to each element through Equation 20. Equations 21–24 are employed in the next stage to assess the distance from the alternatives to the positive and negative ideal points, consequently calculating the performance score and ranking as per Equation 25. Table 3 summarizes the distances, scores, and rankings among companies in 2020. Additionally, implementing a consistent assessment procedure for all analysis years and the ranking results are described in Fig 3.

**Table 3:** Companies Ranking in 2020 based on TOPSIS.

Company	S_i*_	S_i-_	Score	Rank
CAV	0.0865	0.0740	0.4610	13
GEX	0.0899	0.0687	0.4331	22
GMD	0.0933	0.0656	0.4130	35
GSP	0.0817	0.0683	0.4554	15
HAH	0.0839	0.0707	0.4572	14
L10	0.0937	0.0659	0.4130	34
MSN	0.0824	0.0798	0.4919	5
PAC	0.0863	0.0680	0.4406	20
PDN	0.0676	0.0938	0.5812	1
PJT	0.0878	0.0627	0.4166	33
PVP	0.0802	0.0741	0.4803	8
PVT	0.0860	0.0690	0.4452	17
REE	0.0852	0.0734	0.4627	11
SRF	0.0977	0.0641	0.3961	40
STG	0.0868	0.0683	0.4402	21
SVI	0.0849	0.0821	0.4917	6
TCL	0.0762	0.0764	0.5007	4
TDP	0.0919	0.0664	0.4195	30
THI	0.0903	0.0678	0.4289	25
TMS	0.0676	0.0831	0.5513	2
TV2	0.0775	0.0846	0.5218	3
VIP	0.0908	0.0688	0.4308	24
VOS	0.1128	0.0476	0.2968	45
VTB	0.0820	0.0702	0.4615	12
VTO	0.0876	0.0637	0.4209	29
ARM	0.1021	0.0583	0.3635	43
BBS	0.0954	0.0668	0.4117	36
CDN	0.0804	0.0751	0.4829	7
CIA	0.1210	0.0531	0.3051	44
CTB	0.0932	0.0699	0.4286	26
CTT	0.1006	0.0676	0.4020	39
DL1	0.0906	0.0727	0.4452	18
IPA	0.0879	0.0725	0.4520	16
PBP	0.0862	0.0687	0.4435	19
PMP	0.0932	0.0670	0.4183	32
PMS	0.0904	0.0670	0.4257	28
PRC	0.0973	0.0584	0.3752	42
PSC	0.0895	0.0624	0.4109	37
PTS	0.0888	0.0673	0.4309	23
SDC	0.0992	0.0632	0.3891	41
SDG	0.0933	0.0638	0.4061	38
TPP	0.0951	0.0685	0.4187	31
VBC	0.0926	0.0692	0.4277	27
VCM	0.0981	0.0888	0.4753	9
VSM	0.0822	0.0733	0.4712	10

As shown in Fig 3, from 2020 to 2021, PDN and IPA were the highest enterprises, respectively, and TMS maintained the second position. Corresponding to each year, VOS and SDG were the worst, while the CIA remained the second-lowest ranking. In 2022, PDN still lost two ranks like the previous year, while CIA dramatically jumped from 44th to the highest rank. Simultaneously, IPA dramatically dropped to the bottom of the ranking. Additionally, examining the fluctuations in ranking throughout the years reveals that the enterprises exhibiting minor variations are TCL, PBP, and PDN, particularly TCL, which consistently retains its ranking over all years. On the contrary, the firms that show the most remarkable changes are CIA, IPA, and PRC, respectively. Moreover, the differences in company rankings between 2020 and 2021 are less noticeable than from 2021 to 2022, with average standard deviations of 5.15 and 8.08, respectively.

#### 4.2.3. A comparison of EDAS and TOPSIS methods.

The weights objectively established by the CRITIC technique are consistently assigned to all indicators in both EDAS and TOPSIS. The results of the two methods generally differ in all years under examination, as displayed in Table 4. This discrepancy is understandable due to their different evaluation concepts. EDAS evaluates alternatives based on the distance to the average solution. Meanwhile, TOPSIS assesses alternatives by measuring the distance to both the positive and negative ideal solutions. The best choice should be nearest to the positive ideal solution and most distant from the negative one. However, the disparity is not notably significant, and numerous instances show that both approaches’ ranking outcomes are identical, especially for the top and worst companies. This finding demonstrates the consistency and reliability of the proposed method.

**Table 4:** Compare the companies’ rankings according to EDAS and TOPSIS over the years.

Company	2020		2021		2022	
TOPSIS	EDAS	TOPSIS	EDAS	TOPSIS	EDAS
CAV	13	14	22	27	21	16
GEX	22	37	13	39	27	41
GMD	35	44	18	34	16	39
GSP	15	13	32	18	23	32
HAH	14	25	4	7	7	31
L10	34	30	40	37	24	33
MSN	5	31	6	16	30	40
PAC	20	8	31	9	43	14
PDN	1	1	3	1	3	3
PJT	33	33	43	35	40	24
PVP	8	17	21	19	13	11
PVT	17	22	27	26	20	23
REE	11	40	17	42	10	44
SRF	40	35	42	44	35	42
STG	21	15	10	10	31	13
SVI	6	7	15	13	14	12
TCL	4	3	5	4	4	5
TDP	30	28	12	21	37	27
THI	25	19	39	36	33	38
TMS	2	2	2	2	15	9
TV2	3	4	8	14	29	37
VIP	24	41	23	28	8	6
VOS	45	45	14	41	6	29
VTB	12	9	16	11	11	8
VTO	29	21	30	15	41	22
ARM	43	24	38	24	42	18
BBS	36	34	35	30	32	36
CDN	7	11	9	6	17	21
CIA	44	32	44	43	1	1
CTB	26	23	19	31	12	25
CTT	39	26	29	23	36	19
DL1	18	16	36	45	5	43
IPA	16	36	1	8	45	45
PBP	19	18	20	5	18	4
PMP	32	27	25	22	44	28
PMS	28	12	34	17	25	10
PRC	42	38	33	33	2	2
PSC	37	43	41	40	28	15
PTS	23	42	26	38	22	34
SDC	41	29	37	25	34	30
SDG	38	6	45	29	39	20
TPP	31	39	28	32	19	35
VBC	27	20	24	20	26	26
VCM	9	10	11	12	38	17
VSM	10	5	7	3	9	7

Additionally, the average standard deviations in the ranking outcomes between the two methods were 5.72, 5.63, and 8.08 for 2020, 2021, and 2022, respectively. The significant rise in 2022 could be explained by disparate recovery and development of enterprises within the industrial goods and services sector post-COVID-19. This results in substantial variances in indicators’ value among alternatives and the potential emergence of outliers. This will exert a more significant influence on TOPSIS than on EDAS.

Notably, leading companies like PDN and TMS consistently exhibit similar rankings by EDAS and TOPSIS for many years. Likewise, the CIA achieved the top rating by both approaches in 2022. Moreover, it is remarkable that PDN, a corporation in the highest group during the observed years, possesses the most excellent inventory turnover indicator among its competitors. This index’s value of the second-ranked enterprise, TMS, also significantly exceeds that of the lowest-ranked firm, such as SDG. The inventory turnover ratio is a crucial metric that not only signifies sales performance and inventory management but also reflects the capacity to adapt to variations in market demand and reveals the enterprise’s financial health. This study’s results indicate that the inventory turnover ratio is an essential parameter for assessing the performance of firms in Vietnam’s industrial goods and service sector. The inventory turnover metric differs across various industries. For instance, this index in the automobile industry is frequently inferior to the textile field. Consequently, comparing alternatives with the average value is essential to maintain objectivity. This shows that applying EDAS and TOPSIS approaches is justified for a more comprehensive assessment.

PDN and TMS are the best corporations according to both TOPSIS and EDAS techniques, indicating that these firms exhibit exceptional performance while maintaining balance and stability across evaluation metrics. These results reflect the robust growth potential of enterprises. To attain this outcome, each enterprise employed numerous distinct tactics, and digital transformation and sustainability emerged as crucial success strategies among these organizations. In an effort to reduce emissions, PDN transferred the majority of its diesel usage to electric energy for manufacturing and business operations. Furthermore, the company built intelligent power systems to analyze electricity consumption during operations and implement electricity-saving measures. The company also installed rooftop solar power systems, generating renewable energy sources. Additionally, PDN tried to digitalize operations and administration through software such as VTOS and VSL to enhance service quality and minimize operational expenses. Besides, TMS wanted to lead in sustainable development through green services and compliance with ESG standards. The company augmented utilizing technologies to mitigate greenhouse gas emissions. Specifically, it implemented solar power systems to meet its electricity consumption requirements and alleviate strain on the national power transmission grid. TMS operated five solar power projects in various branches across Vietnam, generating an average of 7,164 MWh and decreasing CO2 emissions by approximately 5,760 tons annually. In addition, TMS promoted digital transformation to optimize operational processes and reduce costs, time, and resources while increasing labor productivity. The Transimex SPro and SFlash projects were implemented to automate 50–90% of operations, guaranteeing continuous business under all circumstances.

## 5. Conclusion

The industrial goods and services sector substantially contributes to the Vietnamese economy and draws numerous domestic and foreign investors. Performance assessment is essential for organizations in this sector. Creditors can judiciously analyze the debt repayment capacity based on evaluation results and determine whether to approve the loan. Enterprise analysts can understand their company’s current position and select strategies efficiently. The investors can identify an effective, profitable investment portfolio. Simultaneously, the evaluation results and insight gained from typical businesses are valuable information for policymakers creating support policies for this industry, particularly following the epidemic.

This study deploys a framework with three phases to evaluate and rank Vietnam’s industrial goods and services enterprises. After reviewing the literature and consulting with experts, fifteen financial ratios belonging to five groups are identified in Phase I that comprehensively reflect the enterprise’s solvency, profitability, growth, operational efficiency, and capital structure. Additionally, data from annual financial statements published on the Hanoi and HoChiMinh Stock Exchanges from 2020 to 2022 were collected, and 45 firms that satisfied the data requirements were chosen for analysis. In Phase II, the weights of the indicators are objectively determined using the CRITIC method. Finally, in Phase III, the EDAS and TOPSIS methods are simultaneously employed to assess firms based on the multi-criteria with respective weights obtained in the previous stage.

The research finding demonstrates that the CRITIC-derived weights are consistent and reliable, validated by comparing them with the results of the Statistical Variance Procedure. This result significantly contributes to the accuracy of the ranking step. In general, the ranking outcomes of the EDAS and TOPSIS techniques differ due to their distinct evaluation concepts; the former relies on the distance to the average solution, while the latter is based on the distance to the positive and negative ideal solutions. Nonetheless, there are cases in which the rankings are similar between both techniques, particularly for the highest and lowest-ranked firms such as PDN, TMS, CIA, VOS, and IPA. The gap in ranking outcomes between the two techniques during the epidemic stage is less pronounced than that observed post-pandemic. Because organizations’ varying adaptation and resilience result in considerable variations in index values among firms, this significantly impacts the outcomes of TOPSIS more than those of EDAS.

Regarding the highest ranking, PDN excelled in performance throughout COVID-19 and to be succeeded by CIA in 2022 across both methods. In terms of the least favorable companies, EDAS reports that the companies performing worst over the years were VOS, DL1, and IPA. According to TOPIS, VOS and SDG experienced the poorest outcomes in the initial two years; however, following the pandemic, IPA was replaced for this position. Additionally, ranking fluctuations of both methods over the years indicate that TCL shows relatively highest stability, while the most significant change is CIA. The variation observed between 2022 and 2021 is considerably greater than between 2021 and 2020. A noteworthy observation is that the top-ranked organizations almost have the highest inventory turnover ratio, which is suggested as a critical metric for evaluating and ranking enterprises in this sector.

In short, this study employs the proposed methodology to conduct the evaluation process objectively. It eliminates experts’ roles in setting weights and ratings, using actual enterprises’ data to evaluate and rank. Furthermore, this assessment is particularly meaningful when conducted during and after COVID-19, as it profoundly reflects the capacity of Vietnam’s industrial goods and services firms to respond to unforeseen shocks, recuperate, and develop. This study also yields several significant management implications. First, COVID-19 is both a challenge and an opportunity for organizations to change and develop, and digital transformation is one key to corporate success. In addition, sustainable development is also an essential solution to help businesses reduce costs, improve operational efficiency and reputation, and comply with international standards in standardization and global integration. Second, during and after the COVID-19 shock, short-term solvency and financial risk levels become top concerns when assessing corporate performance. Also, the inventory turnover ratio is significant to the success of firms in this industry. Third, the combination of EDAS and TOPSIS is an effective cross-validation strategy, ensuring that final decisions are not solely based on a single perspective but are supported by various independent methods. Each method possesses distinct advantages; they can offset each other’s shortcomings and yield a more comprehensive assessment. The results of TOPSIS allow for the assessment of the alternatives’ superiority. On the other hand, EDAS demonstrates stability and balance across all assessment variables. This combination is significant as it indicates the robust growth potential of the assessed firm.

While contributing to literature and application, this study has limitations like other investigations. Enterprise performance is evaluated solely on financial metrics, excluding non-financial factors such as governance indicators. Financial indexes thoroughly reflect a business’s financial health and are frequently employed to assess and rank companies in various studies. Nonetheless, governance indices are also crucial in demonstrating the efficacy of corporate governance. Consequently, integrating financial indices and governance variables is suggested for a more profound and thorough evaluation in the subsequent study. In addition, in Vietnam, ESG has recently emerged as a significant concern for enterprises, particularly within the industrial goods and services sector, as well as for stakeholders. To align with global sustainable development tendencies and satisfy export requirements, Vietnamese enterprises are proactively implementing ESG practices. Therefore, this is a significant aspect to consider when assessing firm performance in future research. Ultimately, applying the proposed framework in this research to other sectors is feasible in the upcoming work.

## Appendix A

**Table A1:** The normalized decision matrix in 2020.

Company	I1	I2	I3	I4	I5	I6	I7	I8	I9	I10	I11	I12	I13	I14	I15
CAV	0.92	0.67	0.71	0.50	0.68	0.15	0.20	0.14	0.01	0.90	0.17	0.44	1.00	0.75	0.17
GEX	0.68	0.48	0.29	0.50	0.72	0.15	0.27	0.12	0.00	0.92	0.11	0.53	0.92	0.83	0.64
GMD	0.62	0.50	0.25	0.40	0.69	0.05	0.20	0.13	0.02	0.97	0.14	0.95	0.56	0.98	0.68
GSP	0.74	0.62	0.29	0.47	0.72	0.26	0.21	0.17	0.11	0.83	0.36	0.39	0.96	0.94	0.65
HAH	0.70	0.63	0.38	0.45	0.72	0.36	0.57	0.16	0.02	0.88	0.18	0.64	0.84	0.98	0.86
L10	0.64	0.41	0.28	0.35	0.74	0.20	0.36	0.13	0.00	0.92	0.06	0.33	1.00	0.69	0.39
MSN	0.57	0.40	0.25	1.00	0.61	0.05	0.07	0.11	0.00	0.89	1.00	0.70	0.90	0.80	0.85
PAC	0.88	0.59	0.40	0.39	0.71	0.12	0.00	0.13	0.00	0.83	0.83	0.55	0.99	0.71	0.05
PDN	0.97	1.00	0.79	0.44	0.73	0.19	0.37	0.19	1.00	0.94	0.17	0.85	0.84	0.96	0.48
PJT	0.72	0.55	0.28	0.34	0.71	0.00	0.03	0.12	0.02	0.76	0.79	0.49	0.89	0.93	0.73
PVP	0.80	0.72	0.36	0.41	0.78	0.34	0.58	0.21	0.16	0.88	0.13	0.54	0.99	0.98	0.85
PVT	0.71	0.59	0.32	0.38	0.73	0.31	0.34	0.16	0.06	0.93	0.18	0.54	0.92	0.96	0.74
REE	0.76	0.67	0.59	0.49	0.70	0.27	0.40	0.14	0.00	0.93	0.09	0.80	0.86	0.97	0.88
SRF	0.65	0.43	0.25	0.33	0.68	0.12	0.20	0.11	0.00	0.96	0.02	0.41	0.89	0.67	0.08
STG	0.63	0.53	0.25	0.47	0.70	0.27	0.50	0.21	0.05	0.90	0.10	0.66	0.78	0.97	0.39
SVI	1.00	0.93	1.00	0.40	0.72	0.22	0.28	0.24	0.01	0.94	0.08	0.59	0.98	0.89	0.08
TCL	0.76	0.74	0.38	0.49	0.70	0.24	0.27	0.54	0.16	0.95	0.13	0.57	0.84	0.96	0.32
TDP	0.73	0.52	0.27	0.49	0.72	0.14	0.05	0.11	0.00	0.84	0.17	0.58	0.93	0.78	0.14
THI	0.72	0.53	0.40	0.37	0.72	0.19	0.30	0.12	0.00	0.93	0.15	0.58	0.90	0.86	0.30
TMS	0.77	0.69	0.51	0.67	0.72	0.17	0.36	0.16	0.72	0.86	0.22	0.43	0.94	0.95	0.52
TV2	0.94	0.72	0.70	0.41	0.72	0.23	0.36	0.78	0.01	0.98	0.10	0.50	0.91	0.81	0.30
VIP	0.62	0.52	0.22	0.31	0.86	0.51	0.42	0.14	0.00	0.85	0.14	0.56	0.61	0.99	1.00
VOS	0.00	0.10	0.05	0.31	0.04	0.08	0.20	0.09	0.02	0.96	0.13	0.21	0.79	0.77	0.89
VTB	0.68	0.59	0.28	0.56	0.76	0.40	0.28	0.35	0.00	0.75	0.22	0.82	0.90	0.96	0.20
VTO	0.63	0.50	0.21	0.27	0.71	0.22	0.41	0.13	0.01	0.87	0.60	0.57	0.80	0.97	0.71
ARM	0.60	0.39	0.20	0.18	0.64	0.14	0.34	0.11	0.01	0.91	0.10	0.53	0.67	0.43	0.00
BBS	0.63	0.42	0.22	0.45	0.73	0.09	0.21	0.11	0.01	0.95	0.04	0.47	0.86	0.68	0.08
CDN	0.78	0.84	0.32	0.46	0.72	1.00	0.48	0.28	0.04	0.80	0.16	0.93	0.75	1.00	0.83
CIA	0.34	0.00	0.00	0.02	0.00	0.49	1.00	0.00	0.02	0.93	0.07	0.00	0.19	0.98	0.20
CTB	0.77	0.60	0.36	0.29	0.78	0.21	0.21	0.21	0.00	0.97	0.04	0.73	0.70	0.83	0.12
CTT	0.79	0.42	0.27	0.40	0.77	0.05	0.10	0.11	0.01	0.94	0.14	0.39	0.87	0.00	0.08
DL1	0.58	0.46	0.19	0.37	0.89	0.13	0.36	0.13	0.20	0.97	0.00	0.30	0.99	0.99	0.00
IPA	0.67	0.54	0.32	0.45	0.76	0.34	0.73	0.12	0.00	0.94	0.17	1.00	0.44	0.96	0.98
PBP	0.67	0.53	0.24	0.55	0.70	0.20	0.02	0.14	0.00	0.90	0.48	0.46	0.89	0.93	0.18
PMP	0.68	0.46	0.26	0.48	0.77	0.10	0.11	0.11	0.00	0.87	0.13	0.46	0.88	0.66	0.09
PMS	0.75	0.61	0.39	0.31	0.78	0.13	0.29	0.17	0.03	0.83	0.16	0.44	0.88	0.88	0.03
PRC	0.54	0.37	0.19	0.29	0.63	0.08	0.22	0.11	0.03	0.87	0.10	0.43	0.86	0.90	0.41
PSC	0.68	0.51	0.28	0.27	0.72	0.01	0.12	0.13	0.09	0.82	0.41	0.49	0.94	0.95	0.94
PTS	0.65	0.45	0.26	0.36	0.78	0.00	0.05	0.11	0.01	0.92	0.57	0.53	0.81	0.88	0.82
SDC	0.56	0.40	0.18	0.26	0.71	0.33	0.44	0.12	0.00	0.94	0.00	0.64	0.47	0.92	0.17
SDG	0.74	0.64	0.75	0.60	0.71	0.13	0.14	0.15	0.01	0.00	0.33	0.52	0.90	0.90	0.03
TPP	0.59	0.39	0.20	0.42	0.95	0.11	0.04	0.11	0.00	0.80	0.13	0.62	0.86	0.69	0.30
VBC	0.88	0.59	0.40	0.36	0.73	0.14	0.24	0.13	0.01	0.91	0.07	0.43	0.98	0.73	0.05
VCM	0.71	0.64	0.38	0.00	1.00	0.46	0.19	1.00	0.01	1.00	0.04	0.94	0.00	0.97	0.29
VSM	0.81	0.72	0.37	0.47	0.73	0.20	0.46	0.20	0.15	0.93	0.11	0.45	0.93	0.91	0.20

**Table A2:** The symmetric linear correlation coefficient matrix in 2020.

Indicator	I1	I2	I3	I4	I5	I6	I7	I8	I9	I10	I11	I12	I13	I14	I15
I1	1.00	0.85	0.76	0.23	0.62	0.05	−0.13	0.30	0.26	−0.07	0.04	0.34	0.35	−0.02	−0.23
I2	0.85	1.00	0.80	0.29	0.56	0.25	−0.03	0.41	0.44	−0.10	−0.02	0.52	0.30	0.29	0.02
I3	0.76	0.80	1.00	0.27	0.29	−0.01	−0.07	0.31	0.34	−0.24	−0.05	0.27	0.30	0.11	−0.15
I4	0.23	0.29	0.27	1.00	0.14	−0.17	−0.27	−0.17	0.19	−0.26	0.40	0.19	0.56	0.01	0.14
I5	0.62	0.56	0.29	0.14	1.00	0.00	−0.32	0.32	0.05	−0.05	−0.03	0.51	0.17	0.00	−0.09
I6	0.05	0.25	−0.01	−0.17	0.00	1.00	0.60	0.31	−0.02	0.03	−0.28	0.33	−0.41	0.40	0.22
I7	−0.13	−0.03	−0.07	−0.27	−0.32	0.60	1.00	−0.02	0.14	0.18	−0.42	−0.01	−0.41	0.37	0.22
I8	0.30	0.41	0.31	−0.17	0.32	0.31	−0.02	1.00	0.02	0.13	−0.18	0.36	−0.32	0.18	−0.08
I9	0.26	0.44	0.34	0.19	0.05	−0.02	0.14	0.02	1.00	0.06	−0.05	0.08	0.12	0.20	0.07
I10	−0.07	−0.10	−0.24	−0.26	−0.05	0.03	0.18	0.13	0.06	1.00	−0.25	0.02	−0.19	−0.08	0.10
I11	0.04	−0.02	−0.05	0.40	−0.03	−0.28	−0.42	−0.18	−0.05	−0.25	1.00	0.04	0.22	0.06	0.30
I12	0.34	0.52	0.27	0.19	0.51	0.33	−0.01	0.36	0.08	0.02	0.04	1.00	−0.28	0.28	0.29
I13	0.35	0.30	0.30	0.56	0.17	−0.41	−0.41	−0.32	0.12	−0.19	0.22	−0.28	1.00	−0.18	−0.09
I14	−0.02	0.29	0.11	0.01	0.00	0.40	0.37	0.18	0.20	−0.08	0.06	0.28	−0.18	1.00	0.43
I15	−0.23	0.02	−0.15	0.14	−0.09	0.22	0.22	−0.08	0.07	0.10	0.30	0.29	−0.09	0.43	1.00

**Table A3:** Weighted Positive distance for all indicators in 2020.

Company	I1	I2	I3	I4	I5	I6	I7	I8	I9	I10	I11	I12	I13	I14	I15
CAV	0.015	0.011	0.063	0.013	0.000	0.000	0.000	0.000	0.000	0.000	0.000	0.012	0.000	0.007	0.071
GEX	0.000	0.000	0.000	0.013	0.001	0.000	0.000	0.000	0.000	0.000	0.000	0.002	0.000	0.001	0.000
GMD	0.000	0.000	0.000	0.000	0.000	0.000	0.000	0.000	0.000	0.000	0.000	0.000	0.025	0.000	0.000
GSP	0.003	0.006	0.000	0.009	0.001	0.013	0.000	0.000	0.041	0.003	0.064	0.018	0.000	0.000	0.000
HAH	0.000	0.007	0.006	0.006	0.001	0.044	0.078	0.000	0.000	0.000	0.000	0.000	0.000	0.000	0.000
L10	0.000	0.000	0.000	0.000	0.003	0.000	0.019	0.000	0.000	0.000	0.000	0.025	0.000	0.011	0.006
MSN	0.000	0.000	0.000	0.080	0.000	0.000	0.000	0.000	0.000	0.000	0.334	0.000	0.000	0.004	0.000
PAC	0.013	0.004	0.009	0.000	0.000	0.000	0.000	0.000	0.000	0.003	0.261	0.001	0.000	0.010	0.106
PDN	0.019	0.039	0.078	0.006	0.002	0.000	0.023	0.002	0.847	0.000	0.000	0.000	0.000	0.000	0.000
PJT	0.002	0.001	0.000	0.000	0.000	0.000	0.000	0.000	0.000	0.008	0.247	0.007	0.000	0.000	0.000
PVP	0.008	0.015	0.003	0.000	0.006	0.038	0.081	0.006	0.083	0.000	0.000	0.002	0.000	0.000	0.000
PVT	0.001	0.004	0.000	0.000	0.002	0.028	0.015	0.000	0.000	0.000	0.000	0.001	0.000	0.000	0.000
REE	0.004	0.011	0.043	0.012	0.000	0.016	0.031	0.000	0.000	0.000	0.000	0.000	0.000	0.000	0.000
SRF	0.000	0.000	0.000	0.000	0.000	0.000	0.000	0.000	0.000	0.000	0.000	0.016	0.000	0.013	0.098
STG	0.000	0.000	0.000	0.008	0.000	0.017	0.058	0.006	0.000	0.000	0.000	0.000	0.004	0.000	0.006
SVI	0.021	0.033	0.114	0.000	0.001	0.001	0.000	0.018	0.000	0.000	0.000	0.000	0.000	0.000	0.098
TCL	0.004	0.017	0.006	0.011	0.000	0.008	0.000	0.116	0.087	0.000	0.000	0.000	0.000	0.000	0.028
TDP	0.002	0.000	0.000	0.012	0.001	0.000	0.000	0.000	0.000	0.002	0.000	0.000	0.000	0.005	0.080
THI	0.002	0.000	0.010	0.000	0.001	0.000	0.002	0.000	0.000	0.000	0.000	0.000	0.000	0.000	0.032
TMS	0.005	0.013	0.028	0.036	0.001	0.000	0.018	0.000	0.593	0.002	0.003	0.014	0.000	0.000	0.000
TV2	0.017	0.015	0.061	0.001	0.001	0.006	0.019	0.194	0.000	0.000	0.000	0.006	0.000	0.003	0.032
VIP	0.000	0.000	0.000	0.000	0.012	0.085	0.035	0.000	0.000	0.002	0.000	0.000	0.020	0.000	0.000
VOS	0.000	0.000	0.000	0.000	0.000	0.000	0.000	0.000	0.000	0.000	0.000	0.039	0.002	0.006	0.000
VTB	0.000	0.004	0.000	0.021	0.004	0.055	0.000	0.052	0.000	0.009	0.001	0.000	0.000	0.000	0.063
VTO	0.000	0.000	0.000	0.000	0.000	0.002	0.032	0.000	0.000	0.001	0.163	0.000	0.002	0.000	0.000
ARM	0.000	0.000	0.000	0.000	0.000	0.000	0.013	0.000	0.000	0.000	0.000	0.002	0.015	0.030	0.119
BBS	0.000	0.000	0.000	0.006	0.002	0.000	0.000	0.000	0.000	0.000	0.000	0.009	0.000	0.012	0.098
CDN	0.006	0.025	0.000	0.008	0.001	0.229	0.053	0.031	0.000	0.005	0.000	0.000	0.007	0.000	0.000
CIA	0.000	0.000	0.000	0.000	0.000	0.082	0.194	0.000	0.000	0.000	0.000	0.061	0.061	0.000	0.063
CTB	0.006	0.005	0.003	0.000	0.006	0.000	0.000	0.008	0.000	0.000	0.000	0.000	0.011	0.001	0.085
CTT	0.007	0.000	0.000	0.000	0.005	0.000	0.000	0.000	0.000	0.000	0.000	0.018	0.000	0.060	0.098
DL1	0.000	0.000	0.000	0.000	0.014	0.000	0.020	0.000	0.120	0.000	0.000	0.028	0.000	0.000	0.119
IPA	0.000	0.000	0.000	0.007	0.004	0.038	0.122	0.000	0.000	0.000	0.000	0.000	0.037	0.000	0.000
PBP	0.000	0.000	0.000	0.019	0.000	0.000	0.000	0.000	0.000	0.000	0.113	0.010	0.000	0.000	0.067
PMP	0.000	0.000	0.000	0.010	0.005	0.000	0.000	0.000	0.000	0.001	0.000	0.011	0.000	0.013	0.093
PMS	0.004	0.006	0.008	0.000	0.006	0.000	0.000	0.000	0.000	0.003	0.000	0.013	0.000	0.000	0.111
PRC	0.000	0.000	0.000	0.000	0.000	0.000	0.000	0.000	0.000	0.001	0.000	0.014	0.000	0.000	0.002
PSC	0.000	0.000	0.000	0.000	0.001	0.000	0.000	0.000	0.016	0.004	0.082	0.007	0.000	0.000	0.000
PTS	0.000	0.000	0.000	0.000	0.006	0.000	0.000	0.000	0.000	0.000	0.154	0.003	0.001	0.000	0.000
SDC	0.000	0.000	0.000	0.000	0.000	0.033	0.042	0.000	0.000	0.000	0.000	0.000	0.034	0.000	0.071
SDG	0.003	0.008	0.070	0.026	0.000	0.000	0.000	0.000	0.000	0.061	0.049	0.004	0.000	0.000	0.111
TPP	0.000	0.000	0.000	0.002	0.019	0.000	0.000	0.000	0.000	0.005	0.000	0.000	0.000	0.011	0.032
VBC	0.013	0.003	0.010	0.000	0.002	0.000	0.000	0.000	0.000	0.000	0.000	0.013	0.000	0.009	0.106
VCM	0.001	0.008	0.007	0.000	0.023	0.073	0.000	0.264	0.000	0.000	0.000	0.000	0.080	0.000	0.037
VSM	0.008	0.015	0.005	0.009	0.002	0.000	0.048	0.004	0.076	0.000	0.000	0.012	0.000	0.000	0.063

**Table A4:** Weighted Negative distance for all indicators in 2020.

Company	I1	I2	I3	I4	I5	I6	I7	I8	I9	I10	I11	I12	I13	I14	I15
CAV	0.000	0.000	0.000	0.000	0.002	0.019	0.025	0.015	0.056	0.001	0.019	0.000	0.018	0.000	0.000
GEX	0.001	0.006	0.009	0.000	0.000	0.019	0.006	0.023	0.058	0.003	0.043	0.000	0.010	0.000	0.064
GMD	0.005	0.004	0.016	0.001	0.002	0.047	0.024	0.018	0.040	0.006	0.029	0.044	0.000	0.009	0.077
GSP	0.000	0.000	0.010	0.000	0.000	0.000	0.020	0.006	0.000	0.000	0.000	0.000	0.014	0.007	0.068
HAH	0.000	0.000	0.000	0.000	0.000	0.000	0.000	0.008	0.042	0.000	0.014	0.009	0.002	0.009	0.129
L10	0.004	0.012	0.011	0.008	0.000	0.003	0.000	0.020	0.058	0.003	0.065	0.000	0.017	0.000	0.000
MSN	0.008	0.013	0.017	0.000	0.008	0.048	0.059	0.025	0.058	0.001	0.000	0.016	0.008	0.000	0.125
PAC	0.000	0.000	0.000	0.002	0.000	0.027	0.079	0.019	0.060	0.000	0.000	0.000	0.016	0.000	0.000
PDN	0.000	0.000	0.000	0.000	0.000	0.007	0.000	0.000	0.000	0.004	0.019	0.033	0.002	0.008	0.020
PJT	0.000	0.000	0.011	0.008	0.000	0.062	0.071	0.023	0.046	0.000	0.000	0.000	0.007	0.006	0.090
PVP	0.000	0.000	0.000	0.000	0.000	0.000	0.000	0.000	0.000	0.000	0.036	0.000	0.017	0.009	0.125
PVT	0.000	0.000	0.004	0.004	0.000	0.000	0.000	0.008	0.005	0.003	0.014	0.000	0.010	0.008	0.094
REE	0.000	0.000	0.000	0.000	0.000	0.000	0.000	0.015	0.059	0.003	0.050	0.027	0.004	0.009	0.133
SRF	0.003	0.010	0.016	0.010	0.002	0.027	0.023	0.024	0.059	0.006	0.081	0.000	0.007	0.000	0.000
STG	0.005	0.001	0.017	0.000	0.000	0.000	0.000	0.000	0.019	0.002	0.046	0.012	0.000	0.009	0.000
SVI	0.000	0.000	0.000	0.001	0.000	0.000	0.002	0.000	0.052	0.004	0.054	0.003	0.016	0.003	0.000
TCL	0.000	0.000	0.000	0.000	0.000	0.000	0.005	0.000	0.000	0.004	0.033	0.001	0.002	0.008	0.000
TDP	0.000	0.003	0.013	0.000	0.000	0.020	0.066	0.024	0.062	0.000	0.017	0.003	0.011	0.000	0.000
THI	0.000	0.001	0.000	0.004	0.000	0.008	0.000	0.022	0.060	0.003	0.024	0.003	0.008	0.000	0.000
TMS	0.000	0.000	0.000	0.000	0.000	0.013	0.000	0.010	0.000	0.000	0.000	0.000	0.012	0.007	0.029
TV2	0.000	0.000	0.000	0.000	0.000	0.000	0.000	0.000	0.055	0.007	0.049	0.000	0.009	0.000	0.000
VIP	0.005	0.003	0.022	0.013	0.000	0.000	0.000	0.014	0.058	0.000	0.029	0.000	0.000	0.010	0.168
VOS	0.048	0.039	0.051	0.013	0.052	0.038	0.025	0.031	0.046	0.006	0.033	0.000	0.000	0.000	0.138
VTB	0.001	0.000	0.011	0.000	0.000	0.000	0.003	0.000	0.061	0.000	0.000	0.029	0.009	0.008	0.000
VTO	0.005	0.004	0.023	0.018	0.000	0.000	0.000	0.020	0.054	0.000	0.000	0.002	0.000	0.008	0.085
ARM	0.007	0.014	0.025	0.030	0.005	0.021	0.000	0.025	0.054	0.002	0.048	0.000	0.000	0.000	0.000
BBS	0.005	0.011	0.022	0.000	0.000	0.037	0.021	0.025	0.054	0.005	0.074	0.000	0.004	0.000	0.000
CDN	0.000	0.000	0.004	0.000	0.000	0.000	0.000	0.000	0.022	0.000	0.023	0.041	0.000	0.011	0.120
CIA	0.025	0.047	0.060	0.051	0.055	0.000	0.000	0.061	0.042	0.003	0.060	0.000	0.000	0.009	0.000
CTB	0.000	0.000	0.000	0.016	0.000	0.001	0.023	0.000	0.060	0.007	0.074	0.019	0.000	0.000	0.000
CTT	0.000	0.011	0.013	0.000	0.000	0.048	0.052	0.025	0.050	0.004	0.030	0.000	0.005	0.000	0.000
DL1	0.008	0.008	0.027	0.005	0.000	0.025	0.000	0.020	0.000	0.006	0.089	0.000	0.017	0.010	0.000
IPA	0.001	0.000	0.005	0.000	0.000	0.000	0.000	0.022	0.058	0.004	0.016	0.049	0.000	0.008	0.164
PBP	0.002	0.001	0.018	0.000	0.001	0.003	0.073	0.015	0.058	0.002	0.000	0.000	0.007	0.006	0.000
PMP	0.001	0.008	0.014	0.000	0.000	0.032	0.048	0.024	0.058	0.000	0.035	0.000	0.006	0.000	0.000
PMS	0.000	0.000	0.000	0.012	0.000	0.025	0.000	0.007	0.039	0.000	0.022	0.000	0.006	0.002	0.000
PRC	0.011	0.015	0.028	0.015	0.006	0.040	0.019	0.026	0.035	0.000	0.047	0.000	0.004	0.004	0.000
PSC	0.001	0.003	0.011	0.018	0.000	0.058	0.046	0.019	0.000	0.000	0.000	0.000	0.012	0.007	0.151
PTS	0.003	0.009	0.014	0.005	0.000	0.061	0.066	0.025	0.050	0.003	0.000	0.000	0.000	0.002	0.116
SDC	0.009	0.013	0.028	0.020	0.000	0.000	0.000	0.022	0.061	0.004	0.090	0.009	0.000	0.005	0.000
SDG	0.000	0.000	0.000	0.000	0.000	0.025	0.039	0.012	0.049	0.000	0.000	0.000	0.009	0.003	0.000
TPP	0.007	0.014	0.026	0.000	0.000	0.031	0.067	0.026	0.059	0.000	0.034	0.007	0.005	0.000	0.000
VBC	0.000	0.000	0.000	0.006	0.000	0.022	0.013	0.019	0.055	0.002	0.060	0.000	0.016	0.000	0.000
VCM	0.000	0.000	0.000	0.054	0.000	0.000	0.028	0.000	0.053	0.008	0.074	0.043	0.000	0.009	0.000
VSM	0.000	0.000	0.000	0.000	0.000	0.003	0.000	0.000	0.000	0.003	0.042	0.000	0.011	0.004	0.000

**Table A5:** The normalized decision matrix in 2020 based on TOPSIS.

Company	I1	I2	I3	I4	I5	I6	I7	I8	I9	I10	I11	I12	I13	I14	I15
CAV	0.25	0.18	0.31	0.09	−0.04	0.11	0.10	0.03	0.01	0.10	0.09	0.16	0.02	0.16	0.18
GEX	0.10	0.07	0.08	0.10	0.00	0.11	0.12	0.02	0.00	0.08	0.06	0.15	0.06	0.11	0.11
GMD	0.06	0.08	0.05	−0.01	−0.03	0.07	0.10	0.03	0.02	0.04	0.07	0.11	0.24	0.02	0.11
GSP	0.14	0.15	0.07	0.06	0.00	0.15	0.10	0.05	0.09	0.14	0.18	0.16	0.04	0.04	0.11
HAH	0.11	0.16	0.12	0.04	0.00	0.19	0.23	0.05	0.02	0.11	0.09	0.14	0.10	0.02	0.08
L10	0.08	0.04	0.07	−0.06	0.02	0.13	0.16	0.02	0.00	0.08	0.03	0.17	0.03	0.20	0.15
MSN	0.03	0.03	0.05	0.58	−0.10	0.07	0.05	0.01	0.01	0.10	0.48	0.13	0.07	0.13	0.09
PAC	0.23	0.14	0.13	−0.01	−0.01	0.10	0.03	0.02	0.00	0.14	0.40	0.15	0.03	0.19	0.19
PDN	0.28	0.37	0.36	0.04	0.01	0.12	0.16	0.07	0.77	0.06	0.09	0.12	0.10	0.03	0.13
PJT	0.13	0.11	0.07	−0.06	−0.01	0.05	0.04	0.02	0.01	0.19	0.38	0.15	0.08	0.05	0.10
PVP	0.18	0.21	0.11	0.00	0.06	0.18	0.24	0.08	0.12	0.11	0.07	0.15	0.03	0.02	0.09
PVT	0.12	0.14	0.09	−0.03	0.01	0.17	0.15	0.05	0.05	0.07	0.09	0.15	0.06	0.03	0.10
REE	0.15	0.18	0.24	0.09	−0.02	0.15	0.17	0.03	0.00	0.07	0.05	0.13	0.09	0.02	0.08
SRF	0.08	0.05	0.05	−0.07	−0.04	0.10	0.10	0.01	0.00	0.05	0.02	0.16	0.07	0.21	0.19
STG	0.07	0.10	0.05	0.06	−0.02	0.16	0.21	0.08	0.04	0.09	0.06	0.14	0.13	0.03	0.15
SVI	0.30	0.33	0.47	−0.01	0.01	0.13	0.13	0.11	0.01	0.06	0.05	0.15	0.03	0.07	0.19
TCL	0.15	0.22	0.12	0.08	−0.01	0.14	0.13	0.34	0.13	0.06	0.07	0.15	0.10	0.03	0.16
TDP	0.13	0.09	0.06	0.08	0.00	0.11	0.05	0.01	0.00	0.13	0.09	0.15	0.06	0.14	0.18
THI	0.12	0.10	0.14	−0.03	0.00	0.12	0.13	0.02	0.00	0.07	0.08	0.15	0.07	0.10	0.16
TMS	0.16	0.19	0.19	0.26	0.00	0.11	0.16	0.05	0.56	0.12	0.11	0.16	0.05	0.04	0.13
TV2	0.27	0.21	0.30	0.00	0.00	0.14	0.16	0.52	0.01	0.04	0.05	0.15	0.07	0.12	0.16
VIP	0.06	0.09	0.03	−0.09	0.13	0.25	0.18	0.04	0.00	0.13	0.07	0.15	0.21	0.01	0.07
VOS	−0.33	−0.14	−0.06	−0.10	−0.62	0.08	0.10	0.00	0.02	0.05	0.07	0.18	0.12	0.15	0.08
VTB	0.10	0.14	0.07	0.15	0.04	0.21	0.13	0.19	0.00	0.20	0.11	0.12	0.07	0.03	0.17
VTO	0.07	0.08	0.03	−0.13	−0.01	0.13	0.17	0.02	0.01	0.11	0.29	0.15	0.12	0.03	0.10
ARM	0.05	0.02	0.02	−0.21	−0.07	0.10	0.15	0.01	0.01	0.09	0.05	0.15	0.19	0.36	0.20
BBS	0.07	0.04	0.03	0.05	0.01	0.08	0.10	0.01	0.01	0.06	0.02	0.16	0.09	0.21	0.19
CDN	0.16	0.28	0.09	0.05	0.00	0.44	0.20	0.14	0.04	0.16	0.08	0.11	0.14	0.01	0.09
CIA	−0.12	−0.20	−0.09	−0.37	−0.65	0.24	0.39	−0.07	0.02	0.07	0.04	0.20	0.42	0.02	0.17
CTB	0.16	0.14	0.11	−0.12	0.05	0.13	0.10	0.09	0.00	0.04	0.03	0.13	0.17	0.11	0.18
CTT	0.17	0.04	0.06	0.00	0.04	0.07	0.06	0.01	0.01	0.07	0.07	0.16	0.09	0.63	0.19
DL1	0.03	0.06	0.02	−0.03	0.15	0.10	0.16	0.02	0.16	0.04	0.01	0.17	0.03	0.02	0.20
IPA	0.10	0.11	0.09	0.05	0.04	0.18	0.29	0.02	0.01	0.06	0.09	0.11	0.30	0.03	0.07
PBP	0.10	0.10	0.05	0.14	−0.02	0.13	0.04	0.03	0.00	0.09	0.23	0.16	0.08	0.05	0.17
PMP	0.10	0.06	0.06	0.07	0.04	0.09	0.07	0.01	0.00	0.12	0.07	0.16	0.08	0.22	0.19
PMS	0.14	0.15	0.13	−0.09	0.05	0.10	0.13	0.05	0.02	0.14	0.08	0.16	0.08	0.08	0.19
PRC	0.01	0.01	0.01	−0.11	−0.08	0.08	0.11	0.01	0.02	0.12	0.05	0.16	0.09	0.07	0.14
PSC	0.10	0.09	0.07	−0.13	0.00	0.05	0.07	0.02	0.07	0.15	0.20	0.15	0.05	0.04	0.07
PTS	0.08	0.05	0.06	−0.04	0.06	0.05	0.05	0.01	0.01	0.08	0.28	0.15	0.12	0.08	0.09
SDC	0.03	0.03	0.01	−0.14	−0.01	0.18	0.19	0.02	0.00	0.06	0.01	0.14	0.28	0.06	0.18
SDG	0.14	0.16	0.33	0.19	−0.01	0.10	0.08	0.04	0.01	0.74	0.16	0.15	0.07	0.07	0.19
TPP	0.04	0.02	0.02	0.01	0.21	0.09	0.04	0.01	0.00	0.16	0.07	0.14	0.09	0.20	0.16
VBC	0.23	0.13	0.14	−0.04	0.01	0.10	0.11	0.02	0.01	0.08	0.04	0.16	0.03	0.18	0.19
VCM	0.12	0.16	0.13	−0.39	0.25	0.23	0.09	0.69	0.01	0.02	0.02	0.11	0.51	0.03	0.16
VSM	0.18	0.21	0.12	0.06	0.01	0.13	0.19	0.08	0.12	0.07	0.06	0.16	0.06	0.06	0.17

## Supporting information

S1 Data Minimal data set-Plos one. (DOCX)
