# Supplementary material for: Performance evaluation of Vietnamese industrial goods and services during and post-COVID-19 era based on multi-criteria decision-making methods
Source: PLoS One. 2025 May 28;20(5):e0323764. doi: 10.1371/journal.pone.0323764 (PMC12119014; doi:10.1371/journal.pone.0323764)
Supplement: S1 Data — (DOCX) [file pone.0323764.s001.docx]

**MINIMAL DATA SET**

Title: **Performance evaluation of Vietnamese industrial goods and services during and post-COVID-19 era based on multi-criteria decision-making methods**

**Table S1. Data in 2020.**

| Company | Indicator | | | | | | | | | | | | | | |  |
| --- | --- | --- | --- | --- | --- | --- | --- | --- | --- | --- | --- | --- | --- | --- | --- | --- |
|  | I1 | I2 | I3 | I4 | I5 | I6 | I7 | I8 | I9 | I10 | I11 | I12 | I13 | I14 | I15 | |
| CAV | 0.2344 | 0.0814 | 6694.9005 | 0.1679 | -0.3177 | 1.2000 | 0.6922 | 5.6148 | 6.4700 | 9.2755 | 8.4400 | 0.9022 | 0.0129 | 1.9206 | 0.8800 | |
| GEX | 0.0949 | 0.0331 | 1632.0370 | 0.1714 | -0.0189 | 1.2100 | 0.8714 | 2.5629 | 5.1400 | 7.2705 | 5.8300 | 0.8536 | 0.0357 | 1.3184 | 0.5700 | |
| GMD | 0.0564 | 0.0372 | 1157.7097 | -0.0141 | -0.2718 | 0.7800 | 0.7019 | 4.4964 | 20.8700 | 4.2426 | 7.3500 | 0.6356 | 0.1311 | 0.2646 | 0.5400 | |
| GSP | 0.1313 | 0.0674 | 1583.3817 | 0.1123 | 0.0293 | 1.7000 | 0.7362 | 9.0712 | 93.2700 | 13.6873 | 17.4400 | 0.9293 | 0.0242 | 0.5018 | 0.5600 | |
| HAH | 0.1068 | 0.0705 | 2694.2779 | 0.0746 | 0.0277 | 2.1700 | 1.6500 | 8.2557 | 19.2600 | 10.2852 | 8.9900 | 0.7991 | 0.0570 | 0.2398 | 0.4200 | |
| L10 | 0.0732 | 0.0162 | 1493.8155 | -0.1024 | 0.1507 | 1.4500 | 1.1010 | 3.7310 | 4.6400 | 7.3689 | 3.3700 | 0.9627 | 0.0142 | 2.3286 | 0.7300 | |
| MSN | 0.0321 | 0.0116 | 1050.4807 | 1.0317 | -0.8921 | 0.7700 | 0.3800 | 1.6166 | 5.2900 | 9.4724 | 46.7600 | 0.7683 | 0.0394 | 1.5531 | 0.4300 | |
| PAC | 0.2125 | 0.0609 | 2909.5188 | -0.0260 | -0.0849 | 1.0800 | 0.1966 | 4.1181 | 3.4700 | 13.8368 | 38.8800 | 0.8483 | 0.0168 | 2.1929 | 0.9600 | |
| PDN | 0.2635 | 0.1635 | 7716.3104 | 0.0702 | 0.0910 | 1.3900 | 1.1410 | 12.2234 | 813.4800 | 6.1897 | 8.3600 | 0.6875 | 0.0568 | 0.3788 | 0.6700 | |
| PJT | 0.1202 | 0.0512 | 1487.9856 | -0.1066 | -0.1026 | 0.5500 | 0.2705 | 2.6146 | 15.7400 | 18.6211 | 37.3100 | 0.8774 | 0.0429 | 0.6227 | 0.5100 | |
| PVP | 0.1666 | 0.0926 | 2447.5015 | 0.0057 | 0.5217 | 2.0800 | 1.6760 | 14.1669 | 130.6200 | 10.7051 | 6.5900 | 0.8513 | 0.0153 | 0.2781 | 0.4300 | |
| PVT | 0.1119 | 0.0606 | 1993.8671 | -0.0484 | 0.0634 | 1.9300 | 1.0649 | 8.2702 | 52.2200 | 7.0334 | 8.9900 | 0.8485 | 0.0340 | 0.3853 | 0.5000 | |
| REE | 0.1397 | 0.0811 | 5250.8621 | 0.1526 | -0.1357 | 1.7400 | 1.2101 | 5.6193 | 4.4500 | 6.9442 | 5.0000 | 0.7153 | 0.0503 | 0.2820 | 0.4100 | |
| SRF | 0.0766 | 0.0206 | 1117.6827 | -0.1288 | -0.3201 | 1.0800 | 0.7090 | 2.0495 | 4.5000 | 4.5791 | 1.6900 | 0.9179 | 0.0414 | 2.4823 | 0.9400 | |
| STG | 0.0638 | 0.0462 | 1085.3203 | 0.1061 | -0.1338 | 1.7600 | 1.4634 | 13.9629 | 39.4900 | 8.8631 | 5.4300 | 0.7867 | 0.0732 | 0.3096 | 0.7300 | |
| SVI | 0.2838 | 0.1468 | 10260.3837 | -0.0093 | 0.0454 | 1.5100 | 0.9054 | 18.5759 | 10.1500 | 6.1697 | 4.5800 | 0.8267 | 0.0178 | 0.8634 | 0.9400 | |
| TCL | 0.1407 | 0.0988 | 2707.3045 | 0.1453 | -0.1218 | 1.6200 | 0.8831 | 57.4369 | 134.2300 | 5.8526 | 6.9100 | 0.8375 | 0.0560 | 0.3822 | 0.7800 | |
| TDP | 0.1223 | 0.0422 | 1352.4628 | 0.1497 | 0.0219 | 1.1900 | 0.3171 | 2.1851 | 1.3600 | 12.8606 | 8.6400 | 0.8274 | 0.0321 | 1.7075 | 0.9000 | |
| THI | 0.1162 | 0.0464 | 2991.5652 | -0.0581 | -0.0149 | 1.3800 | 0.9441 | 2.8660 | 3.6200 | 6.8917 | 7.8200 | 0.8294 | 0.0409 | 1.1468 | 0.7900 | |
| TMS | 0.1459 | 0.0867 | 4227.3330 | 0.4568 | -0.0201 | 1.2900 | 1.0953 | 7.6000 | 587.1300 | 11.9194 | 10.8400 | 0.9102 | 0.0287 | 0.4360 | 0.6500 | |
| TV2 | 0.2499 | 0.0925 | 6552.2503 | 0.0075 | 0.0200 | 1.5900 | 1.0972 | 88.2522 | 7.8500 | 3.5772 | 5.1900 | 0.8720 | 0.0377 | 1.4617 | 0.7900 | |
| VIP | 0.0585 | 0.0422 | 713.7018 | -0.1681 | 1.1158 | 2.8000 | 1.2476 | 5.9042 | 5.2600 | 12.1142 | 7.2700 | 0.8427 | 0.1174 | 0.1498 | 0.3300 | |
| VOS | -0.3080 | -0.0633 | -1337.5576 | -0.1706 | -5.4207 | 0.9100 | 0.6926 | -0.5386 | 15.7900 | 4.5515 | 6.9100 | 1.0264 | 0.0686 | 1.7934 | 0.4000 | |
| VTB | 0.0943 | 0.0615 | 1504.8215 | 0.2649 | 0.3270 | 2.3300 | 0.9023 | 32.2534 | 2.6200 | 19.2899 | 10.6200 | 0.7038 | 0.0385 | 0.3882 | 0.8600 | |
| VTO | 0.0619 | 0.0372 | 647.0852 | -0.2314 | -0.0832 | 1.5200 | 1.2247 | 3.8478 | 9.0000 | 10.9157 | 28.1600 | 0.8351 | 0.0676 | 0.3211 | 0.5200 | |
| ARM | 0.0444 | 0.0098 | 501.7839 | -0.3826 | -0.6249 | 1.1700 | 1.0448 | 1.8590 | 8.4600 | 8.3578 | 5.2800 | 0.8548 | 0.1025 | 4.2737 | 0.9900 | |
| BBS | 0.0636 | 0.0166 | 700.0000 | 0.0815 | 0.1111 | 0.9300 | 0.7301 | 1.5905 | 8.6300 | 5.4035 | 2.4200 | 0.8866 | 0.0510 | 2.4496 | 0.9400 | |
| CDN | 0.1517 | 0.1238 | 1945.7727 | 0.0978 | 0.0357 | 5.0000 | 1.4179 | 23.7692 | 37.2400 | 15.5646 | 7.9400 | 0.6464 | 0.0800 | 0.0978 | 0.4400 | |
| CIA | -0.1084 | -0.0878 | -1953.8531 | -0.6575 | -5.7134 | 2.7500 | 2.7261 | -12.5947 | 18.9600 | 7.0758 | 3.9700 | 1.1343 | 0.2298 | 0.2452 | 0.8600 | |
| CTB | 0.1502 | 0.0629 | 2442.8862 | -0.2063 | 0.4764 | 1.4800 | 0.7164 | 14.7439 | 3.6300 | 3.8166 | 2.4600 | 0.7504 | 0.0929 | 1.3260 | 0.9100 | |
| CTT | 0.1608 | 0.0186 | 1360.8835 | -0.0057 | 0.3911 | 0.7700 | 0.4461 | 1.8604 | 11.7100 | 6.5053 | 7.2000 | 0.9294 | 0.0471 | 7.3895 | 0.9400 | |
| DL1 | 0.0324 | 0.0266 | 349.8746 | -0.0618 | 1.3590 | 1.1200 | 1.1085 | 3.6452 | 164.2600 | 3.8634 | 0.7600 | 0.9772 | 0.0146 | 0.1968 | 0.9900 | |
| IPA | 0.0910 | 0.0483 | 1901.0584 | 0.0844 | 0.3441 | 2.0700 | 2.0553 | 2.8809 | 5.4100 | 5.9087 | 8.7600 | 0.6096 | 0.1640 | 0.3820 | 0.3400 | |
| PBP | 0.0898 | 0.0460 | 986.1414 | 0.2452 | -0.1631 | 1.4500 | 0.2512 | 5.6126 | 5.2100 | 8.7826 | 22.7900 | 0.8913 | 0.0434 | 0.5743 | 0.8700 | |
| PMP | 0.0973 | 0.0267 | 1249.1756 | 0.1313 | 0.3871 | 1.0100 | 0.4843 | 2.0974 | 4.9400 | 11.1502 | 6.6500 | 0.8934 | 0.0451 | 2.5777 | 0.9300 | |
| PMS | 0.1354 | 0.0667 | 2820.1913 | -0.1550 | 0.4586 | 1.1100 | 0.9246 | 8.6634 | 22.4000 | 13.7790 | 8.0400 | 0.9054 | 0.0442 | 0.9891 | 0.9700 | |
| PRC | 0.0132 | 0.0061 | 313.5968 | -0.1934 | -0.6748 | 0.8900 | 0.7467 | 1.2766 | 25.9100 | 11.1884 | 5.3600 | 0.9095 | 0.0512 | 0.8230 | 0.7200 | |
| PSC | 0.0946 | 0.0415 | 1481.0933 | -0.2304 | -0.0069 | 0.6100 | 0.4980 | 3.8860 | 70.9000 | 14.7890 | 19.4000 | 0.8772 | 0.0301 | 0.4769 | 0.3700 | |
| PTS | 0.0783 | 0.0241 | 1281.7333 | -0.0696 | 0.4889 | 0.5700 | 0.3176 | 1.7427 | 12.5400 | 7.2908 | 27.1900 | 0.8568 | 0.0651 | 0.9498 | 0.4500 | |
| SDC | 0.0253 | 0.0132 | 303.2496 | -0.2561 | -0.0862 | 2.0000 | 1.3150 | 2.8105 | 1.9300 | 6.2135 | 0.7200 | 0.7995 | 0.1565 | 0.7165 | 0.8800 | |
| SDG | 0.1280 | 0.0726 | 7162.8092 | 0.3357 | -0.0969 | 1.1200 | 0.5599 | 6.8195 | 12.8000 | 71.3763 | 15.7700 | 0.8627 | 0.0385 | 0.8559 | 0.9700 | |
| TPP | 0.0398 | 0.0102 | 432.2868 | 0.0237 | 1.7990 | 1.0200 | 0.3008 | 1.2658 | 3.9400 | 15.8553 | 6.8200 | 0.8097 | 0.0496 | 2.3555 | 0.7900 | |
| VBC | 0.2137 | 0.0598 | 2933.5125 | -0.0756 | 0.0511 | 1.1600 | 0.8055 | 4.0209 | 7.9800 | 8.1666 | 3.9300 | 0.9069 | 0.0188 | 2.0764 | 0.9600 | |
| VCM | 0.1125 | 0.0722 | 2724.5173 | -0.7000 | 2.2301 | 2.6100 | 0.6664 | 116.2001 | 9.8100 | 2.0427 | 2.4000 | 0.6389 | 0.2817 | 0.3120 | 0.8000 | |
| VSM | 0.1691 | 0.0930 | 2599.4482 | 0.1114 | 0.0506 | 1.4500 | 1.3674 | 13.1494 | 124.9700 | 7.2257 | 5.9300 | 0.9000 | 0.0310 | 0.7447 | 0.8600 | |

**Table S2. Data in 2021**

| Company | Indicator | | | | | | | | | | | | | | |  |
| --- | --- | --- | --- | --- | --- | --- | --- | --- | --- | --- | --- | --- | --- | --- | --- | --- |
|  | I1 | I2 | I3 | I4 | I5 | I6 | I7 | I8 | I9 | I10 | I11 | I12 | I13 | I14 | I15 | |
| CAV | 0.1888 | 0.0548 | 5713.0026 | -0.0082 | -0.1396 | 1.1600 | 0.2142 | 5.0561 | 3.3500 | 6.2550 | 8.1000 | 0.9073 | 0.0137 | 2.4745 | 0.9200 | |
| GEX | 0.0724 | 0.0235 | 1213.9827 | 0.5903 | 0.0696 | 1.3000 | 0.7412 | 2.8284 | 3.2400 | 7.1853 | 7.1800 | 0.8470 | 0.0451 | 1.1208 | 0.5600 | |
| GMD | 0.0898 | 0.0595 | 1911.7323 | 0.2305 | 0.3294 | 0.7500 | 0.6740 | 7.7605 | 28.3000 | 5.5910 | 8.2300 | 0.6439 | 0.0919 | 0.3212 | 0.6100 | |
| GSP | 0.1001 | 0.0515 | 964.2374 | 0.0545 | -0.1142 | 1.6500 | 1.0046 | 8.6073 | 85.7000 | 11.9522 | 12.3100 | 0.9351 | 0.0226 | 0.5006 | 0.5100 | |
| HAH | 0.2752 | 0.1673 | 8795.1271 | 0.6408 | 1.2891 | 2.0700 | 1.8011 | 21.6138 | 26.3700 | 15.1145 | 10.2600 | 0.6347 | 0.0435 | 0.3263 | 0.4700 | |
| L10 | 0.0586 | 0.0127 | 1185.5861 | -0.1074 | -0.0918 | 1.4300 | 0.9875 | 3.3045 | 4.0700 | 5.6073 | 3.2500 | 0.9687 | 0.0087 | 2.9941 | 0.7400 | |
| MSN | 0.2542 | 0.0708 | 7253.3929 | 0.1385 | 5.3602 | 1.2600 | 0.8377 | 3.4604 | 5.1800 | 8.9520 | 39.0300 | 0.7503 | 0.0459 | 0.8160 | 0.4100 | |
| PAC | 0.2176 | 0.0712 | 3366.4412 | 0.0236 | 0.1429 | 1.1700 | 0.1909 | 5.8422 | 3.8000 | 17.9360 | 46.2300 | 0.8534 | 0.0170 | 1.7810 | 0.9600 | |
| PDN | 0.2341 | 0.1490 | 7779.7042 | 0.1247 | -0.1035 | 1.3500 | 0.9886 | 17.0184 | 975.7700 | 6.8402 | 8.7100 | 0.6995 | 0.0539 | 0.3870 | 0.6700 | |
| PJT | 0.0839 | 0.0428 | 821.7007 | 0.0345 | -0.2065 | 0.8200 | 0.3948 | 2.4574 | 14.5200 | 24.7458 | 31.9400 | 0.8984 | 0.0405 | 0.3358 | 0.4400 | |
| PVP | 0.1292 | 0.0823 | 2046.7138 | -0.2278 | 0.0830 | 2.5500 | 2.3084 | 16.4706 | 66.5000 | 5.6583 | 3.1700 | 0.7921 | 0.0188 | 0.2415 | 0.4900 | |
| PVT | 0.0996 | 0.0560 | 1960.8058 | 0.0105 | -0.0055 | 1.9800 | 1.1267 | 8.2908 | 48.9500 | 7.6610 | 7.1600 | 0.8341 | 0.0372 | 0.3518 | 0.4400 | |
| REE | 0.1299 | 0.0709 | 5982.9922 | 0.0303 | 0.2096 | 1.6800 | 1.2037 | 4.6185 | 4.3200 | 5.4145 | 3.4300 | 0.6024 | 0.0638 | 0.2483 | 0.2600 | |
| SRF | 0.0531 | 0.0155 | 484.6312 | -0.3740 | 0.2787 | 1.1300 | 0.7123 | 2.0389 | 2.7300 | 2.8934 | 1.1700 | 0.9070 | 0.0470 | 2.0619 | 0.9200 | |
| STG | 0.1319 | 0.0927 | 2319.0132 | 0.4205 | 0.4744 | 1.7500 | 1.3786 | 20.7977 | 27.7300 | 12.3028 | 7.0700 | 0.8310 | 0.0465 | 0.3027 | 0.7100 | |
| SVI | 0.1534 | 0.0819 | 7178.6427 | 0.0896 | -0.4221 | 1.6500 | 0.9699 | 20.1881 | 9.0000 | 5.4142 | 4.3900 | 0.8734 | 0.0220 | 0.8093 | 0.9700 | |
| TCL | 0.1866 | 0.1236 | 3115.6725 | 0.0880 | 0.0379 | 1.5700 | 1.0505 | 92.6132 | 155.5800 | 5.8285 | 6.5900 | 0.8283 | 0.0521 | 0.4265 | 0.8000 | |
| TDP | 0.1285 | 0.0380 | 1441.9092 | 0.7735 | -0.3267 | 1.0500 | 0.4137 | 2.0246 | 2.3600 | 14.4052 | 13.2100 | 0.8731 | 0.0256 | 2.3609 | 0.8400 | |
| THI | 0.0583 | 0.0249 | 1647.0480 | -0.1497 | -0.3163 | 1.4600 | 0.6788 | 2.3179 | 2.5400 | 5.1043 | 7.5800 | 0.8668 | 0.0459 | 0.9496 | 0.7600 | |
| TMS | 0.1955 | 0.1182 | 5389.6862 | 0.8676 | 0.0337 | 1.4800 | 1.3661 | 13.6626 | 751.4900 | 15.4127 | 11.4500 | 0.9144 | 0.0250 | 0.4708 | 0.7300 | |
| TV2 | 0.2137 | 0.0697 | 5432.9388 | 0.0845 | -0.0443 | 1.1900 | 1.0047 | 60.2103 | 14.2400 | 1.9652 | 2.0300 | 0.9089 | 0.0372 | 2.2085 | 0.9800 | |
| VIP | 0.0096 | 0.0068 | 153.3086 | 0.3185 | -0.8757 | 4.0800 | 1.0160 | 1.8909 | 7.0100 | 14.9397 | 8.3100 | 0.9314 | 0.0725 | 0.1688 | 0.4700 | |
| VOS | 0.6462 | 0.1769 | 3502.1706 | 0.1113 | -3.3561 | 1.5000 | 0.8333 | 5.9698 | 11.7600 | 3.0779 | 7.7600 | 0.6818 | 0.0739 | 0.7204 | 0.4200 | |
| VTB | 0.0230 | 0.0163 | 349.1028 | -0.2914 | -0.5996 | 2.6100 | 0.9206 | 97.2726 | 3.0000 | 30.6397 | 8.5300 | 0.7539 | 0.0490 | 0.2926 | 0.8100 | |
| VTO | 0.1150 | 0.0730 | 1408.1416 | -0.1882 | 1.2768 | 2.0800 | 1.8270 | 7.8704 | 8.7000 | 13.5874 | 20.5500 | 0.8627 | 0.0718 | 0.3152 | 0.5900 | |
| ARM | 0.0531 | 0.0123 | 558.7237 | 0.0679 | 0.0427 | 1.3300 | 1.1502 | 2.1621 | 10.2100 | 8.8574 | 4.5600 | 0.8772 | 0.0833 | 2.2482 | 0.9800 | |
| BBS | 0.0940 | 0.0262 | 1350.0000 | 0.0669 | 0.4197 | 1.0000 | 0.8070 | 2.0196 | 9.3100 | 6.3652 | 2.4400 | 0.9182 | 0.0205 | 2.5433 | 0.9900 | |
| CDN | 0.1681 | 0.1383 | 2258.8682 | 0.1929 | -0.0283 | 4.7200 | 1.4373 | 30.8479 | 33.2000 | 17.2008 | 9.1000 | 0.6404 | 0.0849 | 0.1153 | 0.5600 | |
| CIA | -0.1385 | -0.1076 | -2020.3291 | -0.5882 | 1.6045 | 2.5700 | 2.4440 | -14.2105 | 8.6600 | 3.9825 | 1.8900 | 1.6495 | 0.4585 | 0.2374 | 0.8300 | |
| CTB | 0.1115 | 0.0399 | 1844.4308 | 0.3204 | -0.4254 | 2.0200 | 0.6188 | 3.5238 | 2.4800 | 5.3441 | 3.7700 | 0.8639 | 0.0672 | 1.3157 | 0.6200 | |
| CTT | 0.1603 | 0.0185 | 1281.2108 | 0.3867 | -0.2456 | 0.8100 | 0.5641 | 1.8811 | 15.9600 | 8.4021 | 8.6300 | 0.9481 | 0.0357 | 7.1209 | 0.9500 | |
| DL1 | 0.0400 | 0.0271 | 447.2558 | -0.4661 | 1.4015 | 1.4500 | 1.3798 | 4.6802 | 6.8000 | 1.8416 | 3.6600 | 0.9405 | 0.0175 | 0.4332 | 0.5800 | |
| IPA | 0.5505 | 0.2489 | 8742.4770 | 0.0757 | 6.9964 | 4.0300 | 4.0123 | 8.7361 | 14.7900 | 8.1690 | 6.6500 | 0.4559 | 0.1501 | 0.2761 | 0.2200 | |
| PBP | 0.0923 | 0.0564 | 1027.2682 | 0.1798 | -0.1170 | 1.6300 | 0.5646 | 13.5689 | 7.4200 | 14.2599 | 69.2200 | 0.9099 | 0.0437 | 0.5727 | 0.9300 | |
| PMP | 0.1029 | 0.0270 | 1359.8840 | 0.4743 | -0.2616 | 1.0300 | 0.4451 | 2.2564 | 5.8600 | 16.5994 | 9.9100 | 0.9047 | 0.0429 | 2.7458 | 0.9600 | |
| PMS | 0.1505 | 0.0751 | 3074.6111 | 0.0017 | 0.1415 | 1.1700 | 0.6563 | 11.9812 | 15.1400 | 12.7554 | 8.2500 | 0.9086 | 0.0405 | 0.9793 | 0.9900 | |
| PRC | 0.0469 | 0.0223 | 1110.6557 | -0.0115 | 2.5828 | 0.9900 | 0.7756 | 2.0230 | 22.6400 | 14.7693 | 5.8200 | 0.9068 | 0.0509 | 0.6833 | 0.6400 | |
| PSC | 0.0641 | 0.0288 | 927.7951 | 0.0362 | -0.3613 | 0.6500 | 0.5385 | 3.1299 | 82.7800 | 15.0031 | 21.7000 | 0.9019 | 0.0279 | 0.4681 | 0.4100 | |
| PTS | 0.1201 | 0.0412 | 1872.8192 | 0.0881 | 0.4717 | 0.7100 | 0.4680 | 2.5986 | 16.0200 | 8.9367 | 25.5500 | 0.8587 | 0.0670 | 0.7864 | 0.4600 | |
| SDC | 0.0447 | 0.0259 | 681.4649 | -0.0916 | 0.9509 | 2.2600 | 1.5252 | 8.2059 | 1.9900 | 6.7125 | 0.7200 | 0.7811 | 0.2079 | 0.5599 | 0.8700 | |
| SDG | 0.0426 | 0.0224 | 1769.1150 | -0.1551 | -0.6076 | 1.2100 | 0.5382 | 2.7249 | 8.7800 | 27.6482 | 7.4600 | 0.8984 | 0.0369 | 0.8666 | 0.9400 | |
| TPP | 0.0299 | 0.0078 | 274.6364 | 0.4783 | -0.3554 | 1.0900 | 0.6049 | 1.1935 | 4.5100 | 14.7270 | 6.7500 | 0.8178 | 0.0300 | 2.2036 | 0.7900 | |
| VBC | 0.2039 | 0.0656 | 2998.6319 | 0.1220 | -0.0945 | 1.2300 | 0.8309 | 4.2350 | 9.1200 | 8.1878 | 4.5800 | 0.9158 | 0.0174 | 2.0028 | 0.9800 | |
| VCM | 0.0135 | 0.0100 | 323.6994 | 0.0668 | -0.8886 | 2.7600 | 0.7282 | 71.6186 | 53.7500 | 5.8470 | 3.9500 | 0.7961 | 0.1924 | 0.2696 | 0.9300 | |
| VSM | 0.2234 | 0.1221 | 2599.4482 | 0.4212 | 0.0882 | 1.5200 | 1.4652 | 47.0138 | 157.6200 | 7.9527 | 6.3200 | 0.8915 | 0.0328 | 0.7612 | 0.9500 | |

**Table S3. Data in 2022**

| Company | Indicator | | | | | | | | | | | | | | |  |
| --- | --- | --- | --- | --- | --- | --- | --- | --- | --- | --- | --- | --- | --- | --- | --- | --- |
|  | I1 | I2 | I3 | I4 | I5 | I6 | I7 | I8 | I9 | I10 | I11 | I12 | I13 | I14 | I15 | |
| CAV | 0.2117 | 0.0677 | 6670.9283 | 0.0822 | 0.0574 | 1.2800 | 0.3277 | 5.1064 | 3.1500 | 10.8899 | 13.9900 | 0.8973 | 0.0113 | 1.4100 | 0.9000 | |
| GEX | 0.0178 | 0.0065 | 429.1464 | 0.1217 | -0.1805 | 1.1500 | 0.5506 | 2.5262 | 2.4500 | 7.2967 | 9.7200 | 0.7987 | 0.0520 | 0.8210 | 0.5500 | |
| GMD | 0.1326 | 0.0837 | 3297.9040 | 0.2158 | 0.3256 | 0.8200 | 0.7094 | 11.0069 | 28.8300 | 3.5768 | 7.9700 | 0.5593 | 0.1345 | 0.4040 | 0.6300 | |
| GSP | 0.1166 | 0.0582 | 1382.4585 | 0.1307 | 0.2814 | 1.5400 | 0.7035 | 4.8376 | 69.0000 | 11.5648 | 11.8100 | 0.9106 | 0.0284 | 0.4478 | 0.4300 | |
| HAH | 0.3433 | 0.1985 | 11684.4269 | 0.6394 | 0.1530 | 1.9200 | 1.5315 | 23.2578 | 28.1200 | 15.6969 | 13.5800 | 0.5565 | 0.0355 | 0.3153 | 0.4200 | |
| L10 | 0.0665 | 0.0146 | 1421.8521 | -0.0336 | 0.1892 | 1.5600 | 1.2900 | 3.8770 | 4.4400 | 5.3760 | 3.2600 | 0.9634 | 0.0189 | 2.1775 | 0.7000 | |
| MSN | 0.0903 | 0.0267 | 2505.3971 | -0.1494 | -0.4467 | 0.7300 | 0.4759 | 2.0618 | 4.0000 | 7.1125 | 29.2100 | 0.7239 | 0.0506 | 1.7829 | 0.6200 | |
| PAC | 0.1763 | 0.0645 | 3086.9756 | 0.0865 | -0.1638 | 1.1500 | 0.1852 | 4.7835 | 4.0500 | 24.3283 | 39.1500 | 0.8578 | 0.0184 | 1.5638 | 0.9700 | |
| PDN | 0.3016 | 0.1968 | 11676.7004 | 0.1961 | 0.2178 | 1.6000 | 1.1604 | 21.4334 | 677.0700 | 7.2140 | 10.4100 | 0.6579 | 0.0519 | 0.3538 | 0.7100 | |
| PJT | 0.0760 | 0.0448 | 843.4014 | 0.3104 | -0.2064 | 0.9900 | 0.4340 | 3.0175 | 15.5900 | 25.5783 | 48.9500 | 0.9221 | 0.0376 | 0.3552 | 0.5700 | |
| PVP | 0.1355 | 0.0888 | 2291.8271 | 0.1973 | -0.0647 | 2.4800 | 2.1365 | 18.4515 | 100.4600 | 4.8576 | 3.2200 | 0.9317 | 0.0208 | 0.3500 | 0.6300 | |
| PVT | 0.1145 | 0.0641 | 2568.2649 | 0.2127 | 0.1421 | 2.0600 | 1.0377 | 7.7935 | 46.0300 | 9.9288 | 8.3200 | 0.8171 | 0.0451 | 0.3812 | 0.4900 | |
| REE | 0.1514 | 0.0819 | 7554.5381 | 0.6127 | 0.0208 | 2.1000 | 1.4572 | 5.3937 | 4.5100 | 7.6998 | 3.9900 | 0.5380 | 0.0616 | 0.2128 | 0.2800 | |
| SRF | -0.2818 | -0.0789 | -3972.6405 | 0.2742 | -4.2280 | 1.1300 | 0.7905 | -2.2646 | 3.9300 | 3.4962 | 1.6200 | 1.0158 | 0.1353 | 2.8471 | 0.9400 | |
| STG | 0.1189 | 0.0872 | 2426.4438 | -0.0857 | 0.1244 | 2.0500 | 1.6280 | 18.5878 | 19.2100 | 12.4127 | 7.0800 | 0.8288 | 0.0491 | 0.2282 | 0.7300 | |
| SVI | 0.1713 | 0.0980 | 9085.7964 | 0.0223 | 0.2380 | 1.8900 | 0.8685 | 29.7120 | 8.2900 | 5.0236 | 4.8700 | 0.8579 | 0.0231 | 0.6673 | 0.9900 | |
| TCL | 0.2196 | 0.1462 | 4130.1243 | 0.1428 | -0.0372 | 1.8400 | 1.3235 | 124.8327 | 136.6100 | 8.5085 | 7.5200 | 0.8297 | 0.0500 | 0.3614 | 0.7700 | |
| TDP | 0.1222 | 0.0309 | 1390.1287 | 0.3310 | -0.1887 | 1.0500 | 0.4401 | 1.7721 | 3.0700 | 15.7823 | 10.7100 | 0.8863 | 0.0259 | 2.7280 | 0.8900 | |
| THI | 0.0319 | 0.0160 | 897.5641 | -0.3491 | -0.2710 | 2.2800 | 1.0470 | 1.6741 | 1.5800 | 6.3013 | 7.6100 | 0.8771 | 0.0595 | 0.4406 | 0.6200 | |
| TMS | 0.1730 | 0.1169 | 6241.0063 | -0.4278 | 0.9204 | 2.0300 | 1.5651 | 14.3284 | 241.8000 | 11.2366 | 7.1500 | 0.8493 | 0.0477 | 0.2071 | 0.6000 | |
| TV2 | 0.0387 | 0.0142 | 783.2003 | -0.6357 | -0.4658 | 1.3100 | 1.1447 | 11.3887 | 5.8600 | 0.6545 | 0.6200 | 0.8594 | 0.1129 | 1.1766 | 0.9800 | |
| VIP | 0.2080 | 0.1689 | 3245.5427 | 0.2855 | 17.3453 | 7.0200 | 1.9618 | 28.2443 | 9.3500 | 34.6845 | 8.6000 | 0.8247 | 0.0560 | 0.0979 | 0.7800 | |
| VOS | 0.3846 | 0.1799 | 3485.3002 | 0.6999 | -0.4146 | 1.9000 | 1.0065 | 11.1539 | 14.4900 | 4.6478 | 11.5600 | 0.6952 | 0.0437 | 0.4131 | 0.5500 | |
| VTB | 0.0967 | 0.0740 | 1448.0480 | -0.2424 | 2.8820 | 4.6300 | 1.5882 | 134.4936 | 3.8500 | 64.8360 | 9.7300 | 0.7010 | 0.0633 | 0.1508 | 0.6000 | |
| VTO | 0.0638 | 0.0425 | 697.5443 | 0.2473 | -0.5452 | 2.9700 | 2.5355 | 4.6269 | 10.5400 | 23.9879 | 16.7600 | 0.8338 | 0.0729 | 0.2205 | 0.4700 | |
| ARM | 0.0658 | 0.0198 | 762.7197 | -0.0578 | 0.3040 | 1.3100 | 1.1847 | 7.0188 | 12.5400 | 14.7191 | 5.0500 | 0.8457 | 0.1136 | 2.2983 | 0.9800 | |
| BBS | 0.0712 | 0.0212 | 1252.0998 | -0.1432 | -0.1068 | 1.0000 | 0.7435 | 1.9043 | 7.2400 | 6.5113 | 2.2200 | 0.9074 | 0.0302 | 2.1240 | 0.9900 | |
| CDN | 0.1771 | 0.1412 | 2525.3936 | 0.1089 | 0.0098 | 3.8600 | 1.2676 | 38.9289 | 32.7600 | 12.3639 | 9.5800 | 0.6139 | 0.1126 | 0.1459 | 0.4900 | |
| CIA | 0.2255 | 0.1960 | 3324.0727 | 1.5557 | -1.4618 | 23.2800 | 23.5110 | 21.3169 | 14.5300 | 9.2598 | 8.2200 | 0.9710 | 0.1633 | 0.0359 | 0.8800 | |
| CTB | 0.2024 | 0.0619 | 3617.5484 | 0.6728 | 0.1567 | 1.1000 | 0.5559 | 5.6718 | 3.0200 | 5.0861 | 4.0800 | 0.8593 | 0.0440 | 2.0204 | 0.8400 | |
| CTT | 0.1641 | 0.0175 | 1631.8031 | 0.0923 | -0.0428 | 0.8800 | 0.6537 | 2.0669 | 18.3600 | 7.3809 | 6.9400 | 0.9437 | 0.0361 | 9.0231 | 0.9700 | |
| DL1 | 0.0366 | 0.0183 | 426.4276 | 1.7736 | -0.4691 | 1.2000 | 1.1342 | 1.5576 | 6.3000 | 5.0495 | 15.3900 | 0.5529 | 0.0939 | 0.4513 | 0.3700 | |
| IPA | 0.0251 | 0.0110 | 442.6531 | 0.1043 | -0.9243 | 3.9500 | 3.9319 | 1.1701 | 12.4200 | 25.4230 | 9.2100 | 0.4687 | 0.2124 | 0.2201 | 0.1700 | |
| PBP | 0.1055 | 0.0560 | 1406.8275 | 0.0775 | 0.0804 | 1.3800 | 0.1712 | 21.6074 | 8.7000 | 16.5108 | 177.9500 | 0.9265 | 0.0355 | 1.1277 | 0.9900 | |
| PMP | 0.1069 | 0.0289 | 1445.3828 | -0.0748 | 0.1488 | 1.0400 | 0.5715 | 2.2576 | 5.6400 | 18.1817 | 8.1700 | 0.8942 | 0.0502 | 2.4715 | 0.9700 | |
| PMS | 0.1460 | 0.0731 | 3328.5276 | 0.7142 | -0.4206 | 1.2500 | 0.7489 | 10.1021 | 18.5600 | 23.5647 | 17.5900 | 0.9418 | 0.0242 | 0.9988 | 0.9900 | |
| PRC | 0.9391 | 0.6031 | 41537.2864 | 0.2401 | 29.1575 | 3.9000 | 2.9253 | 39.4931 | 24.6700 | 27.6727 | 6.2700 | 0.9330 | 0.0602 | 0.2712 | 0.7400 | |
| PSC | 0.0470 | 0.0222 | 752.4167 | 0.7897 | -0.6007 | 0.6800 | 0.5274 | 3.5628 | 119.8900 | 28.0731 | 33.4800 | 0.9114 | 0.0289 | 0.5981 | 0.5500 | |
| PTS | 0.0925 | 0.0346 | 1419.8190 | 0.2695 | -0.3655 | 0.6800 | 0.4978 | 2.4100 | 24.4500 | 10.7421 | 28.4800 | 0.8844 | 0.0572 | 0.8684 | 0.5300 | |
| SDC | 0.0321 | 0.0193 | 424.4308 | -0.0265 | -0.2629 | 2.1700 | 1.3595 | 9.2170 | 1.8700 | 6.7510 | 0.7200 | 0.7631 | 0.2104 | 0.6066 | 0.8800 | |
| SDG | 0.0706 | 0.0360 | 3192.4628 | 0.0979 | 0.6094 | 1.2500 | 0.7043 | 4.0850 | 8.3400 | 16.0613 | 6.4700 | 0.8891 | 0.0303 | 0.9401 | 0.9400 | |
| TPP | 0.0357 | 0.0092 | 326.5751 | 0.5371 | 0.1604 | 1.0600 | 0.5243 | 1.4683 | 4.5900 | 7.0342 | 6.5500 | 0.8274 | 0.0303 | 2.4893 | 0.8500 | |
| VBC | 0.1865 | 0.0610 | 2907.9262 | -0.0306 | 0.0004 | 1.2200 | 0.7986 | 4.1323 | 7.6500 | 7.7947 | 4.2400 | 0.9069 | 0.0186 | 2.0325 | 0.9900 | |
| VCM | 0.0033 | 0.0024 | 75.4730 | -0.3731 | -0.6281 | 2.4700 | 0.8207 | 16.8782 | 39.4600 | 4.5713 | 3.0400 | 0.7972 | 0.3512 | 0.3447 | 0.8500 | |
| VSM | 0.2173 | 0.1208 | 4471.6761 | 0.2512 | -0.0885 | 1.6300 | 1.5568 | 24.1060 | 224.4700 | 9.7772 | 6.7000 | 0.9058 | 0.0257 | 0.6229 | 0.7800 | |

**Table S4. The standard deviation, amount of information content, and weight of indicators in 2020 based on CRITIC.**

| Indicator | Standard deviation | Amount of information content | Weight |
| --- | --- | --- | --- |
| I1 | 0.1617 | 1.723 | 0.048 |
| I2 | 0.1779 | 1.680 | 0.047 |
| I3 | 0.1917 | 2.126 | 0.060 |
| I4 | 0.1555 | 1.936 | 0.054 |
| I5 | 0.1659 | 1.964 | 0.055 |
| I6 | 0.1733 | 2.204 | 0.062 |
| I7 | 0.1976 | 2.800 | 0.079 |
| I8 | 0.1747 | 2.174 | 0.061 |
| I9 | 0.1821 | 2.208 | 0.062 |
| I10 | 0.1464 | 2.157 | 0.061 |
| I11 | 0.2243 | 3.192 | 0.090 |
| I12 | 0.1963 | 2.176 | 0.061 |
| I13 | 0.2052 | 2.844 | 0.080 |
| I14 | 0.1793 | 2.141 | 0.060 |
| I15 | 0.3307 | 4.249 | 0.119 |

**Table S5. The normalized decision matrix in 2021 based on CRITIC.**

| Company | Indicator | | | | | | | | | | | | | | |  |
| --- | --- | --- | --- | --- | --- | --- | --- | --- | --- | --- | --- | --- | --- | --- | --- | --- |
|  | I1 | I2 | I3 | I4 | I5 | I6 | I7 | I8 | I9 | I10 | I11 | I12 | I13 | I14 | I15 | |
| CAV | 0.4171 | 0.4555 | 0.7150 | 0.3984 | 0.3107 | 0.1253 | 0.0061 | 0.1728 | 0.0014 | 0.8467 | 0.1077 | 0.6219 | 0.9887 | 0.6632 | 0.0909 | |
| GEX | 0.2688 | 0.3677 | 0.2990 | 0.8095 | 0.3309 | 0.1597 | 0.1440 | 0.1528 | 0.0013 | 0.8144 | 0.0943 | 0.6724 | 0.9190 | 0.8565 | 0.5584 | |
| GMD | 0.2909 | 0.4687 | 0.3636 | 0.5624 | 0.3560 | 0.0246 | 0.1264 | 0.1971 | 0.0270 | 0.8698 | 0.1096 | 0.8425 | 0.8149 | 0.9706 | 0.4935 | |
| GSP | 0.3041 | 0.4463 | 0.2760 | 0.4415 | 0.3131 | 0.2457 | 0.2129 | 0.2047 | 0.0860 | 0.6489 | 0.1692 | 0.5985 | 0.9690 | 0.9450 | 0.6234 | |
| HAH | 0.5272 | 0.7711 | 1.0000 | 0.8442 | 0.4487 | 0.3489 | 0.4214 | 0.3213 | 0.0250 | 0.5391 | 0.1393 | 0.8502 | 0.9226 | 0.9699 | 0.6753 | |
| L10 | 0.2512 | 0.3374 | 0.2964 | 0.3303 | 0.3153 | 0.1916 | 0.2085 | 0.1571 | 0.0021 | 0.8692 | 0.0369 | 0.5703 | 1.0000 | 0.5891 | 0.3247 | |
| MSN | 0.5004 | 0.5004 | 0.8575 | 0.4992 | 0.8419 | 0.1499 | 0.1693 | 0.1585 | 0.0033 | 0.7531 | 0.5593 | 0.7534 | 0.9173 | 0.9000 | 0.7532 | |
| PAC | 0.4538 | 0.5015 | 0.4981 | 0.4202 | 0.3380 | 0.1278 | 0.0000 | 0.1799 | 0.0019 | 0.4411 | 0.6644 | 0.6669 | 0.9814 | 0.7622 | 0.0390 | |
| PDN | 0.4748 | 0.7198 | 0.9061 | 0.4897 | 0.3142 | 0.1720 | 0.2088 | 0.2801 | 1.0000 | 0.8264 | 0.1166 | 0.7959 | 0.8995 | 0.9612 | 0.4156 | |
| PJT | 0.2834 | 0.4219 | 0.2628 | 0.4277 | 0.3042 | 0.0418 | 0.0534 | 0.1495 | 0.0129 | 0.2047 | 0.4558 | 0.6293 | 0.9291 | 0.9685 | 0.7143 | |
| PVP | 0.3411 | 0.5327 | 0.3760 | 0.2475 | 0.3322 | 0.4668 | 0.5541 | 0.2752 | 0.0662 | 0.8675 | 0.0358 | 0.7183 | 0.9774 | 0.9820 | 0.6494 | |
| PVT | 0.3034 | 0.4589 | 0.3681 | 0.4112 | 0.3236 | 0.3268 | 0.2449 | 0.2018 | 0.0482 | 0.7979 | 0.0940 | 0.6832 | 0.9366 | 0.9662 | 0.7143 | |
| REE | 0.3420 | 0.5007 | 0.7400 | 0.4249 | 0.3444 | 0.2531 | 0.2650 | 0.1689 | 0.0024 | 0.8759 | 0.0396 | 0.8773 | 0.8775 | 0.9810 | 0.9481 | |
| SRF | 0.2442 | 0.3453 | 0.2316 | 0.1471 | 0.3511 | 0.1179 | 0.1364 | 0.1458 | 0.0008 | 0.9635 | 0.0066 | 0.6221 | 0.9147 | 0.7221 | 0.0909 | |
| STG | 0.3446 | 0.5619 | 0.4012 | 0.6929 | 0.3700 | 0.2703 | 0.3108 | 0.3140 | 0.0264 | 0.6367 | 0.0927 | 0.6857 | 0.9159 | 0.9732 | 0.3636 | |
| SVI | 0.3720 | 0.5316 | 0.8505 | 0.4656 | 0.2834 | 0.2457 | 0.2039 | 0.3086 | 0.0072 | 0.8759 | 0.0536 | 0.6502 | 0.9703 | 0.9009 | 0.0260 | |
| TCL | 0.4143 | 0.6485 | 0.4749 | 0.4645 | 0.3278 | 0.2260 | 0.2249 | 0.9582 | 0.1577 | 0.8616 | 0.0857 | 0.6880 | 0.9035 | 0.9556 | 0.2468 | |
| TDP | 0.3403 | 0.4084 | 0.3201 | 0.9354 | 0.2926 | 0.0983 | 0.0583 | 0.1456 | 0.0004 | 0.5637 | 0.1823 | 0.6504 | 0.9622 | 0.6795 | 0.1948 | |
| THI | 0.2508 | 0.3717 | 0.3391 | 0.3012 | 0.2936 | 0.1990 | 0.1277 | 0.1483 | 0.0006 | 0.8867 | 0.1001 | 0.6558 | 0.9172 | 0.8809 | 0.2987 | |
| TMS | 0.4256 | 0.6334 | 0.6851 | 1.0000 | 0.3274 | 0.2039 | 0.3075 | 0.2500 | 0.7697 | 0.5288 | 0.1566 | 0.6159 | 0.9636 | 0.9492 | 0.3377 | |
| TV2 | 0.4488 | 0.4973 | 0.6891 | 0.4621 | 0.3199 | 0.1327 | 0.2130 | 0.6676 | 0.0126 | 0.9957 | 0.0191 | 0.6205 | 0.9365 | 0.7012 | 0.0130 | |
| VIP | 0.1887 | 0.3209 | 0.2010 | 0.6228 | 0.2396 | 0.8428 | 0.2159 | 0.1444 | 0.0052 | 0.5452 | 0.1108 | 0.6016 | 0.8580 | 0.9924 | 0.6753 | |
| VOS | 1.0000 | 0.7980 | 0.5106 | 0.4805 | 0.0000 | 0.2088 | 0.1681 | 0.1810 | 0.0100 | 0.9571 | 0.1028 | 0.8108 | 0.8550 | 0.9136 | 0.7403 | |
| VTB | 0.2058 | 0.3475 | 0.2191 | 0.2038 | 0.2663 | 0.4816 | 0.1910 | 1.0000 | 0.0010 | 0.0000 | 0.1140 | 0.7503 | 0.9104 | 0.9747 | 0.2338 | |
| VTO | 0.3231 | 0.5066 | 0.3170 | 0.2747 | 0.4475 | 0.3514 | 0.4281 | 0.1981 | 0.0069 | 0.5921 | 0.2895 | 0.6592 | 0.8596 | 0.9715 | 0.5195 | |
| ARM | 0.2442 | 0.3363 | 0.2385 | 0.4507 | 0.3283 | 0.1671 | 0.2510 | 0.1469 | 0.0084 | 0.7564 | 0.0561 | 0.6470 | 0.8341 | 0.6955 | 0.0130 | |
| BBS | 0.2963 | 0.3753 | 0.3116 | 0.4500 | 0.3647 | 0.0860 | 0.1612 | 0.1456 | 0.0075 | 0.8429 | 0.0251 | 0.6127 | 0.9737 | 0.6534 | 0.0000 | |
| CDN | 0.3907 | 0.6898 | 0.3957 | 0.5365 | 0.3214 | 1.0000 | 0.3262 | 0.4042 | 0.0321 | 0.4667 | 0.1223 | 0.8454 | 0.8304 | 1.0000 | 0.5584 | |
| CIA | 0.0000 | 0.0000 | 0.0000 | 0.0000 | 0.4792 | 0.4717 | 0.5896 | 0.0000 | 0.0068 | 0.9257 | 0.0171 | 0.0000 | 0.0000 | 0.9826 | 0.2078 | |
| CTB | 0.3186 | 0.4137 | 0.3573 | 0.6241 | 0.2831 | 0.3366 | 0.1120 | 0.1591 | 0.0005 | 0.8784 | 0.0445 | 0.6582 | 0.8698 | 0.8286 | 0.4805 | |
| CTT | 0.3808 | 0.3537 | 0.3053 | 0.6697 | 0.3005 | 0.0393 | 0.0977 | 0.1443 | 0.0143 | 0.7722 | 0.1155 | 0.5877 | 0.9400 | 0.0000 | 0.0519 | |
| DL1 | 0.2275 | 0.3778 | 0.2282 | 0.0838 | 0.4596 | 0.1966 | 0.3111 | 0.1694 | 0.0049 | 1.0000 | 0.0429 | 0.5940 | 0.9804 | 0.9546 | 0.5325 | |
| IPA | 0.8780 | 1.0000 | 0.9951 | 0.4560 | 1.0000 | 0.8305 | 1.0000 | 0.2058 | 0.0131 | 0.7803 | 0.0866 | 1.0000 | 0.6856 | 0.9770 | 1.0000 | |
| PBP | 0.2941 | 0.4600 | 0.2818 | 0.5275 | 0.3129 | 0.2408 | 0.0978 | 0.2492 | 0.0056 | 0.5688 | 1.0000 | 0.6196 | 0.9222 | 0.9347 | 0.0779 | |
| PMP | 0.3076 | 0.3776 | 0.3125 | 0.7298 | 0.2989 | 0.0934 | 0.0665 | 0.1477 | 0.0040 | 0.4875 | 0.1342 | 0.6240 | 0.9239 | 0.6245 | 0.0390 | |
| PMS | 0.3683 | 0.5125 | 0.4711 | 0.4052 | 0.3378 | 0.1278 | 0.1218 | 0.2349 | 0.0135 | 0.6210 | 0.1099 | 0.6207 | 0.9293 | 0.8767 | 0.0000 | |
| PRC | 0.2363 | 0.3644 | 0.2895 | 0.3961 | 0.5737 | 0.0835 | 0.1530 | 0.1456 | 0.0212 | 0.5511 | 0.0745 | 0.6222 | 0.9060 | 0.9189 | 0.4545 | |
| PSC | 0.2582 | 0.3826 | 0.2726 | 0.4289 | 0.2893 | 0.0000 | 0.0910 | 0.1555 | 0.0830 | 0.5430 | 0.3063 | 0.6263 | 0.9572 | 0.9496 | 0.7532 | |
| PTS | 0.3296 | 0.4174 | 0.3600 | 0.4645 | 0.3697 | 0.0147 | 0.0725 | 0.1508 | 0.0144 | 0.7536 | 0.3625 | 0.6625 | 0.8703 | 0.9042 | 0.6883 | |
| SDC | 0.2335 | 0.3745 | 0.2498 | 0.3411 | 0.4160 | 0.3956 | 0.3492 | 0.2011 | 0.0000 | 0.8309 | 0.0000 | 0.7276 | 0.5572 | 0.9365 | 0.1558 | |
| SDG | 0.2308 | 0.3647 | 0.3504 | 0.2975 | 0.2655 | 0.1376 | 0.0909 | 0.1519 | 0.0070 | 0.1039 | 0.0984 | 0.6292 | 0.9372 | 0.8927 | 0.0649 | |
| TPP | 0.2146 | 0.3237 | 0.2122 | 0.7326 | 0.2899 | 0.1081 | 0.1083 | 0.1382 | 0.0026 | 0.5526 | 0.0880 | 0.6968 | 0.9525 | 0.7019 | 0.2597 | |
| VBC | 0.4363 | 0.4858 | 0.4641 | 0.4879 | 0.3151 | 0.1425 | 0.1675 | 0.1655 | 0.0073 | 0.7796 | 0.0564 | 0.6147 | 0.9806 | 0.7306 | 0.0130 | |
| VCM | 0.1937 | 0.3299 | 0.2167 | 0.4499 | 0.2383 | 0.5184 | 0.1406 | 0.7699 | 0.0532 | 0.8609 | 0.0472 | 0.7150 | 0.5915 | 0.9780 | 0.0779 | |
| VSM | 0.4612 | 0.6443 | 0.4271 | 0.6933 | 0.3327 | 0.2138 | 0.3335 | 0.5492 | 0.1598 | 0.7878 | 0.0818 | 0.6351 | 0.9463 | 0.9078 | 0.0519 | |

**Table S6. The symmetric linear correlation coefficient matrix in 2021 based on CRITIC.**

| Indicator | I1 | I2 | I3 | I4 | I5 | I6 | I7 | I8 | I9 | I10 | I11 | I12 | I13 | I14 | I15 |
| --- | --- | --- | --- | --- | --- | --- | --- | --- | --- | --- | --- | --- | --- | --- | --- |
| I1 | 1 | 0.8686 | 0.6920 | 0.2731 | 0.1731 | 0.0889 | 0.2732 | 0.0653 | 0.1549 | 0.1635 | 0.0768 | 0.5807 | 0.2258 | 0.0103 | 0.2994 |
| I2 | 0.8686 | 1 | 0.7612 | 0.3312 | 0.2314 | 0.2522 | 0.3983 | 0.2328 | 0.3329 | 0.0427 | 0.0592 | 0.7276 | 0.2968 | 0.2603 | 0.3599 |
| I3 | 0.6920 | 0.7612 | 1 | 0.2856 | 0.3683 | 0.0498 | 0.2632 | 0.0992 | 0.3567 | 0.1442 | 0.0379 | 0.5498 | 0.2502 | 0.1137 | 0.2417 |
| I4 | 0.2731 | 0.3312 | 0.2856 | 1 | -0.1172 | -0.1007 | -0.1793 | -0.0111 | 0.2554 | -0.1634 | 0.0950 | 0.3025 | 0.3355 | -0.1937 | 0.0172 |
| I5 | 0.1731 | 0.2314 | 0.3683 | -0.1172 | 1 | 0.2129 | 0.5910 | -0.1463 | -0.0646 | 0.0751 | 0.1137 | 0.1556 | -0.2273 | 0.1498 | 0.3240 |
| I6 | 0.0889 | 0.2522 | 0.0498 | -0.1007 | 0.2129 | 1 | 0.6286 | 0.2402 | -0.0556 | -0.0694 | -0.1702 | 0.1945 | -0.3942 | 0.4147 | 0.3010 |
| I7 | 0.2732 | 0.3983 | 0.2632 | -0.1793 | 0.5910 | 0.6286 | 1 | 0.0444 | 0.0670 | 0.2021 | -0.2783 | 0.1162 | -0.4558 | 0.3551 | 0.3764 |
| I8 | 0.0653 | 0.2328 | 0.0992 | -0.0111 | -0.1463 | 0.2402 | 0.0444 | 1 | 0.1093 | -0.1248 | -0.1108 | 0.2378 | 0.0500 | 0.2085 | -0.1973 |
| I9 | 0.1549 | 0.3329 | 0.3567 | 0.2554 | -0.0646 | -0.0556 | 0.0670 | 0.1093 | 1 | 0.0124 | -0.0352 | 0.0860 | 0.0737 | 0.1641 | 0.0264 |
| I10 | 0.1635 | 0.0427 | 0.1442 | -0.1634 | 0.0751 | -0.0694 | 0.2021 | -0.1248 | 0.0124 | 1 | -0.3635 | -0.0774 | -0.1977 | -0.1264 | 0.0111 |
| I11 | 0.0768 | 0.0592 | 0.0379 | 0.0950 | 0.1137 | -0.1702 | -0.2783 | -0.1108 | -0.0352 | -0.3635 | 1 | 0.0255 | 0.1737 | 0.0912 | 0.0706 |
| I12 | 0.5807 | 0.7276 | 0.5498 | 0.3025 | 0.1556 | 0.1945 | 0.1162 | 0.2378 | 0.0860 | -0.0774 | 0.0255 | 1 | 0.4297 | 0.1928 | 0.4165 |
| I13 | 0.2258 | 0.2968 | 0.2502 | 0.3355 | -0.2273 | -0.3942 | -0.4558 | 0.0500 | 0.0737 | -0.1977 | 0.1737 | 0.4297 | 1 | -0.2474 | -0.0096 |
| I14 | 0.0103 | 0.2603 | 0.1137 | -0.1937 | 0.1498 | 0.4147 | 0.3551 | 0.2085 | 0.1641 | -0.1264 | 0.0912 | 0.1928 | -0.2474 | 1 | 0.4893 |
| I15 | 0.2994 | 0.3599 | 0.2417 | 0.0172 | 0.3240 | 0.3010 | 0.3764 | -0.1973 | 0.0264 | 0.0111 | 0.0706 | 0.4165 | -0.0096 | 0.4893 | 1 |

**Table S7. The standard deviation, amount of information content, and weight of indicators in 2021 based on CRITIC.**

| Indicator | Standard deviation | Amount of information content | Weight |
| --- | --- | --- | --- |
| I1 | 0.1635 | 1.644 | 0.048 |
| I2 | 0.1626 | 1.438 | 0.042 |
| I3 | 0.2304 | 2.255 | 0.065 |
| I4 | 0.2048 | 2.636 | 0.076 |
| I5 | 0.1486 | 1.807 | 0.052 |
| I6 | 0.2160 | 2.680 | 0.077 |
| I7 | 0.1754 | 2.034 | 0.059 |
| I8 | 0.2095 | 2.786 | 0.081 |
| I9 | 0.1850 | 2.315 | 0.067 |
| I10 | 0.2250 | 3.256 | 0.094 |
| I11 | 0.1895 | 2.694 | 0.078 |
| I12 | 0.1363 | 1.372 | 0.040 |
| I13 | 0.1626 | 2.227 | 0.064 |
| I14 | 0.1771 | 2.146 | 0.062 |
| I15 | 0.2921 | 3.293 | 0.095 |

**Table S8. The normalized decision matrix in 2022 based on CRITIC.**

| Company | Indicator | | | | | | | | | | | | | | |  |
| --- | --- | --- | --- | --- | --- | --- | --- | --- | --- | --- | --- | --- | --- | --- | --- | --- |
|  | I1 | I2 | I3 | I4 | I5 | I6 | I7 | I8 | I9 | I10 | I11 | I12 | I13 | I14 | I15 | |
| CAV | 0.4042 | 0.2150 | 0.2339 | 0.2980 | 0.1284 | 0.0265 | 0.0067 | 0.0539 | 0.0023 | 0.8405 | 0.0754 | 0.2166 | 1.0000 | 0.8471 | 0.1098 | |
| GEX | 0.2454 | 0.1252 | 0.0967 | 0.3144 | 0.1212 | 0.0208 | 0.0163 | 0.0350 | 0.0013 | 0.8965 | 0.0513 | 0.3968 | 0.8802 | 0.9126 | 0.5366 | |
| GMD | 0.3394 | 0.2384 | 0.1598 | 0.3534 | 0.1364 | 0.0062 | 0.0231 | 0.0970 | 0.0403 | 0.9545 | 0.0414 | 0.8345 | 0.6374 | 0.9590 | 0.4390 | |
| GSP | 0.3263 | 0.2010 | 0.1177 | 0.3181 | 0.1351 | 0.0381 | 0.0228 | 0.0519 | 0.0998 | 0.8300 | 0.0631 | 0.1924 | 0.9496 | 0.9542 | 0.6829 | |
| HAH | 0.5120 | 0.4067 | 0.3440 | 0.5293 | 0.1312 | 0.0549 | 0.0583 | 0.1866 | 0.0393 | 0.7656 | 0.0731 | 0.8396 | 0.9287 | 0.9689 | 0.6951 | |
| L10 | 0.2853 | 0.1371 | 0.1185 | 0.2499 | 0.1323 | 0.0389 | 0.0479 | 0.0449 | 0.0042 | 0.9264 | 0.0149 | 0.0957 | 0.9777 | 0.7617 | 0.3537 | |
| MSN | 0.3048 | 0.1548 | 0.1423 | 0.2019 | 0.1133 | 0.0022 | 0.0131 | 0.0316 | 0.0036 | 0.8994 | 0.1612 | 0.5335 | 0.8843 | 0.8056 | 0.4512 | |
| PAC | 0.3752 | 0.2103 | 0.1551 | 0.2997 | 0.1217 | 0.0208 | 0.0006 | 0.0515 | 0.0037 | 0.6311 | 0.2173 | 0.2888 | 0.9790 | 0.8300 | 0.0244 | |
| PDN | 0.4778 | 0.4043 | 0.3439 | 0.3453 | 0.1332 | 0.0407 | 0.0424 | 0.1733 | 1.0000 | 0.8978 | 0.0552 | 0.6542 | 0.8805 | 0.9646 | 0.3415 | |
| PJT | 0.2931 | 0.1814 | 0.1058 | 0.3927 | 0.1205 | 0.0137 | 0.0113 | 0.0386 | 0.0207 | 0.6117 | 0.2725 | 0.1714 | 0.9225 | 0.9645 | 0.5122 | |
| PVP | 0.3418 | 0.2459 | 0.1377 | 0.3457 | 0.1247 | 0.0796 | 0.0842 | 0.1515 | 0.1464 | 0.9345 | 0.0147 | 0.1537 | 0.9720 | 0.9650 | 0.4390 | |
| PVT | 0.3246 | 0.2097 | 0.1437 | 0.3522 | 0.1309 | 0.0611 | 0.0371 | 0.0735 | 0.0658 | 0.8555 | 0.0434 | 0.3633 | 0.9006 | 0.9616 | 0.6098 | |
| REE | 0.3548 | 0.2358 | 0.2533 | 0.5181 | 0.1273 | 0.0628 | 0.0551 | 0.0560 | 0.0043 | 0.8902 | 0.0190 | 0.8733 | 0.8518 | 0.9803 | 0.8659 | |
| SRF | 0.0000 | 0.0000 | 0.0000 | 0.3777 | 0.0000 | 0.0199 | 0.0265 | 0.0000 | 0.0035 | 0.9557 | 0.0056 | 0.0000 | 0.6351 | 0.6872 | 0.0610 | |
| STG | 0.3282 | 0.2435 | 0.1406 | 0.2283 | 0.1304 | 0.0606 | 0.0624 | 0.1525 | 0.0261 | 0.8168 | 0.0364 | 0.3419 | 0.8886 | 0.9786 | 0.3171 | |
| SVI | 0.3711 | 0.2594 | 0.2869 | 0.2731 | 0.1338 | 0.0535 | 0.0299 | 0.2338 | 0.0099 | 0.9319 | 0.0240 | 0.2886 | 0.9651 | 0.9297 | 0.0000 | |
| TCL | 0.4107 | 0.3301 | 0.1780 | 0.3231 | 0.1255 | 0.0513 | 0.0494 | 0.9294 | 0.1999 | 0.8776 | 0.0389 | 0.3401 | 0.8860 | 0.9638 | 0.2683 | |
| TDP | 0.3309 | 0.1610 | 0.1178 | 0.4012 | 0.1210 | 0.0164 | 0.0115 | 0.0295 | 0.0022 | 0.7643 | 0.0569 | 0.2367 | 0.9568 | 0.7005 | 0.1220 | |
| THI | 0.2569 | 0.1391 | 0.1070 | 0.1190 | 0.1185 | 0.0708 | 0.0375 | 0.0288 | 0.0000 | 0.9120 | 0.0394 | 0.2536 | 0.8581 | 0.9550 | 0.4512 | |
| TMS | 0.3725 | 0.2871 | 0.2244 | 0.0863 | 0.1542 | 0.0597 | 0.0597 | 0.1213 | 0.3556 | 0.8351 | 0.0368 | 0.3045 | 0.8929 | 0.9810 | 0.4756 | |
| TV2 | 0.2625 | 0.1365 | 0.1045 | 0.0000 | 0.1127 | 0.0279 | 0.0417 | 0.0998 | 0.0063 | 1.0000 | 0.0000 | 0.2859 | 0.7011 | 0.8731 | 0.0122 | |
| VIP | 0.4012 | 0.3633 | 0.1586 | 0.3823 | 0.6462 | 0.2805 | 0.0767 | 0.2231 | 0.0115 | 0.4698 | 0.0450 | 0.3493 | 0.8684 | 0.9931 | 0.2561 | |
| VOS | 0.5458 | 0.3795 | 0.1639 | 0.5544 | 0.1142 | 0.0540 | 0.0358 | 0.0981 | 0.0191 | 0.9378 | 0.0617 | 0.5860 | 0.9046 | 0.9580 | 0.5366 | |
| VTB | 0.3100 | 0.2242 | 0.1191 | 0.1633 | 0.2130 | 0.1748 | 0.0607 | 1.0000 | 0.0034 | 0.0000 | 0.0514 | 0.5755 | 0.8471 | 0.9872 | 0.4756 | |
| VTO | 0.2831 | 0.1780 | 0.1026 | 0.3665 | 0.1103 | 0.1013 | 0.1013 | 0.0504 | 0.0133 | 0.6364 | 0.0910 | 0.3327 | 0.8186 | 0.9795 | 0.6341 | |
| ARM | 0.2847 | 0.1447 | 0.1041 | 0.2399 | 0.1357 | 0.0279 | 0.0434 | 0.0679 | 0.0162 | 0.7809 | 0.0250 | 0.3109 | 0.6991 | 0.7483 | 0.0122 | |
| BBS | 0.2891 | 0.1468 | 0.1148 | 0.2044 | 0.1234 | 0.0142 | 0.0245 | 0.0305 | 0.0084 | 0.9087 | 0.0090 | 0.1982 | 0.9443 | 0.7677 | 0.0000 | |
| CDN | 0.3759 | 0.3227 | 0.1428 | 0.3091 | 0.1269 | 0.1407 | 0.0470 | 0.3012 | 0.0462 | 0.8176 | 0.0505 | 0.7347 | 0.7019 | 0.9878 | 0.6098 | |
| CIA | 0.4155 | 0.4031 | 0.1603 | 0.9096 | 0.0829 | 1.0000 | 1.0000 | 0.1724 | 0.0192 | 0.8659 | 0.0429 | 0.0819 | 0.5527 | 1.0000 | 0.1341 | |
| CTB | 0.3966 | 0.2065 | 0.1668 | 0.5431 | 0.1313 | 0.0186 | 0.0165 | 0.0580 | 0.0021 | 0.9310 | 0.0195 | 0.2861 | 0.9036 | 0.7792 | 0.1829 | |
| CTT | 0.3652 | 0.1413 | 0.1231 | 0.3022 | 0.1254 | 0.0088 | 0.0207 | 0.0317 | 0.0248 | 0.8952 | 0.0356 | 0.1319 | 0.9270 | 0.0000 | 0.0244 | |
| DL1 | 0.2608 | 0.1425 | 0.0967 | 1.0000 | 0.1126 | 0.0230 | 0.0413 | 0.0279 | 0.0070 | 0.9315 | 0.0833 | 0.8462 | 0.7569 | 0.9538 | 0.7561 | |
| IPA | 0.2514 | 0.1318 | 0.0970 | 0.3071 | 0.0990 | 0.1447 | 0.1611 | 0.0251 | 0.0160 | 0.6141 | 0.0484 | 1.0000 | 0.4083 | 0.9795 | 1.0000 | |
| PBP | 0.3172 | 0.1978 | 0.1182 | 0.2960 | 0.1290 | 0.0310 | 0.0000 | 0.1746 | 0.0105 | 0.7529 | 1.0000 | 0.1632 | 0.9286 | 0.8785 | 0.0000 | |
| PMP | 0.3184 | 0.1581 | 0.1191 | 0.2328 | 0.1311 | 0.0159 | 0.0172 | 0.0331 | 0.0060 | 0.7269 | 0.0426 | 0.2224 | 0.8855 | 0.7290 | 0.0244 | |
| PMS | 0.3504 | 0.2229 | 0.1604 | 0.5603 | 0.1140 | 0.0252 | 0.0248 | 0.0904 | 0.0251 | 0.6430 | 0.0957 | 0.1354 | 0.9620 | 0.8929 | 0.0000 | |
| PRC | 1.0000 | 1.0000 | 1.0000 | 0.3635 | 1.0000 | 0.1425 | 0.1180 | 0.3053 | 0.0342 | 0.5790 | 0.0319 | 0.1514 | 0.8559 | 0.9738 | 0.3049 | |
| PSC | 0.2693 | 0.1482 | 0.1038 | 0.5916 | 0.1086 | 0.0000 | 0.0153 | 0.0426 | 0.1751 | 0.5728 | 0.1853 | 0.1909 | 0.9480 | 0.9374 | 0.5366 | |
| PTS | 0.3066 | 0.1664 | 0.1185 | 0.3757 | 0.1157 | 0.0000 | 0.0140 | 0.0342 | 0.0339 | 0.8428 | 0.1571 | 0.2402 | 0.8648 | 0.9074 | 0.5610 | |
| SDC | 0.2571 | 0.1440 | 0.0966 | 0.2529 | 0.1188 | 0.0659 | 0.0509 | 0.0840 | 0.0004 | 0.9050 | 0.0006 | 0.4620 | 0.4142 | 0.9365 | 0.1341 | |
| SDG | 0.2886 | 0.1685 | 0.1574 | 0.3045 | 0.1449 | 0.0252 | 0.0228 | 0.0464 | 0.0100 | 0.7599 | 0.0330 | 0.2317 | 0.9440 | 0.8994 | 0.0610 | |
| TPP | 0.2601 | 0.1292 | 0.0945 | 0.4868 | 0.1314 | 0.0168 | 0.0151 | 0.0273 | 0.0045 | 0.9006 | 0.0334 | 0.3443 | 0.9439 | 0.7270 | 0.1707 | |
| VBC | 0.3836 | 0.2051 | 0.1512 | 0.2512 | 0.1267 | 0.0239 | 0.0269 | 0.0468 | 0.0090 | 0.8887 | 0.0204 | 0.1991 | 0.9783 | 0.7778 | 0.0000 | |
| VCM | 0.2335 | 0.1192 | 0.0890 | 0.1090 | 0.1078 | 0.0792 | 0.0278 | 0.1400 | 0.0561 | 0.9390 | 0.0136 | 0.3996 | 0.0000 | 0.9656 | 0.1707 | |
| VSM | 0.4088 | 0.2928 | 0.1855 | 0.3681 | 0.1240 | 0.0420 | 0.0594 | 0.1928 | 0.3300 | 0.8579 | 0.0343 | 0.2011 | 0.9576 | 0.9347 | 0.2561 | |

**Table S9. The symmetric linear correlation coefficient matrix in 2022 based on CRITIC.**

| Indicator | I1 | I2 | I3 | I4 | I5 | I6 | I7 | I8 | I9 | I10 | I11 | I12 | I13 | I14 | I15 |
| --- | --- | --- | --- | --- | --- | --- | --- | --- | --- | --- | --- | --- | --- | --- | --- |
| I1 | 1 | 0.9500 | 0.9040 | 0.1401 | 0.7334 | 0.1746 | 0.1456 | 0.2572 | 0.1934 | -0.1652 | -0.0328 | 0.0487 | 0.2486 | 0.1590 | 0.0340 |
| I2 | 0.9500 | 1 | 0.9166 | 0.1694 | 0.7868 | 0.3150 | 0.2659 | 0.3434 | 0.2373 | -0.2115 | -0.0506 | 0.0563 | 0.1296 | 0.3101 | 0.0830 |
| I3 | 0.9040 | 0.9166 | 1 | 0.0500 | 0.7871 | 0.0836 | 0.0716 | 0.1979 | 0.2024 | -0.1440 | -0.0748 | 0.0296 | 0.1580 | 0.1803 | 0.0319 |
| I4 | 0.1401 | 0.1694 | 0.0500 | 1 | -0.0241 | 0.3956 | 0.4462 | -0.1225 | -0.0435 | 0.0314 | 0.0354 | 0.1637 | 0.0709 | 0.1112 | 0.2497 |
| I5 | 0.7334 | 0.7868 | 0.7871 | -0.0241 | 1 | 0.1412 | 0.0201 | 0.2252 | -0.0307 | -0.3859 | -0.0530 | -0.0777 | 0.0829 | 0.1489 | -0.0150 |
| I6 | 0.1746 | 0.3150 | 0.0836 | 0.3956 | 0.1412 | 1 | 0.9656 | 0.1864 | -0.0512 | -0.1419 | -0.0832 | -0.0720 | -0.2922 | 0.2402 | -0.0054 |
| I7 | 0.1456 | 0.2659 | 0.0716 | 0.4462 | 0.0201 | 0.9656 | 1 | 0.0796 | -0.0225 | -0.0073 | -0.0974 | -0.0966 | -0.2798 | 0.1822 | -0.0077 |
| I8 | 0.2572 | 0.3434 | 0.1979 | -0.1225 | 0.2252 | 0.1864 | 0.0796 | 1 | 0.1372 | -0.4849 | -0.0170 | 0.1245 | 0.0037 | 0.2637 | 0.0244 |
| I9 | 0.1934 | 0.2373 | 0.2024 | -0.0435 | -0.0307 | -0.0512 | -0.0225 | 0.1372 | 1 | 0.0955 | -0.0514 | 0.1083 | 0.0882 | 0.1659 | 0.0820 |
| I10 | -0.1652 | -0.2115 | -0.1440 | 0.0314 | -0.3859 | -0.1419 | -0.0073 | -0.4849 | 0.0955 | 1 | -0.1700 | -0.0435 | -0.1123 | -0.1947 | -0.1359 |
| I11 | -0.0328 | -0.0506 | -0.0748 | 0.0354 | -0.0530 | -0.0832 | -0.0974 | -0.0170 | -0.0514 | -0.1700 | 1 | -0.1225 | 0.1652 | 0.0368 | -0.0855 |
| I12 | 0.0487 | 0.0563 | 0.0296 | 0.1637 | -0.0777 | -0.0720 | -0.0966 | 0.1245 | 0.1083 | -0.0435 | -0.1225 | 1 | -0.3095 | 0.3428 | 0.6468 |
| I13 | 0.2486 | 0.1296 | 0.1580 | 0.0709 | 0.0829 | -0.2922 | -0.2798 | 0.0037 | 0.0882 | -0.1123 | 0.1652 | -0.3095 | 1 | -0.1798 | -0.0824 |
| I14 | 0.1590 | 0.3101 | 0.1803 | 0.1112 | 0.1489 | 0.2402 | 0.1822 | 0.2637 | 0.1659 | -0.1947 | 0.0368 | 0.3428 | -0.1798 | 1 | 0.4598 |
| I15 | 0.0340 | 0.0830 | 0.0319 | 0.2497 | -0.0150 | -0.0054 | -0.0077 | 0.0244 | 0.0820 | -0.1359 | -0.0855 | 0.6468 | -0.0824 | 0.4598 | 1 |

**Table S10. The standard deviation, amount of information content, and weight of indicators in 2022 based on CRITIC.**

| Indicator | Standard deviation | Amount of information content | Weight |
| --- | --- | --- | --- |
| I1 | 0.1316 | 1.344 | 0.041 |
| I2 | 0.1469 | 1.425 | 0.043 |
| I3 | 0.1425 | 1.512 | 0.046 |
| I4 | 0.1834 | 2.261 | 0.069 |
| I5 | 0.1529 | 1.783 | 0.054 |
| I6 | 0.1511 | 1.835 | 0.056 |
| I7 | 0.1465 | 1.807 | 0.055 |
| I8 | 0.1966 | 2.513 | 0.077 |
| I9 | 0.1629 | 2.100 | 0.064 |
| I10 | 0.1749 | 2.810 | 0.086 |
| I11 | 0.1515 | 2.212 | 0.067 |
| I12 | 0.2398 | 3.166 | 0.097 |
| I13 | 0.1893 | 2.709 | 0.083 |
| I14 | 0.1628 | 1.917 | 0.058 |
| I15 | 0.2681 | 3.410 | 0.104 |

**Table S11. The criteria's average solution in 2020 based on EDAS.**

| Company | Indicator | | | | | | | | | | | | | | |  |
| --- | --- | --- | --- | --- | --- | --- | --- | --- | --- | --- | --- | --- | --- | --- | --- | --- |
|  | I1 | I2 | I3 | I4 | I5 | I6 | I7 | I8 | I9 | I10 | I11 | I12 | I13 | I14 | I15 | |
| CAV | 0.9165 | 0.6733 | 0.7081 | 0.5012 | 0.6793 | 0.1461 | 0.1960 | 0.1414 | 0.0063 | 0.8957 | 0.1677 | 0.4424 | 1.0000 | 0.7500 | 0.1667 | |
| GEX | 0.6808 | 0.4811 | 0.2936 | 0.5032 | 0.7169 | 0.1483 | 0.2668 | 0.1177 | 0.0047 | 0.9246 | 0.1110 | 0.5349 | 0.9153 | 0.8326 | 0.6364 | |
| GMD | 0.6157 | 0.4974 | 0.2547 | 0.3961 | 0.6850 | 0.0517 | 0.1997 | 0.1327 | 0.0240 | 0.9683 | 0.1440 | 0.9505 | 0.5606 | 0.9771 | 0.6818 | |
| GSP | 0.7423 | 0.6176 | 0.2896 | 0.4691 | 0.7229 | 0.2584 | 0.2133 | 0.1682 | 0.1132 | 0.8320 | 0.3632 | 0.3907 | 0.9580 | 0.9446 | 0.6515 | |
| HAH | 0.7009 | 0.6299 | 0.3806 | 0.4473 | 0.7227 | 0.3640 | 0.5746 | 0.1619 | 0.0220 | 0.8811 | 0.1796 | 0.6388 | 0.8362 | 0.9805 | 0.8636 | |
| L10 | 0.6441 | 0.4138 | 0.2823 | 0.3451 | 0.7382 | 0.2022 | 0.3575 | 0.1268 | 0.0040 | 0.9232 | 0.0576 | 0.3271 | 0.9951 | 0.6941 | 0.3939 | |
| MSN | 0.5747 | 0.3955 | 0.2460 | 1.0000 | 0.6069 | 0.0494 | 0.0725 | 0.1103 | 0.0048 | 0.8928 | 1.0000 | 0.6975 | 0.9016 | 0.8004 | 0.8485 | |
| PAC | 0.8795 | 0.5917 | 0.3982 | 0.3892 | 0.7086 | 0.1191 | 0.0000 | 0.1298 | 0.0026 | 0.8299 | 0.8288 | 0.5450 | 0.9855 | 0.7127 | 0.0455 | |
| PDN | 0.9657 | 1.0000 | 0.7917 | 0.4448 | 0.7307 | 0.1888 | 0.3734 | 0.1927 | 1.0000 | 0.9402 | 0.1659 | 0.8516 | 0.8368 | 0.9615 | 0.4848 | |
| PJT | 0.7236 | 0.5531 | 0.2818 | 0.3427 | 0.7063 | 0.0000 | 0.0292 | 0.1181 | 0.0177 | 0.7609 | 0.7947 | 0.4897 | 0.8886 | 0.9280 | 0.7273 | |
| PVP | 0.8020 | 0.7179 | 0.3603 | 0.4075 | 0.7849 | 0.3438 | 0.5849 | 0.2078 | 0.1592 | 0.8751 | 0.1275 | 0.5394 | 0.9912 | 0.9753 | 0.8485 | |
| PVT | 0.7095 | 0.5905 | 0.3232 | 0.3763 | 0.7272 | 0.3101 | 0.3433 | 0.1620 | 0.0626 | 0.9280 | 0.1796 | 0.5447 | 0.9218 | 0.9606 | 0.7424 | |
| REE | 0.7565 | 0.6721 | 0.5899 | 0.4924 | 0.7022 | 0.2674 | 0.4007 | 0.1414 | 0.0038 | 0.9293 | 0.0930 | 0.7987 | 0.8610 | 0.9747 | 0.8788 | |
| SRF | 0.6499 | 0.4314 | 0.2515 | 0.3298 | 0.6790 | 0.1191 | 0.2026 | 0.1137 | 0.0039 | 0.9634 | 0.0211 | 0.4125 | 0.8941 | 0.6730 | 0.0758 | |
| STG | 0.6283 | 0.5332 | 0.2488 | 0.4655 | 0.7024 | 0.2719 | 0.5008 | 0.2062 | 0.0470 | 0.9016 | 0.1023 | 0.6624 | 0.7757 | 0.9710 | 0.3939 | |
| SVI | 1.0000 | 0.9335 | 1.0000 | 0.3989 | 0.7250 | 0.2157 | 0.2802 | 0.2420 | 0.0108 | 0.9405 | 0.0838 | 0.5863 | 0.9820 | 0.8950 | 0.0758 | |
| TCL | 0.7582 | 0.7425 | 0.3816 | 0.4881 | 0.7039 | 0.2404 | 0.2714 | 0.5437 | 0.1636 | 0.9450 | 0.1344 | 0.5657 | 0.8397 | 0.9610 | 0.3182 | |
| TDP | 0.7271 | 0.5173 | 0.2707 | 0.4906 | 0.7220 | 0.1438 | 0.0476 | 0.1148 | 0.0000 | 0.8440 | 0.1720 | 0.5850 | 0.9288 | 0.7792 | 0.1364 | |
| THI | 0.7168 | 0.5340 | 0.4049 | 0.3707 | 0.7174 | 0.1865 | 0.2955 | 0.1200 | 0.0028 | 0.9301 | 0.1542 | 0.5812 | 0.8959 | 0.8561 | 0.3030 | |
| TMS | 0.7670 | 0.6944 | 0.5061 | 0.6680 | 0.7167 | 0.1663 | 0.3553 | 0.1568 | 0.7213 | 0.8575 | 0.2198 | 0.4271 | 0.9412 | 0.9536 | 0.5152 | |
| TV2 | 0.9427 | 0.7175 | 0.6964 | 0.4086 | 0.7218 | 0.2337 | 0.3561 | 0.7830 | 0.0080 | 0.9779 | 0.0971 | 0.5000 | 0.9079 | 0.8130 | 0.3030 | |
| VIP | 0.6193 | 0.5173 | 0.2184 | 0.3071 | 0.8597 | 0.5056 | 0.4155 | 0.1436 | 0.0048 | 0.8547 | 0.1423 | 0.5557 | 0.6116 | 0.9929 | 1.0000 | |
| VOS | 0.0000 | 0.0975 | 0.0505 | 0.3057 | 0.0368 | 0.0809 | 0.1961 | 0.0936 | 0.0178 | 0.9638 | 0.1344 | 0.2057 | 0.7930 | 0.7675 | 0.8939 | |
| VTB | 0.6798 | 0.5941 | 0.2832 | 0.5572 | 0.7604 | 0.4000 | 0.2790 | 0.3482 | 0.0016 | 0.7512 | 0.2150 | 0.8205 | 0.9048 | 0.9602 | 0.1970 | |
| VTO | 0.6250 | 0.4974 | 0.2129 | 0.2706 | 0.7088 | 0.2180 | 0.4064 | 0.1277 | 0.0094 | 0.8720 | 0.5960 | 0.5702 | 0.7967 | 0.9694 | 0.7121 | |
| ARM | 0.5955 | 0.3884 | 0.2010 | 0.1833 | 0.6406 | 0.1393 | 0.3353 | 0.1122 | 0.0087 | 0.9089 | 0.0990 | 0.5326 | 0.6669 | 0.4273 | 0.0000 | |
| BBS | 0.6279 | 0.4154 | 0.2173 | 0.4513 | 0.7332 | 0.0854 | 0.2109 | 0.1101 | 0.0090 | 0.9515 | 0.0369 | 0.4721 | 0.8583 | 0.6775 | 0.0758 | |
| CDN | 0.7768 | 0.8420 | 0.3193 | 0.4607 | 0.7238 | 1.0000 | 0.4828 | 0.2823 | 0.0442 | 0.8050 | 0.1568 | 0.9299 | 0.7505 | 1.0000 | 0.8333 | |
| CIA | 0.3373 | 0.0000 | 0.0000 | 0.0246 | 0.0000 | 0.4944 | 1.0000 | 0.0000 | 0.0217 | 0.9274 | 0.0706 | 0.0000 | 0.1931 | 0.9798 | 0.1970 | |
| CTB | 0.7742 | 0.5997 | 0.3600 | 0.2851 | 0.7792 | 0.2090 | 0.2055 | 0.2123 | 0.0028 | 0.9744 | 0.0378 | 0.7317 | 0.7026 | 0.8316 | 0.1212 | |
| CTT | 0.7922 | 0.4234 | 0.2714 | 0.4009 | 0.7685 | 0.0494 | 0.0986 | 0.1122 | 0.0127 | 0.9356 | 0.1407 | 0.3906 | 0.8731 | 0.0000 | 0.0758 | |
| DL1 | 0.5752 | 0.4552 | 0.1886 | 0.3686 | 0.8903 | 0.1281 | 0.3605 | 0.1261 | 0.2006 | 0.9737 | 0.0009 | 0.2995 | 0.9937 | 0.9864 | 0.0000 | |
| IPA | 0.6742 | 0.5416 | 0.3156 | 0.4530 | 0.7626 | 0.3416 | 0.7348 | 0.1202 | 0.0050 | 0.9442 | 0.1746 | 1.0000 | 0.4381 | 0.9610 | 0.9848 | |
| PBP | 0.6722 | 0.5324 | 0.2407 | 0.5458 | 0.6987 | 0.2022 | 0.0216 | 0.1414 | 0.0047 | 0.9028 | 0.4794 | 0.4632 | 0.8867 | 0.9347 | 0.1818 | |
| PMP | 0.6849 | 0.4556 | 0.2622 | 0.4801 | 0.7680 | 0.1034 | 0.1137 | 0.1141 | 0.0044 | 0.8686 | 0.1288 | 0.4592 | 0.8803 | 0.6599 | 0.0909 | |
| PMS | 0.7492 | 0.6148 | 0.3909 | 0.3147 | 0.7770 | 0.1258 | 0.2878 | 0.1651 | 0.0259 | 0.8307 | 0.1590 | 0.4363 | 0.8837 | 0.8778 | 0.0303 | |
| PRC | 0.5428 | 0.3737 | 0.1856 | 0.2925 | 0.6343 | 0.0764 | 0.2175 | 0.1077 | 0.0302 | 0.8681 | 0.1008 | 0.4284 | 0.8575 | 0.9005 | 0.4091 | |
| PSC | 0.6803 | 0.5145 | 0.2812 | 0.2712 | 0.7184 | 0.0135 | 0.1192 | 0.1280 | 0.0856 | 0.8162 | 0.4057 | 0.4899 | 0.9360 | 0.9480 | 0.9394 | |
| PTS | 0.6528 | 0.4453 | 0.2649 | 0.3640 | 0.7808 | 0.0045 | 0.0479 | 0.1113 | 0.0138 | 0.9243 | 0.5749 | 0.5288 | 0.8059 | 0.8831 | 0.8182 | |
| SDC | 0.5632 | 0.4019 | 0.1848 | 0.2563 | 0.7084 | 0.3258 | 0.4422 | 0.1196 | 0.0007 | 0.9398 | 0.0000 | 0.6380 | 0.4659 | 0.9151 | 0.1667 | |
| SDG | 0.7367 | 0.6383 | 0.7464 | 0.5981 | 0.7071 | 0.1281 | 0.1436 | 0.1507 | 0.0141 | 0.0000 | 0.3269 | 0.5176 | 0.9049 | 0.8960 | 0.0303 | |
| TPP | 0.5877 | 0.3900 | 0.1954 | 0.4179 | 0.9457 | 0.1056 | 0.0412 | 0.1076 | 0.0032 | 0.8008 | 0.1325 | 0.6188 | 0.8638 | 0.6904 | 0.3030 | |
| VBC | 0.8815 | 0.5873 | 0.4001 | 0.3606 | 0.7257 | 0.1371 | 0.2407 | 0.1290 | 0.0082 | 0.9117 | 0.0697 | 0.4334 | 0.9781 | 0.7286 | 0.0455 | |
| VCM | 0.7105 | 0.6367 | 0.3830 | 0.0000 | 1.0000 | 0.4629 | 0.1857 | 1.0000 | 0.0104 | 1.0000 | 0.0365 | 0.9441 | 0.0000 | 0.9706 | 0.2879 | |
| VSM | 0.8062 | 0.7195 | 0.3728 | 0.4685 | 0.7256 | 0.2022 | 0.4629 | 0.1999 | 0.1522 | 0.9252 | 0.1132 | 0.4467 | 0.9328 | 0.9113 | 0.1970 | |
| **Average** | **0.6944** | **0.5471** | **0.3446** | **0.4038** | **0.7061** | **0.2126** | **0.2882** | **0.1879** | **0.0683** | **0.8798** | **0.2118** | **0.5552** | **0.8177** | **0.8503** | **0.4152** | |

**Table S12. Positive distance from average for all indicators in 2020 based on EDAS**

| Company | Indicator | | | | | | | | | | | | | | |  |
| --- | --- | --- | --- | --- | --- | --- | --- | --- | --- | --- | --- | --- | --- | --- | --- | --- |
|  | I1 | I2 | I3 | I4 | I5 | I6 | I7 | I8 | I9 | I10 | I11 | I12 | I13 | I14 | I15 | |
| CAV | 0.3199 | 0.2306 | 1.0549 | 0.2411 | 0.0000 | 0.0000 | 0.0000 | 0.0000 | 0.0000 | 0.0000 | 0.0000 | 0.2032 | 0.0000 | 0.1179 | 0.5985 | |
| GEX | 0.0000 | 0.0000 | 0.0000 | 0.2460 | 0.0153 | 0.0000 | 0.0000 | 0.0000 | 0.0000 | 0.0000 | 0.0000 | 0.0365 | 0.0000 | 0.0208 | 0.0000 | |
| GMD | 0.0000 | 0.0000 | 0.0000 | 0.0000 | 0.0000 | 0.0000 | 0.0000 | 0.0000 | 0.0000 | 0.0000 | 0.0000 | 0.0000 | 0.3144 | 0.0000 | 0.0000 | |
| GSP | 0.0690 | 0.1288 | 0.0000 | 0.1616 | 0.0239 | 0.2156 | 0.0000 | 0.0000 | 0.6568 | 0.0543 | 0.7148 | 0.2963 | 0.0000 | 0.0000 | 0.0000 | |
| HAH | 0.0094 | 0.1514 | 0.1044 | 0.1076 | 0.0236 | 0.7125 | 0.9934 | 0.0000 | 0.0000 | 0.0000 | 0.0000 | 0.0000 | 0.0000 | 0.0000 | 0.0000 | |
| L10 | 0.0000 | 0.0000 | 0.0000 | 0.0000 | 0.0455 | 0.0000 | 0.2404 | 0.0000 | 0.0000 | 0.0000 | 0.0000 | 0.4108 | 0.0000 | 0.1837 | 0.0511 | |
| MSN | 0.0000 | 0.0000 | 0.0000 | 1.4762 | 0.0000 | 0.0000 | 0.0000 | 0.0000 | 0.0000 | 0.0000 | 3.7219 | 0.0000 | 0.0000 | 0.0587 | 0.0000 | |
| PAC | 0.2666 | 0.0815 | 0.1555 | 0.0000 | 0.0035 | 0.0000 | 0.0000 | 0.0000 | 0.0000 | 0.0567 | 2.9138 | 0.0183 | 0.0000 | 0.1619 | 0.8905 | |
| PDN | 0.3907 | 0.8278 | 1.2976 | 0.1013 | 0.0349 | 0.0000 | 0.2953 | 0.0257 | 13.6395 | 0.0000 | 0.0000 | 0.0000 | 0.0000 | 0.0000 | 0.0000 | |
| PJT | 0.0420 | 0.0110 | 0.0000 | 0.0000 | 0.0004 | 0.0000 | 0.0000 | 0.0000 | 0.0000 | 0.1352 | 2.7527 | 0.1180 | 0.0000 | 0.0000 | 0.0000 | |
| PVP | 0.1549 | 0.3121 | 0.0458 | 0.0091 | 0.1117 | 0.6173 | 1.0291 | 0.1060 | 1.3301 | 0.0054 | 0.0000 | 0.0285 | 0.0000 | 0.0000 | 0.0000 | |
| PVT | 0.0218 | 0.0794 | 0.0000 | 0.0000 | 0.0299 | 0.4588 | 0.1909 | 0.0000 | 0.0000 | 0.0000 | 0.0000 | 0.0190 | 0.0000 | 0.0000 | 0.0000 | |
| REE | 0.0895 | 0.2285 | 0.7118 | 0.2192 | 0.0000 | 0.2579 | 0.3901 | 0.0000 | 0.0000 | 0.0000 | 0.0000 | 0.0000 | 0.0000 | 0.0000 | 0.0000 | |
| SRF | 0.0000 | 0.0000 | 0.0000 | 0.0000 | 0.0000 | 0.0000 | 0.0000 | 0.0000 | 0.0000 | 0.0000 | 0.0000 | 0.2570 | 0.0000 | 0.2085 | 0.8175 | |
| STG | 0.0000 | 0.0000 | 0.0000 | 0.1526 | 0.0000 | 0.2791 | 0.7375 | 0.0976 | 0.0000 | 0.0000 | 0.0000 | 0.0000 | 0.0513 | 0.0000 | 0.0511 | |
| SVI | 0.4401 | 0.7063 | 1.9021 | 0.0000 | 0.0268 | 0.0148 | 0.0000 | 0.2883 | 0.0000 | 0.0000 | 0.0000 | 0.0000 | 0.0000 | 0.0000 | 0.8175 | |
| TCL | 0.0919 | 0.3572 | 0.1075 | 0.2087 | 0.0000 | 0.1311 | 0.0000 | 1.8944 | 1.3952 | 0.0000 | 0.0000 | 0.0000 | 0.0000 | 0.0000 | 0.2336 | |
| TDP | 0.0471 | 0.0000 | 0.0000 | 0.2150 | 0.0226 | 0.0000 | 0.0000 | 0.0000 | 0.0000 | 0.0407 | 0.0000 | 0.0000 | 0.0000 | 0.0836 | 0.6715 | |
| THI | 0.0323 | 0.0000 | 0.1750 | 0.0000 | 0.0160 | 0.0000 | 0.0252 | 0.0000 | 0.0000 | 0.0000 | 0.0000 | 0.0000 | 0.0000 | 0.0000 | 0.2701 | |
| TMS | 0.1046 | 0.2692 | 0.4686 | 0.6541 | 0.0151 | 0.0000 | 0.2327 | 0.0000 | 9.5593 | 0.0253 | 0.0379 | 0.2308 | 0.0000 | 0.0000 | 0.0000 | |
| TV2 | 0.3576 | 0.3114 | 1.0210 | 0.0117 | 0.0222 | 0.0994 | 0.2353 | 3.1679 | 0.0000 | 0.0000 | 0.0000 | 0.0994 | 0.0000 | 0.0439 | 0.2701 | |
| VIP | 0.0000 | 0.0000 | 0.0000 | 0.0000 | 0.2176 | 1.3784 | 0.4415 | 0.0000 | 0.0000 | 0.0285 | 0.0000 | 0.0000 | 0.2521 | 0.0000 | 0.0000 | |
| VOS | 0.0000 | 0.0000 | 0.0000 | 0.0000 | 0.0000 | 0.0000 | 0.0000 | 0.0000 | 0.0000 | 0.0000 | 0.0000 | 0.6296 | 0.0302 | 0.0974 | 0.0000 | |
| VTB | 0.0000 | 0.0859 | 0.0000 | 0.3798 | 0.0769 | 0.8816 | 0.0000 | 0.8535 | 0.0000 | 0.1461 | 0.0154 | 0.0000 | 0.0000 | 0.0000 | 0.5255 | |
| VTO | 0.0000 | 0.0000 | 0.0000 | 0.0000 | 0.0038 | 0.0254 | 0.4101 | 0.0000 | 0.0000 | 0.0089 | 1.8143 | 0.0000 | 0.0256 | 0.0000 | 0.0000 | |
| ARM | 0.0000 | 0.0000 | 0.0000 | 0.0000 | 0.0000 | 0.0000 | 0.1633 | 0.0000 | 0.0000 | 0.0000 | 0.0000 | 0.0407 | 0.1843 | 0.4975 | 1.0000 | |
| BBS | 0.0000 | 0.0000 | 0.0000 | 0.1175 | 0.0385 | 0.0000 | 0.0000 | 0.0000 | 0.0000 | 0.0000 | 0.0000 | 0.1496 | 0.0000 | 0.2033 | 0.8175 | |
| CDN | 0.1187 | 0.5390 | 0.0000 | 0.1408 | 0.0250 | 3.7040 | 0.6751 | 0.5029 | 0.0000 | 0.0851 | 0.0000 | 0.0000 | 0.0822 | 0.0000 | 0.0000 | |
| CIA | 0.0000 | 0.0000 | 0.0000 | 0.0000 | 0.0000 | 1.3256 | 2.4693 | 0.0000 | 0.0000 | 0.0000 | 0.0000 | 1.0000 | 0.7638 | 0.0000 | 0.5255 | |
| CTB | 0.1150 | 0.0961 | 0.0447 | 0.0000 | 0.1036 | 0.0000 | 0.0000 | 0.1299 | 0.0000 | 0.0000 | 0.0000 | 0.0000 | 0.1407 | 0.0220 | 0.7080 | |
| CTT | 0.1408 | 0.0000 | 0.0000 | 0.0000 | 0.0884 | 0.0000 | 0.0000 | 0.0000 | 0.0000 | 0.0000 | 0.0000 | 0.2965 | 0.0000 | 1.0000 | 0.8175 | |
| DL1 | 0.0000 | 0.0000 | 0.0000 | 0.0000 | 0.2609 | 0.0000 | 0.2508 | 0.0000 | 1.9365 | 0.0000 | 0.0000 | 0.4606 | 0.0000 | 0.0000 | 1.0000 | |
| IPA | 0.0000 | 0.0000 | 0.0000 | 0.1216 | 0.0800 | 0.6068 | 1.5493 | 0.0000 | 0.0000 | 0.0000 | 0.0000 | 0.0000 | 0.4643 | 0.0000 | 0.0000 | |
| PBP | 0.0000 | 0.0000 | 0.0000 | 0.3516 | 0.0000 | 0.0000 | 0.0000 | 0.0000 | 0.0000 | 0.0000 | 1.2635 | 0.1658 | 0.0000 | 0.0000 | 0.5620 | |
| PMP | 0.0000 | 0.0000 | 0.0000 | 0.1888 | 0.0877 | 0.0000 | 0.0000 | 0.0000 | 0.0000 | 0.0127 | 0.0000 | 0.1729 | 0.0000 | 0.2239 | 0.7810 | |
| PMS | 0.0790 | 0.1237 | 0.1343 | 0.0000 | 0.1004 | 0.0000 | 0.0000 | 0.0000 | 0.0000 | 0.0558 | 0.0000 | 0.2141 | 0.0000 | 0.0000 | 0.9270 | |
| PRC | 0.0000 | 0.0000 | 0.0000 | 0.0000 | 0.0000 | 0.0000 | 0.0000 | 0.0000 | 0.0000 | 0.0133 | 0.0000 | 0.2284 | 0.0000 | 0.0000 | 0.0146 | |
| PSC | 0.0000 | 0.0000 | 0.0000 | 0.0000 | 0.0174 | 0.0000 | 0.0000 | 0.0000 | 0.2536 | 0.0724 | 0.9159 | 0.1176 | 0.0000 | 0.0000 | 0.0000 | |
| PTS | 0.0000 | 0.0000 | 0.0000 | 0.0000 | 0.1058 | 0.0000 | 0.0000 | 0.0000 | 0.0000 | 0.0000 | 1.7148 | 0.0475 | 0.0144 | 0.0000 | 0.0000 | |
| SDC | 0.0000 | 0.0000 | 0.0000 | 0.0000 | 0.0033 | 0.5328 | 0.5340 | 0.0000 | 0.0000 | 0.0000 | 0.0000 | 0.0000 | 0.4303 | 0.0000 | 0.5985 | |
| SDG | 0.0610 | 0.1666 | 1.1661 | 0.4809 | 0.0014 | 0.0000 | 0.0000 | 0.0000 | 0.0000 | 1.0000 | 0.5436 | 0.0677 | 0.0000 | 0.0000 | 0.9270 | |
| TPP | 0.0000 | 0.0000 | 0.0000 | 0.0348 | 0.3394 | 0.0000 | 0.0000 | 0.0000 | 0.0000 | 0.0898 | 0.0000 | 0.0000 | 0.0000 | 0.1881 | 0.2701 | |
| VBC | 0.2695 | 0.0735 | 0.1612 | 0.0000 | 0.0278 | 0.0000 | 0.0000 | 0.0000 | 0.0000 | 0.0000 | 0.0000 | 0.2195 | 0.0000 | 0.1431 | 0.8905 | |
| VCM | 0.0233 | 0.1637 | 0.1116 | 0.0000 | 0.4163 | 1.1776 | 0.0000 | 4.3230 | 0.0000 | 0.0000 | 0.0000 | 0.0000 | 1.0000 | 0.0000 | 0.3066 | |
| VSM | 0.1610 | 0.3150 | 0.0819 | 0.1602 | 0.0277 | 0.0000 | 0.6058 | 0.0640 | 1.2282 | 0.0000 | 0.0000 | 0.1955 | 0.0000 | 0.0000 | 0.5255 | |

**Table S13. Negative distance from average for all indicators in 2020 based on EDAS**

| Company | Indicator | | | | | | | | | | | | | | |  |
| --- | --- | --- | --- | --- | --- | --- | --- | --- | --- | --- | --- | --- | --- | --- | --- | --- |
|  | I1 | I2 | I3 | I4 | I5 | I6 | I7 | I8 | I9 | I10 | I11 | I12 | I13 | I14 | I15 | |
| CAV | 0.0000 | 0.0000 | 0.0000 | 0.0000 | 0.0380 | 0.3129 | 0.3202 | 0.2474 | 0.9079 | 0.0180 | 0.2082 | 0.0000 | 0.2230 | 0.0000 | 0.0000 | |
| GEX | 0.0196 | 0.1207 | 0.1480 | 0.0000 | 0.0000 | 0.3023 | 0.0745 | 0.3735 | 0.9319 | 0.0509 | 0.4759 | 0.0000 | 0.1194 | 0.0000 | 0.5328 | |
| GMD | 0.1132 | 0.0908 | 0.2607 | 0.0192 | 0.0298 | 0.7569 | 0.3070 | 0.2936 | 0.6483 | 0.1005 | 0.3200 | 0.7121 | 0.0000 | 0.1491 | 0.6423 | |
| GSP | 0.0000 | 0.0000 | 0.1596 | 0.0000 | 0.0000 | 0.0000 | 0.2599 | 0.1046 | 0.0000 | 0.0000 | 0.0000 | 0.0000 | 0.1716 | 0.1109 | 0.5693 | |
| HAH | 0.0000 | 0.0000 | 0.0000 | 0.0000 | 0.0000 | 0.0000 | 0.0000 | 0.1383 | 0.6773 | 0.0015 | 0.1518 | 0.1506 | 0.0227 | 0.1531 | 1.0803 | |
| L10 | 0.0724 | 0.2436 | 0.1808 | 0.1454 | 0.0000 | 0.0486 | 0.0000 | 0.3253 | 0.9409 | 0.0493 | 0.7282 | 0.0000 | 0.2170 | 0.0000 | 0.0000 | |
| MSN | 0.1724 | 0.2770 | 0.2862 | 0.0000 | 0.1404 | 0.7674 | 0.7484 | 0.4127 | 0.9292 | 0.0148 | 0.0000 | 0.2563 | 0.1027 | 0.0000 | 1.0438 | |
| PAC | 0.0000 | 0.0000 | 0.0000 | 0.0362 | 0.0000 | 0.4397 | 1.0000 | 0.3093 | 0.9620 | 0.0000 | 0.0000 | 0.0000 | 0.2053 | 0.0000 | 0.0000 | |
| PDN | 0.0000 | 0.0000 | 0.0000 | 0.0000 | 0.0000 | 0.1121 | 0.0000 | 0.0000 | 0.0000 | 0.0686 | 0.2164 | 0.5339 | 0.0233 | 0.1307 | 0.1679 | |
| PJT | 0.0000 | 0.0000 | 0.1822 | 0.1515 | 0.0000 | 1.0000 | 0.8986 | 0.3714 | 0.7408 | 0.0000 | 0.0000 | 0.0000 | 0.0867 | 0.0914 | 0.7518 | |
| PVP | 0.0000 | 0.0000 | 0.0000 | 0.0000 | 0.0000 | 0.0000 | 0.0000 | 0.0000 | 0.0000 | 0.0000 | 0.3980 | 0.0000 | 0.2122 | 0.1470 | 1.0438 | |
| PVT | 0.0000 | 0.0000 | 0.0620 | 0.0683 | 0.0000 | 0.0000 | 0.0000 | 0.1377 | 0.0832 | 0.0548 | 0.1518 | 0.0000 | 0.1274 | 0.1297 | 0.7883 | |
| REE | 0.0000 | 0.0000 | 0.0000 | 0.0000 | 0.0055 | 0.0000 | 0.0000 | 0.2472 | 0.9443 | 0.0562 | 0.5610 | 0.4385 | 0.0530 | 0.1464 | 1.1168 | |
| SRF | 0.0641 | 0.2116 | 0.2702 | 0.1832 | 0.0384 | 0.4397 | 0.2972 | 0.3948 | 0.9434 | 0.0950 | 0.9005 | 0.0000 | 0.0935 | 0.0000 | 0.0000 | |
| STG | 0.0952 | 0.0254 | 0.2779 | 0.0000 | 0.0052 | 0.0000 | 0.0000 | 0.0000 | 0.3127 | 0.0248 | 0.5169 | 0.1931 | 0.0000 | 0.1419 | 0.0000 | |
| SVI | 0.0000 | 0.0000 | 0.0000 | 0.0123 | 0.0000 | 0.0000 | 0.0278 | 0.0000 | 0.8415 | 0.0689 | 0.6041 | 0.0561 | 0.2010 | 0.0526 | 0.0000 | |
| TCL | 0.0000 | 0.0000 | 0.0000 | 0.0000 | 0.0031 | 0.0000 | 0.0584 | 0.0000 | 0.0000 | 0.0741 | 0.3651 | 0.0188 | 0.0270 | 0.1302 | 0.0000 | |
| TDP | 0.0000 | 0.0545 | 0.2144 | 0.0000 | 0.0000 | 0.3235 | 0.8347 | 0.3892 | 1.0000 | 0.0000 | 0.1877 | 0.0537 | 0.1359 | 0.0000 | 0.0000 | |
| THI | 0.0000 | 0.0239 | 0.0000 | 0.0821 | 0.0000 | 0.1226 | 0.0000 | 0.3610 | 0.9593 | 0.0571 | 0.2718 | 0.0468 | 0.0957 | 0.0069 | 0.0000 | |
| TMS | 0.0000 | 0.0000 | 0.0000 | 0.0000 | 0.0000 | 0.2178 | 0.0000 | 0.1654 | 0.0000 | 0.0000 | 0.0000 | 0.0000 | 0.1511 | 0.1215 | 0.2409 | |
| TV2 | 0.0000 | 0.0000 | 0.0000 | 0.0000 | 0.0000 | 0.0000 | 0.0000 | 0.0000 | 0.8830 | 0.1114 | 0.5415 | 0.0000 | 0.1103 | 0.0000 | 0.0000 | |
| VIP | 0.1081 | 0.0545 | 0.3662 | 0.2395 | 0.0000 | 0.0000 | 0.0000 | 0.2355 | 0.9297 | 0.0000 | 0.3282 | 0.0010 | 0.0000 | 0.1677 | 1.4088 | |
| VOS | 1.0000 | 0.8218 | 0.8536 | 0.2429 | 0.9478 | 0.6195 | 0.3197 | 0.5017 | 0.7399 | 0.0955 | 0.3651 | 0.0000 | 0.0000 | 0.0000 | 1.1533 | |
| VTB | 0.0210 | 0.0000 | 0.1782 | 0.0000 | 0.0000 | 0.0000 | 0.0320 | 0.0000 | 0.9773 | 0.0000 | 0.0000 | 0.4778 | 0.1066 | 0.1292 | 0.0000 | |
| VTO | 0.0999 | 0.0908 | 0.3820 | 0.3299 | 0.0000 | 0.0000 | 0.0000 | 0.3204 | 0.8623 | 0.0000 | 0.0000 | 0.0270 | 0.0000 | 0.1400 | 0.7153 | |
| ARM | 0.1424 | 0.2901 | 0.4165 | 0.5461 | 0.0928 | 0.3446 | 0.0000 | 0.4026 | 0.8720 | 0.0331 | 0.5323 | 0.0000 | 0.0000 | 0.0000 | 0.0000 | |
| BBS | 0.0957 | 0.2407 | 0.3694 | 0.0000 | 0.0000 | 0.5983 | 0.2682 | 0.4137 | 0.8689 | 0.0815 | 0.8256 | 0.0000 | 0.0497 | 0.0000 | 0.0000 | |
| CDN | 0.0000 | 0.0000 | 0.0735 | 0.0000 | 0.0000 | 0.0000 | 0.0000 | 0.0000 | 0.3532 | 0.0000 | 0.2595 | 0.6748 | 0.0000 | 0.1761 | 1.0073 | |
| CIA | 0.5143 | 1.0000 | 1.0000 | 0.9392 | 1.0000 | 0.0000 | 0.0000 | 1.0000 | 0.6827 | 0.0541 | 0.6667 | 0.0000 | 0.0000 | 0.1523 | 0.0000 | |
| CTB | 0.0000 | 0.0000 | 0.0000 | 0.2940 | 0.0000 | 0.0169 | 0.2871 | 0.0000 | 0.9591 | 0.1075 | 0.8215 | 0.3180 | 0.0000 | 0.0000 | 0.0000 | |
| CTT | 0.0000 | 0.2261 | 0.2124 | 0.0072 | 0.0000 | 0.7674 | 0.6578 | 0.4026 | 0.8134 | 0.0634 | 0.3354 | 0.0000 | 0.0677 | 0.0000 | 0.0000 | |
| DL1 | 0.1716 | 0.1679 | 0.4526 | 0.0874 | 0.0000 | 0.3975 | 0.0000 | 0.3288 | 0.0000 | 0.1067 | 0.9959 | 0.0000 | 0.2153 | 0.1601 | 0.0000 | |
| IPA | 0.0290 | 0.0101 | 0.0841 | 0.0000 | 0.0000 | 0.0000 | 0.0000 | 0.3604 | 0.9270 | 0.0732 | 0.1754 | 0.8011 | 0.0000 | 0.1302 | 1.3723 | |
| PBP | 0.0320 | 0.0268 | 0.3015 | 0.0000 | 0.0104 | 0.0486 | 0.9252 | 0.2475 | 0.9306 | 0.0261 | 0.0000 | 0.0000 | 0.0844 | 0.0992 | 0.0000 | |
| PMP | 0.0137 | 0.1672 | 0.2390 | 0.0000 | 0.0000 | 0.5137 | 0.6054 | 0.3928 | 0.9355 | 0.0000 | 0.3918 | 0.0000 | 0.0766 | 0.0000 | 0.0000 | |
| PMS | 0.0000 | 0.0000 | 0.0000 | 0.2207 | 0.0000 | 0.4080 | 0.0015 | 0.1214 | 0.6207 | 0.0000 | 0.2492 | 0.0000 | 0.0807 | 0.0323 | 0.0000 | |
| PRC | 0.2184 | 0.3170 | 0.4613 | 0.2756 | 0.1017 | 0.6406 | 0.2456 | 0.4267 | 0.5575 | 0.0000 | 0.5241 | 0.0000 | 0.0488 | 0.0591 | 0.0000 | |
| PSC | 0.0203 | 0.0596 | 0.1839 | 0.3284 | 0.0000 | 0.9366 | 0.5866 | 0.3189 | 0.0000 | 0.0000 | 0.0000 | 0.0000 | 0.1447 | 0.1149 | 1.2628 | |
| PTS | 0.0599 | 0.1861 | 0.2312 | 0.0986 | 0.0000 | 0.9789 | 0.8340 | 0.4074 | 0.7985 | 0.0506 | 0.0000 | 0.0000 | 0.0000 | 0.0386 | 0.9708 | |
| SDC | 0.1889 | 0.2654 | 0.4637 | 0.3653 | 0.0000 | 0.0000 | 0.0000 | 0.3633 | 0.9897 | 0.0682 | 1.0000 | 0.1492 | 0.0000 | 0.0763 | 0.0000 | |
| SDG | 0.0000 | 0.0000 | 0.0000 | 0.0000 | 0.0000 | 0.3975 | 0.5018 | 0.1976 | 0.7938 | 0.0000 | 0.0000 | 0.0000 | 0.1067 | 0.0538 | 0.0000 | |
| TPP | 0.1536 | 0.2872 | 0.4331 | 0.0000 | 0.0000 | 0.5032 | 0.8571 | 0.4272 | 0.9535 | 0.0000 | 0.3744 | 0.1145 | 0.0564 | 0.0000 | 0.0000 | |
| VBC | 0.0000 | 0.0000 | 0.0000 | 0.1072 | 0.0000 | 0.3552 | 0.1648 | 0.3133 | 0.8807 | 0.0362 | 0.6708 | 0.0000 | 0.1963 | 0.0000 | 0.0000 | |
| VCM | 0.0000 | 0.0000 | 0.0000 | 1.0000 | 0.0000 | 0.0000 | 0.3557 | 0.0000 | 0.8477 | 0.1366 | 0.8277 | 0.7005 | 0.0000 | 0.1415 | 0.0000 | |
| VSM | 0.0000 | 0.0000 | 0.0000 | 0.0000 | 0.0000 | 0.0486 | 0.0000 | 0.0000 | 0.0000 | 0.0516 | 0.4657 | 0.0000 | 0.1409 | 0.0717 | 0.0000 | |

**Table S14. The normalized sum of the weighted positive and negative distance from the average, the appraisal score, and rank in 2020 based on EDAS.**

| Company | NSP | NSN | AS | Rank |
| --- | --- | --- | --- | --- |
| CAV | 0.1908 | 0.7006 | 0.4457 | 14 |
| GEX | 0.0175 | 0.5392 | 0.2783 | 37 |
| GMD | 0.0248 | 0.3823 | 0.2035 | 44 |
| GSP | 0.1570 | 0.7601 | 0.4585 | 13 |
| HAH | 0.1413 | 0.5896 | 0.3655 | 25 |
| L10 | 0.0628 | 0.6142 | 0.3385 | 30 |
| MSN | 0.4119 | 0.2598 | 0.3358 | 31 |
| PAC | 0.4026 | 0.6099 | 0.5062 | 8 |
| PDN | 1.0000 | 0.8213 | 0.9106 | 1 |
| PJT | 0.2612 | 0.3796 | 0.3204 | 33 |
| PVP | 0.2386 | 0.6421 | 0.4403 | 17 |
| PVT | 0.0503 | 0.7115 | 0.3809 | 22 |
| REE | 0.1146 | 0.4213 | 0.2680 | 40 |
| SRF | 0.1241 | 0.4835 | 0.3038 | 35 |
| STG | 0.0984 | 0.7879 | 0.4431 | 15 |
| SVI | 0.2820 | 0.7383 | 0.5101 | 7 |
| TCL | 0.2736 | 0.8978 | 0.5857 | 3 |
| TDP | 0.1015 | 0.5810 | 0.3412 | 28 |
| THI | 0.0465 | 0.7433 | 0.3949 | 19 |
| TMS | 0.7028 | 0.8621 | 0.7824 | 2 |
| TV2 | 0.3492 | 0.7713 | 0.5602 | 4 |
| VIP | 0.1518 | 0.3797 | 0.2658 | 41 |
| VOS | 0.0461 | 0.0000 | 0.0231 | 45 |
| VTB | 0.2058 | 0.7686 | 0.4872 | 9 |
| VTO | 0.1966 | 0.5799 | 0.3883 | 21 |
| ARM | 0.1769 | 0.5575 | 0.3672 | 24 |
| BBS | 0.1257 | 0.5032 | 0.3145 | 34 |
| CDN | 0.3601 | 0.5737 | 0.4669 | 11 |
| CIA | 0.4550 | 0.2043 | 0.3296 | 32 |
| CTB | 0.1218 | 0.6177 | 0.3698 | 23 |
| CTT | 0.1850 | 0.5434 | 0.3642 | 26 |
| DL1 | 0.2977 | 0.5859 | 0.4418 | 16 |
| IPA | 0.2047 | 0.3706 | 0.2877 | 36 |
| PBP | 0.2068 | 0.6455 | 0.4261 | 18 |
| PMP | 0.1313 | 0.5662 | 0.3488 | 27 |
| PMS | 0.1483 | 0.7806 | 0.4645 | 12 |
| PRC | 0.0163 | 0.5235 | 0.2699 | 38 |
| PSC | 0.1089 | 0.3740 | 0.2414 | 43 |
| PTS | 0.1614 | 0.3215 | 0.2415 | 42 |
| SDC | 0.1785 | 0.4993 | 0.3389 | 29 |
| SDG | 0.3264 | 0.7362 | 0.5313 | 6 |
| TPP | 0.0687 | 0.4696 | 0.2692 | 39 |
| VBC | 0.1539 | 0.6296 | 0.3918 | 20 |
| VCM | 0.4852 | 0.4830 | 0.4841 | 10 |
| VSM | 0.2370 | 0.8779 | 0.5575 | 5 |

**Table S15. The criteria's average solution in 2021 based on EDAS.**

| Company | Indicator | | | | | | | | | | | | | | |  |
| --- | --- | --- | --- | --- | --- | --- | --- | --- | --- | --- | --- | --- | --- | --- | --- | --- |
|  | I1 | I2 | I3 | I4 | I5 | I6 | I7 | I8 | I9 | I10 | I11 | I12 | I13 | I14 | I15 | |
| CAV | 0.4171 | 0.4555 | 0.7150 | 0.3984 | 0.3107 | 0.1253 | 0.0061 | 0.1728 | 0.0014 | 0.8467 | 0.1077 | 0.6219 | 0.9887 | 0.6632 | 0.0909 | |
| GEX | 0.2688 | 0.3677 | 0.2990 | 0.8095 | 0.3309 | 0.1597 | 0.1440 | 0.1528 | 0.0013 | 0.8144 | 0.0943 | 0.6724 | 0.9190 | 0.8565 | 0.5584 | |
| GMD | 0.2909 | 0.4687 | 0.3636 | 0.5624 | 0.3560 | 0.0246 | 0.1264 | 0.1971 | 0.0270 | 0.8698 | 0.1096 | 0.8425 | 0.8149 | 0.9706 | 0.4935 | |
| GSP | 0.3041 | 0.4463 | 0.2760 | 0.4415 | 0.3131 | 0.2457 | 0.2129 | 0.2047 | 0.0860 | 0.6489 | 0.1692 | 0.5985 | 0.9690 | 0.9450 | 0.6234 | |
| HAH | 0.5272 | 0.7711 | 1.0000 | 0.8442 | 0.4487 | 0.3489 | 0.4214 | 0.3213 | 0.0250 | 0.5391 | 0.1393 | 0.8502 | 0.9226 | 0.9699 | 0.6753 | |
| L10 | 0.2512 | 0.3374 | 0.2964 | 0.3303 | 0.3153 | 0.1916 | 0.2085 | 0.1571 | 0.0021 | 0.8692 | 0.0369 | 0.5703 | 1.0000 | 0.5891 | 0.3247 | |
| MSN | 0.5004 | 0.5004 | 0.8575 | 0.4992 | 0.8419 | 0.1499 | 0.1693 | 0.1585 | 0.0033 | 0.7531 | 0.5593 | 0.7534 | 0.9173 | 0.9000 | 0.7532 | |
| PAC | 0.4538 | 0.5015 | 0.4981 | 0.4202 | 0.3380 | 0.1278 | 0.0000 | 0.1799 | 0.0019 | 0.4411 | 0.6644 | 0.6669 | 0.9814 | 0.7622 | 0.0390 | |
| PDN | 0.4748 | 0.7198 | 0.9061 | 0.4897 | 0.3142 | 0.1720 | 0.2088 | 0.2801 | 1.0000 | 0.8264 | 0.1166 | 0.7959 | 0.8995 | 0.9612 | 0.4156 | |
| PJT | 0.2834 | 0.4219 | 0.2628 | 0.4277 | 0.3042 | 0.0418 | 0.0534 | 0.1495 | 0.0129 | 0.2047 | 0.4558 | 0.6293 | 0.9291 | 0.9685 | 0.7143 | |
| PVP | 0.3411 | 0.5327 | 0.3760 | 0.2475 | 0.3322 | 0.4668 | 0.5541 | 0.2752 | 0.0662 | 0.8675 | 0.0358 | 0.7183 | 0.9774 | 0.9820 | 0.6494 | |
| PVT | 0.3034 | 0.4589 | 0.3681 | 0.4112 | 0.3236 | 0.3268 | 0.2449 | 0.2018 | 0.0482 | 0.7979 | 0.0940 | 0.6832 | 0.9366 | 0.9662 | 0.7143 | |
| REE | 0.3420 | 0.5007 | 0.7400 | 0.4249 | 0.3444 | 0.2531 | 0.2650 | 0.1689 | 0.0024 | 0.8759 | 0.0396 | 0.8773 | 0.8775 | 0.9810 | 0.9481 | |
| SRF | 0.2442 | 0.3453 | 0.2316 | 0.1471 | 0.3511 | 0.1179 | 0.1364 | 0.1458 | 0.0008 | 0.9635 | 0.0066 | 0.6221 | 0.9147 | 0.7221 | 0.0909 | |
| STG | 0.3446 | 0.5619 | 0.4012 | 0.6929 | 0.3700 | 0.2703 | 0.3108 | 0.3140 | 0.0264 | 0.6367 | 0.0927 | 0.6857 | 0.9159 | 0.9732 | 0.3636 | |
| SVI | 0.3720 | 0.5316 | 0.8505 | 0.4656 | 0.2834 | 0.2457 | 0.2039 | 0.3086 | 0.0072 | 0.8759 | 0.0536 | 0.6502 | 0.9703 | 0.9009 | 0.0260 | |
| TCL | 0.4143 | 0.6485 | 0.4749 | 0.4645 | 0.3278 | 0.2260 | 0.2249 | 0.9582 | 0.1577 | 0.8616 | 0.0857 | 0.6880 | 0.9035 | 0.9556 | 0.2468 | |
| TDP | 0.3403 | 0.4084 | 0.3201 | 0.9354 | 0.2926 | 0.0983 | 0.0583 | 0.1456 | 0.0004 | 0.5637 | 0.1823 | 0.6504 | 0.9622 | 0.6795 | 0.1948 | |
| THI | 0.2508 | 0.3717 | 0.3391 | 0.3012 | 0.2936 | 0.1990 | 0.1277 | 0.1483 | 0.0006 | 0.8867 | 0.1001 | 0.6558 | 0.9172 | 0.8809 | 0.2987 | |
| TMS | 0.4256 | 0.6334 | 0.6851 | 1.0000 | 0.3274 | 0.2039 | 0.3075 | 0.2500 | 0.7697 | 0.5288 | 0.1566 | 0.6159 | 0.9636 | 0.9492 | 0.3377 | |
| TV2 | 0.4488 | 0.4973 | 0.6891 | 0.4621 | 0.3199 | 0.1327 | 0.2130 | 0.6676 | 0.0126 | 0.9957 | 0.0191 | 0.6205 | 0.9365 | 0.7012 | 0.0130 | |
| VIP | 0.1887 | 0.3209 | 0.2010 | 0.6228 | 0.2396 | 0.8428 | 0.2159 | 0.1444 | 0.0052 | 0.5452 | 0.1108 | 0.6016 | 0.8580 | 0.9924 | 0.6753 | |
| VOS | 1.0000 | 0.7980 | 0.5106 | 0.4805 | 0.0000 | 0.2088 | 0.1681 | 0.1810 | 0.0100 | 0.9571 | 0.1028 | 0.8108 | 0.8550 | 0.9136 | 0.7403 | |
| VTB | 0.2058 | 0.3475 | 0.2191 | 0.2038 | 0.2663 | 0.4816 | 0.1910 | 1.0000 | 0.0010 | 0.0000 | 0.1140 | 0.7503 | 0.9104 | 0.9747 | 0.2338 | |
| VTO | 0.3231 | 0.5066 | 0.3170 | 0.2747 | 0.4475 | 0.3514 | 0.4281 | 0.1981 | 0.0069 | 0.5921 | 0.2895 | 0.6592 | 0.8596 | 0.9715 | 0.5195 | |
| ARM | 0.2442 | 0.3363 | 0.2385 | 0.4507 | 0.3283 | 0.1671 | 0.2510 | 0.1469 | 0.0084 | 0.7564 | 0.0561 | 0.6470 | 0.8341 | 0.6955 | 0.0130 | |
| BBS | 0.2963 | 0.3753 | 0.3116 | 0.4500 | 0.3647 | 0.0860 | 0.1612 | 0.1456 | 0.0075 | 0.8429 | 0.0251 | 0.6127 | 0.9737 | 0.6534 | 0.0000 | |
| CDN | 0.3907 | 0.6898 | 0.3957 | 0.5365 | 0.3214 | 1.0000 | 0.3262 | 0.4042 | 0.0321 | 0.4667 | 0.1223 | 0.8454 | 0.8304 | 1.0000 | 0.5584 | |
| CIA | 0.0000 | 0.0000 | 0.0000 | 0.0000 | 0.4792 | 0.4717 | 0.5896 | 0.0000 | 0.0068 | 0.9257 | 0.0171 | 0.0000 | 0.0000 | 0.9826 | 0.2078 | |
| CTB | 0.3186 | 0.4137 | 0.3573 | 0.6241 | 0.2831 | 0.3366 | 0.1120 | 0.1591 | 0.0005 | 0.8784 | 0.0445 | 0.6582 | 0.8698 | 0.8286 | 0.4805 | |
| CTT | 0.3808 | 0.3537 | 0.3053 | 0.6697 | 0.3005 | 0.0393 | 0.0977 | 0.1443 | 0.0143 | 0.7722 | 0.1155 | 0.5877 | 0.9400 | 0.0000 | 0.0519 | |
| DL1 | 0.2275 | 0.3778 | 0.2282 | 0.0838 | 0.4596 | 0.1966 | 0.3111 | 0.1694 | 0.0049 | 1.0000 | 0.0429 | 0.5940 | 0.9804 | 0.9546 | 0.5325 | |
| IPA | 0.8780 | 1.0000 | 0.9951 | 0.4560 | 1.0000 | 0.8305 | 1.0000 | 0.2058 | 0.0131 | 0.7803 | 0.0866 | 1.0000 | 0.6856 | 0.9770 | 1.0000 | |
| PBP | 0.2941 | 0.4600 | 0.2818 | 0.5275 | 0.3129 | 0.2408 | 0.0978 | 0.2492 | 0.0056 | 0.5688 | 1.0000 | 0.6196 | 0.9222 | 0.9347 | 0.0779 | |
| PMP | 0.3076 | 0.3776 | 0.3125 | 0.7298 | 0.2989 | 0.0934 | 0.0665 | 0.1477 | 0.0040 | 0.4875 | 0.1342 | 0.6240 | 0.9239 | 0.6245 | 0.0390 | |
| PMS | 0.3683 | 0.5125 | 0.4711 | 0.4052 | 0.3378 | 0.1278 | 0.1218 | 0.2349 | 0.0135 | 0.6210 | 0.1099 | 0.6207 | 0.9293 | 0.8767 | 0.0000 | |
| PRC | 0.2363 | 0.3644 | 0.2895 | 0.3961 | 0.5737 | 0.0835 | 0.1530 | 0.1456 | 0.0212 | 0.5511 | 0.0745 | 0.6222 | 0.9060 | 0.9189 | 0.4545 | |
| PSC | 0.2582 | 0.3826 | 0.2726 | 0.4289 | 0.2893 | 0.0000 | 0.0910 | 0.1555 | 0.0830 | 0.5430 | 0.3063 | 0.6263 | 0.9572 | 0.9496 | 0.7532 | |
| PTS | 0.3296 | 0.4174 | 0.3600 | 0.4645 | 0.3697 | 0.0147 | 0.0725 | 0.1508 | 0.0144 | 0.7536 | 0.3625 | 0.6625 | 0.8703 | 0.9042 | 0.6883 | |
| SDC | 0.2335 | 0.3745 | 0.2498 | 0.3411 | 0.4160 | 0.3956 | 0.3492 | 0.2011 | 0.0000 | 0.8309 | 0.0000 | 0.7276 | 0.5572 | 0.9365 | 0.1558 | |
| SDG | 0.2308 | 0.3647 | 0.3504 | 0.2975 | 0.2655 | 0.1376 | 0.0909 | 0.1519 | 0.0070 | 0.1039 | 0.0984 | 0.6292 | 0.9372 | 0.8927 | 0.0649 | |
| TPP | 0.2146 | 0.3237 | 0.2122 | 0.7326 | 0.2899 | 0.1081 | 0.1083 | 0.1382 | 0.0026 | 0.5526 | 0.0880 | 0.6968 | 0.9525 | 0.7019 | 0.2597 | |
| VBC | 0.4363 | 0.4858 | 0.4641 | 0.4879 | 0.3151 | 0.1425 | 0.1675 | 0.1655 | 0.0073 | 0.7796 | 0.0564 | 0.6147 | 0.9806 | 0.7306 | 0.0130 | |
| VCM | 0.1937 | 0.3299 | 0.2167 | 0.4499 | 0.2383 | 0.5184 | 0.1406 | 0.7699 | 0.0532 | 0.8609 | 0.0472 | 0.7150 | 0.5915 | 0.9780 | 0.0779 | |
| VSM | 0.4612 | 0.6443 | 0.4271 | 0.6933 | 0.3327 | 0.2138 | 0.3335 | 0.5492 | 0.1598 | 0.7878 | 0.0818 | 0.6351 | 0.9463 | 0.9078 | 0.0519 | |
| **Average** | **0.3470** | **0.4675** | **0.4208** | **0.4796** | **0.3549** | **0.2492** | **0.2187** | **0.2559** | **0.0608** | **0.7028** | **0.1512** | **0.6673** | **0.8820** | **0.8573** | **0.3605** | |

**Table S16. Positive distance from average for all indicators in 2021 based on EDAS**

| Company | Indicator | | | | | | | | | | | | | | |  |
| --- | --- | --- | --- | --- | --- | --- | --- | --- | --- | --- | --- | --- | --- | --- | --- | --- |
|  | I1 | I2 | I3 | I4 | I5 | I6 | I7 | I8 | I9 | I10 | I11 | I12 | I13 | I14 | I15 | |
| CAV | 0.2019 | 0.0000 | 0.6991 | 0.0000 | 0.0000 | 0.0000 | 0.0000 | 0.0000 | 0.0000 | 0.0000 | 0.0000 | 0.0681 | 0.0000 | 0.2264 | 0.7478 | |
| GEX | 0.0000 | 0.0000 | 0.0000 | 0.6879 | 0.0000 | 0.0000 | 0.0000 | 0.0000 | 0.0000 | 0.0000 | 0.0000 | 0.0000 | 0.0000 | 0.0010 | 0.0000 | |
| GMD | 0.0000 | 0.0026 | 0.0000 | 0.1726 | 0.0032 | 0.0000 | 0.0000 | 0.0000 | 0.0000 | 0.0000 | 0.0000 | 0.0000 | 0.0761 | 0.0000 | 0.0000 | |
| GSP | 0.0000 | 0.0000 | 0.0000 | 0.0000 | 0.0000 | 0.0000 | 0.0000 | 0.0000 | 0.4142 | 0.0766 | 0.1188 | 0.1031 | 0.0000 | 0.0000 | 0.0000 | |
| HAH | 0.5192 | 0.6494 | 1.3763 | 0.7602 | 0.2644 | 0.3998 | 0.9267 | 0.2557 | 0.0000 | 0.2329 | 0.0000 | 0.0000 | 0.0000 | 0.0000 | 0.0000 | |
| L10 | 0.0000 | 0.0000 | 0.0000 | 0.0000 | 0.0000 | 0.0000 | 0.0000 | 0.0000 | 0.0000 | 0.0000 | 0.0000 | 0.1453 | 0.0000 | 0.3129 | 0.0993 | |
| MSN | 0.4420 | 0.0704 | 1.0375 | 0.0408 | 1.3725 | 0.0000 | 0.0000 | 0.0000 | 0.0000 | 0.0000 | 2.6981 | 0.0000 | 0.0000 | 0.0000 | 0.0000 | |
| PAC | 0.3076 | 0.0728 | 0.1835 | 0.0000 | 0.0000 | 0.0000 | 0.0000 | 0.0000 | 0.0000 | 0.3723 | 3.3931 | 0.0006 | 0.0000 | 0.1109 | 0.8919 | |
| PDN | 0.3682 | 0.5396 | 1.1532 | 0.0210 | 0.0000 | 0.0000 | 0.0000 | 0.0946 | 15.4508 | 0.0000 | 0.0000 | 0.0000 | 0.0000 | 0.0000 | 0.0000 | |
| PJT | 0.0000 | 0.0000 | 0.0000 | 0.0000 | 0.0000 | 0.0000 | 0.0000 | 0.0000 | 0.0000 | 0.7088 | 2.0137 | 0.0570 | 0.0000 | 0.0000 | 0.0000 | |
| PVP | 0.0000 | 0.1394 | 0.0000 | 0.0000 | 0.0000 | 0.8729 | 1.5337 | 0.0754 | 0.0898 | 0.0000 | 0.0000 | 0.0000 | 0.0000 | 0.0000 | 0.0000 | |
| PVT | 0.0000 | 0.0000 | 0.0000 | 0.0000 | 0.0000 | 0.3111 | 0.1197 | 0.0000 | 0.0000 | 0.0000 | 0.0000 | 0.0000 | 0.0000 | 0.0000 | 0.0000 | |
| REE | 0.0000 | 0.0710 | 0.7584 | 0.0000 | 0.0000 | 0.0153 | 0.2119 | 0.0000 | 0.0000 | 0.0000 | 0.0000 | 0.0000 | 0.0051 | 0.0000 | 0.0000 | |
| SRF | 0.0000 | 0.0000 | 0.0000 | 0.0000 | 0.0000 | 0.0000 | 0.0000 | 0.0000 | 0.0000 | 0.0000 | 0.0000 | 0.0678 | 0.0000 | 0.1577 | 0.7478 | |
| STG | 0.0000 | 0.2018 | 0.0000 | 0.4447 | 0.0426 | 0.0843 | 0.4211 | 0.2271 | 0.0000 | 0.0940 | 0.0000 | 0.0000 | 0.0000 | 0.0000 | 0.0000 | |
| SVI | 0.0719 | 0.1370 | 1.0211 | 0.0000 | 0.0000 | 0.0000 | 0.0000 | 0.2057 | 0.0000 | 0.0000 | 0.0000 | 0.0257 | 0.0000 | 0.0000 | 0.9279 | |
| TCL | 0.1938 | 0.3872 | 0.1284 | 0.0000 | 0.0000 | 0.0000 | 0.0285 | 2.7443 | 1.5947 | 0.0000 | 0.0000 | 0.0000 | 0.0000 | 0.0000 | 0.3155 | |
| TDP | 0.0000 | 0.0000 | 0.0000 | 0.9503 | 0.0000 | 0.0000 | 0.0000 | 0.0000 | 0.0000 | 0.1978 | 0.2057 | 0.0253 | 0.0000 | 0.2074 | 0.4596 | |
| THI | 0.0000 | 0.0000 | 0.0000 | 0.0000 | 0.0000 | 0.0000 | 0.0000 | 0.0000 | 0.0000 | 0.0000 | 0.0000 | 0.0173 | 0.0000 | 0.0000 | 0.1713 | |
| TMS | 0.2265 | 0.3548 | 0.6281 | 1.0850 | 0.0000 | 0.0000 | 0.4061 | 0.0000 | 11.6619 | 0.2476 | 0.0358 | 0.0771 | 0.0000 | 0.0000 | 0.0633 | |
| TV2 | 0.2933 | 0.0638 | 0.6376 | 0.0000 | 0.0000 | 0.0000 | 0.0000 | 1.6085 | 0.0000 | 0.0000 | 0.0000 | 0.0702 | 0.0000 | 0.1821 | 0.9640 | |
| VIP | 0.0000 | 0.0000 | 0.0000 | 0.2985 | 0.0000 | 2.3812 | 0.0000 | 0.0000 | 0.0000 | 0.2243 | 0.0000 | 0.0985 | 0.0271 | 0.0000 | 0.0000 | |
| VOS | 1.8815 | 0.7070 | 0.2134 | 0.0018 | 0.0000 | 0.0000 | 0.0000 | 0.0000 | 0.0000 | 0.0000 | 0.0000 | 0.0000 | 0.0306 | 0.0000 | 0.0000 | |
| VTB | 0.0000 | 0.0000 | 0.0000 | 0.0000 | 0.0000 | 0.9321 | 0.0000 | 2.9076 | 0.0000 | 1.0000 | 0.0000 | 0.0000 | 0.0000 | 0.0000 | 0.3515 | |
| VTO | 0.0000 | 0.0836 | 0.0000 | 0.0000 | 0.2611 | 0.4096 | 0.9576 | 0.0000 | 0.0000 | 0.1574 | 0.9142 | 0.0121 | 0.0253 | 0.0000 | 0.0000 | |
| ARM | 0.0000 | 0.0000 | 0.0000 | 0.0000 | 0.0000 | 0.0000 | 0.1478 | 0.0000 | 0.0000 | 0.0000 | 0.0000 | 0.0304 | 0.0543 | 0.1887 | 0.9640 | |
| BBS | 0.0000 | 0.0000 | 0.0000 | 0.0000 | 0.0278 | 0.0000 | 0.0000 | 0.0000 | 0.0000 | 0.0000 | 0.0000 | 0.0818 | 0.0000 | 0.2378 | 1.0000 | |
| CDN | 0.1259 | 0.4754 | 0.0000 | 0.1187 | 0.0000 | 3.0120 | 0.4914 | 0.5793 | 0.0000 | 0.3360 | 0.0000 | 0.0000 | 0.0584 | 0.0000 | 0.0000 | |
| CIA | 0.0000 | 0.0000 | 0.0000 | 0.0000 | 0.3502 | 0.8927 | 1.6959 | 0.0000 | 0.0000 | 0.0000 | 0.0000 | 1.0000 | 1.0000 | 0.0000 | 0.4235 | |
| CTB | 0.0000 | 0.0000 | 0.0000 | 0.3013 | 0.0000 | 0.3505 | 0.0000 | 0.0000 | 0.0000 | 0.0000 | 0.0000 | 0.0137 | 0.0138 | 0.0334 | 0.0000 | |
| CTT | 0.0972 | 0.0000 | 0.0000 | 0.3963 | 0.0000 | 0.0000 | 0.0000 | 0.0000 | 0.0000 | 0.0000 | 0.0000 | 0.1193 | 0.0000 | 1.0000 | 0.8559 | |
| DL1 | 0.0000 | 0.0000 | 0.0000 | 0.0000 | 0.2950 | 0.0000 | 0.4226 | 0.0000 | 0.0000 | 0.0000 | 0.0000 | 0.1099 | 0.0000 | 0.0000 | 0.0000 | |
| IPA | 1.5301 | 1.1390 | 1.3647 | 0.0000 | 1.8179 | 2.3319 | 3.5723 | 0.0000 | 0.0000 | 0.0000 | 0.0000 | 0.0000 | 0.2226 | 0.0000 | 0.0000 | |
| PBP | 0.0000 | 0.0000 | 0.0000 | 0.0999 | 0.0000 | 0.0000 | 0.0000 | 0.0000 | 0.0000 | 0.1907 | 5.6124 | 0.0714 | 0.0000 | 0.0000 | 0.7838 | |
| PMP | 0.0000 | 0.0000 | 0.0000 | 0.5217 | 0.0000 | 0.0000 | 0.0000 | 0.0000 | 0.0000 | 0.3063 | 0.0000 | 0.0649 | 0.0000 | 0.2715 | 0.8919 | |
| PMS | 0.0612 | 0.0962 | 0.1194 | 0.0000 | 0.0000 | 0.0000 | 0.0000 | 0.0000 | 0.0000 | 0.1163 | 0.0000 | 0.0698 | 0.0000 | 0.0000 | 1.0000 | |
| PRC | 0.0000 | 0.0000 | 0.0000 | 0.0000 | 0.6165 | 0.0000 | 0.0000 | 0.0000 | 0.0000 | 0.2158 | 0.0000 | 0.0675 | 0.0000 | 0.0000 | 0.0000 | |
| PSC | 0.0000 | 0.0000 | 0.0000 | 0.0000 | 0.0000 | 0.0000 | 0.0000 | 0.0000 | 0.3648 | 0.2274 | 1.0252 | 0.0614 | 0.0000 | 0.0000 | 0.0000 | |
| PTS | 0.0000 | 0.0000 | 0.0000 | 0.0000 | 0.0419 | 0.0000 | 0.0000 | 0.0000 | 0.0000 | 0.0000 | 1.3969 | 0.0072 | 0.0132 | 0.0000 | 0.0000 | |
| SDC | 0.0000 | 0.0000 | 0.0000 | 0.0000 | 0.1724 | 0.5871 | 0.5966 | 0.0000 | 0.0000 | 0.0000 | 0.0000 | 0.0000 | 0.3682 | 0.0000 | 0.5677 | |
| SDG | 0.0000 | 0.0000 | 0.0000 | 0.0000 | 0.0000 | 0.0000 | 0.0000 | 0.0000 | 0.0000 | 0.8522 | 0.0000 | 0.0571 | 0.0000 | 0.0000 | 0.8199 | |
| TPP | 0.0000 | 0.0000 | 0.0000 | 0.5275 | 0.0000 | 0.0000 | 0.0000 | 0.0000 | 0.0000 | 0.2138 | 0.0000 | 0.0000 | 0.0000 | 0.1813 | 0.2794 | |
| VBC | 0.2573 | 0.0392 | 0.1027 | 0.0172 | 0.0000 | 0.0000 | 0.0000 | 0.0000 | 0.0000 | 0.0000 | 0.0000 | 0.0788 | 0.0000 | 0.1478 | 0.9640 | |
| VCM | 0.0000 | 0.0000 | 0.0000 | 0.0000 | 0.0000 | 1.0800 | 0.0000 | 2.0084 | 0.0000 | 0.0000 | 0.0000 | 0.0000 | 0.3293 | 0.0000 | 0.7838 | |
| VSM | 0.3289 | 0.3782 | 0.0150 | 0.4456 | 0.0000 | 0.0000 | 0.5247 | 1.1460 | 1.6292 | 0.0000 | 0.0000 | 0.0483 | 0.0000 | 0.0000 | 0.8559 | |

**Table S17. Negative distance from average for all indicators in 2021 based on EDAS**

| Company | Indicator | | | | | | | | | | | | | | |  |
| --- | --- | --- | --- | --- | --- | --- | --- | --- | --- | --- | --- | --- | --- | --- | --- | --- |
|  | I1 | I2 | I3 | I4 | I5 | I6 | I7 | I8 | I9 | I10 | I11 | I12 | I13 | I14 | I15 | |
| CAV | 0.0000 | 0.0256 | 0.0000 | 0.1694 | 0.1245 | 0.4973 | 0.9721 | 0.3247 | 0.9770 | 0.2049 | 0.2876 | 0.0000 | 0.1210 | 0.0000 | 0.0000 | |
| GEX | 0.2255 | 0.2134 | 0.2894 | 0.0000 | 0.0675 | 0.3593 | 0.3416 | 0.4028 | 0.9789 | 0.1589 | 0.3764 | 0.0075 | 0.0419 | 0.0000 | 0.5492 | |
| GMD | 0.1617 | 0.0000 | 0.1361 | 0.0000 | 0.0000 | 0.9014 | 0.4220 | 0.2299 | 0.5555 | 0.2377 | 0.2750 | 0.2625 | 0.0000 | 0.1322 | 0.3691 | |
| GSP | 0.1238 | 0.0454 | 0.3443 | 0.0795 | 0.1176 | 0.0142 | 0.0263 | 0.2002 | 0.0000 | 0.0000 | 0.0000 | 0.0000 | 0.0987 | 0.1023 | 0.7294 | |
| HAH | 0.0000 | 0.0000 | 0.0000 | 0.0000 | 0.0000 | 0.0000 | 0.0000 | 0.0000 | 0.5881 | 0.0000 | 0.0791 | 0.2741 | 0.0461 | 0.1313 | 0.8735 | |
| L10 | 0.2762 | 0.2782 | 0.2956 | 0.3114 | 0.1115 | 0.2311 | 0.0468 | 0.3861 | 0.9649 | 0.2369 | 0.7558 | 0.0000 | 0.1338 | 0.0000 | 0.0000 | |
| MSN | 0.0000 | 0.0000 | 0.0000 | 0.0000 | 0.0000 | 0.3987 | 0.2261 | 0.3806 | 0.9461 | 0.0716 | 0.0000 | 0.1290 | 0.0401 | 0.0498 | 1.0897 | |
| PAC | 0.0000 | 0.0000 | 0.0000 | 0.1238 | 0.0476 | 0.4874 | 1.0000 | 0.2971 | 0.9694 | 0.0000 | 0.0000 | 0.0000 | 0.1128 | 0.0000 | 0.0000 | |
| PDN | 0.0000 | 0.0000 | 0.0000 | 0.0000 | 0.1147 | 0.3100 | 0.0455 | 0.0000 | 0.0000 | 0.1759 | 0.2287 | 0.1927 | 0.0198 | 0.1212 | 0.1529 | |
| PJT | 0.1833 | 0.0976 | 0.3756 | 0.1082 | 0.1427 | 0.8324 | 0.7560 | 0.4158 | 0.7883 | 0.0000 | 0.0000 | 0.0000 | 0.0535 | 0.1297 | 0.9816 | |
| PVP | 0.0170 | 0.0000 | 0.1064 | 0.4839 | 0.0639 | 0.0000 | 0.0000 | 0.0000 | 0.0000 | 0.2343 | 0.7635 | 0.0764 | 0.1082 | 0.1454 | 0.8014 | |
| PVT | 0.1257 | 0.0184 | 0.1253 | 0.1425 | 0.0880 | 0.0000 | 0.0000 | 0.2113 | 0.2067 | 0.1354 | 0.3783 | 0.0238 | 0.0619 | 0.1271 | 0.9816 | |
| REE | 0.0144 | 0.0000 | 0.0000 | 0.1142 | 0.0294 | 0.0000 | 0.0000 | 0.3400 | 0.9606 | 0.2464 | 0.7384 | 0.3146 | 0.0000 | 0.1443 | 1.6301 | |
| SRF | 0.2964 | 0.2614 | 0.4496 | 0.6932 | 0.0106 | 0.5268 | 0.3762 | 0.4304 | 0.9875 | 0.3710 | 0.9566 | 0.0000 | 0.0372 | 0.0000 | 0.0000 | |
| STG | 0.0071 | 0.0000 | 0.0466 | 0.0000 | 0.0000 | 0.0000 | 0.0000 | 0.0000 | 0.5652 | 0.0000 | 0.3870 | 0.0276 | 0.0385 | 0.1352 | 0.0088 | |
| SVI | 0.0000 | 0.0000 | 0.0000 | 0.0292 | 0.2014 | 0.0142 | 0.0679 | 0.0000 | 0.8816 | 0.2464 | 0.6457 | 0.0000 | 0.1001 | 0.0509 | 0.0000 | |
| TCL | 0.0000 | 0.0000 | 0.0000 | 0.0315 | 0.0762 | 0.0931 | 0.0000 | 0.0000 | 0.0000 | 0.2259 | 0.4334 | 0.0310 | 0.0244 | 0.1146 | 0.0000 | |
| TDP | 0.0195 | 0.1264 | 0.2393 | 0.0000 | 0.1754 | 0.6057 | 0.7334 | 0.4309 | 0.9937 | 0.0000 | 0.0000 | 0.0000 | 0.0910 | 0.0000 | 0.0000 | |
| THI | 0.2773 | 0.2050 | 0.1942 | 0.3720 | 0.1726 | 0.2015 | 0.4162 | 0.4207 | 0.9907 | 0.2617 | 0.3378 | 0.0000 | 0.0400 | 0.0275 | 0.0000 | |
| TMS | 0.0000 | 0.0000 | 0.0000 | 0.0000 | 0.0773 | 0.1818 | 0.0000 | 0.0230 | 0.0000 | 0.0000 | 0.0000 | 0.0000 | 0.0926 | 0.1073 | 0.0000 | |
| TV2 | 0.0000 | 0.0000 | 0.0000 | 0.0366 | 0.0985 | 0.4677 | 0.0263 | 0.0000 | 0.7931 | 0.4168 | 0.8735 | 0.0000 | 0.0618 | 0.0000 | 0.0000 | |
| VIP | 0.4562 | 0.3136 | 0.5224 | 0.0000 | 0.3248 | 0.0000 | 0.0128 | 0.4356 | 0.9152 | 0.0000 | 0.2673 | 0.0000 | 0.0000 | 0.1575 | 0.8735 | |
| VOS | 0.0000 | 0.0000 | 0.0000 | 0.0000 | 1.0000 | 0.1621 | 0.2313 | 0.2927 | 0.8349 | 0.3618 | 0.3204 | 0.2150 | 0.0000 | 0.0657 | 1.0536 | |
| VTB | 0.4070 | 0.2566 | 0.4794 | 0.5750 | 0.2497 | 0.0000 | 0.1268 | 0.0000 | 0.9829 | 0.0000 | 0.2461 | 0.1244 | 0.0322 | 0.1369 | 0.0000 | |
| VTO | 0.0691 | 0.0000 | 0.2467 | 0.4272 | 0.0000 | 0.0000 | 0.0000 | 0.2260 | 0.8866 | 0.0000 | 0.0000 | 0.0000 | 0.0000 | 0.1332 | 0.4412 | |
| ARM | 0.2964 | 0.2806 | 0.4334 | 0.0603 | 0.0749 | 0.3297 | 0.0000 | 0.4261 | 0.8611 | 0.0763 | 0.6293 | 0.0000 | 0.0000 | 0.0000 | 0.0000 | |
| BBS | 0.1462 | 0.1972 | 0.2595 | 0.0618 | 0.0000 | 0.6550 | 0.2628 | 0.4311 | 0.8763 | 0.1994 | 0.8340 | 0.0000 | 0.1041 | 0.0000 | 0.0000 | |
| CDN | 0.0000 | 0.0000 | 0.0598 | 0.0000 | 0.0942 | 0.0000 | 0.0000 | 0.0000 | 0.4727 | 0.0000 | 0.1911 | 0.2668 | 0.0000 | 0.1665 | 0.5492 | |
| CIA | 1.0000 | 1.0000 | 1.0000 | 1.0000 | 0.0000 | 0.0000 | 0.0000 | 1.0000 | 0.8873 | 0.3171 | 0.8871 | 0.0000 | 0.0000 | 0.1461 | 0.0000 | |
| CTB | 0.0820 | 0.1150 | 0.1509 | 0.0000 | 0.2023 | 0.0000 | 0.4880 | 0.3784 | 0.9917 | 0.2499 | 0.7056 | 0.0000 | 0.0000 | 0.0000 | 0.3331 | |
| CTT | 0.0000 | 0.2434 | 0.2746 | 0.0000 | 0.1533 | 0.8423 | 0.5534 | 0.4360 | 0.7640 | 0.0988 | 0.2364 | 0.0000 | 0.0658 | 0.0000 | 0.0000 | |
| DL1 | 0.3445 | 0.1918 | 0.4578 | 0.8252 | 0.0000 | 0.2114 | 0.0000 | 0.3379 | 0.9187 | 0.4229 | 0.7162 | 0.0000 | 0.1116 | 0.1135 | 0.4772 | |
| IPA | 0.0000 | 0.0000 | 0.0000 | 0.0492 | 0.0000 | 0.0000 | 0.0000 | 0.1957 | 0.7838 | 0.1103 | 0.4276 | 0.4985 | 0.0000 | 0.1397 | 1.7742 | |
| PBP | 0.1525 | 0.0160 | 0.3304 | 0.0000 | 0.1183 | 0.0340 | 0.5528 | 0.0263 | 0.9083 | 0.0000 | 0.0000 | 0.0000 | 0.0456 | 0.0903 | 0.0000 | |
| PMP | 0.1136 | 0.1924 | 0.2573 | 0.0000 | 0.1577 | 0.6254 | 0.6959 | 0.4228 | 0.9346 | 0.0000 | 0.1129 | 0.0000 | 0.0475 | 0.0000 | 0.0000 | |
| PMS | 0.0000 | 0.0000 | 0.0000 | 0.1551 | 0.0480 | 0.4874 | 0.4431 | 0.0820 | 0.7778 | 0.0000 | 0.2731 | 0.0000 | 0.0536 | 0.0226 | 0.0000 | |
| PRC | 0.3192 | 0.2206 | 0.3121 | 0.1740 | 0.0000 | 0.6648 | 0.3004 | 0.4310 | 0.6511 | 0.0000 | 0.5077 | 0.0000 | 0.0273 | 0.0719 | 0.2610 | |
| PSC | 0.2560 | 0.1816 | 0.3523 | 0.1058 | 0.1848 | 1.0000 | 0.5841 | 0.3922 | 0.0000 | 0.0000 | 0.0000 | 0.0000 | 0.0853 | 0.1077 | 1.0897 | |
| PTS | 0.0504 | 0.1072 | 0.1446 | 0.0315 | 0.0000 | 0.9409 | 0.6684 | 0.4108 | 0.7630 | 0.0724 | 0.0000 | 0.0000 | 0.0000 | 0.0547 | 0.9095 | |
| SDC | 0.3273 | 0.1990 | 0.4064 | 0.2888 | 0.0000 | 0.0000 | 0.0000 | 0.2143 | 1.0000 | 0.1823 | 1.0000 | 0.0903 | 0.0000 | 0.0924 | 0.0000 | |
| SDG | 0.3350 | 0.2200 | 0.1674 | 0.3797 | 0.2519 | 0.4480 | 0.5844 | 0.4064 | 0.8853 | 0.0000 | 0.3494 | 0.0000 | 0.0626 | 0.0414 | 0.0000 | |
| TPP | 0.3816 | 0.3076 | 0.4958 | 0.0000 | 0.1832 | 0.5663 | 0.5046 | 0.4601 | 0.9574 | 0.0000 | 0.4179 | 0.0441 | 0.0800 | 0.0000 | 0.0000 | |
| VBC | 0.0000 | 0.0000 | 0.0000 | 0.0000 | 0.1122 | 0.4283 | 0.2342 | 0.3535 | 0.8795 | 0.1094 | 0.6274 | 0.0000 | 0.1119 | 0.0000 | 0.0000 | |
| VCM | 0.4418 | 0.2944 | 0.4850 | 0.0619 | 0.3284 | 0.0000 | 0.3571 | 0.0000 | 0.1256 | 0.2250 | 0.6882 | 0.0714 | 0.0000 | 0.1408 | 0.0000 | |
| VSM | 0.0000 | 0.0000 | 0.0000 | 0.0000 | 0.0625 | 0.1424 | 0.0000 | 0.0000 | 0.0000 | 0.1210 | 0.4594 | 0.0000 | 0.0730 | 0.0589 | 0.0000 | |

**Table S18. Weighted positive distance for all indicators in 2021 based on EDAS.**

| Company | Indicator | | | | | | | | | | | | | | |  |
| --- | --- | --- | --- | --- | --- | --- | --- | --- | --- | --- | --- | --- | --- | --- | --- | --- |
|  | I1 | I2 | I3 | I4 | I5 | I6 | I7 | I8 | I9 | I10 | I11 | I12 | I13 | I14 | I15 | |
| CAV | 0.0096 | 0.0000 | 0.0456 | 0.0000 | 0.0000 | 0.0000 | 0.0000 | 0.0000 | 0.0000 | 0.0000 | 0.0000 | 0.0027 | 0.0000 | 0.0140 | 0.0712 | |
| GEX | 0.0000 | 0.0000 | 0.0000 | 0.0524 | 0.0000 | 0.0000 | 0.0000 | 0.0000 | 0.0000 | 0.0000 | 0.0000 | 0.0000 | 0.0000 | 0.0001 | 0.0000 | |
| GMD | 0.0000 | 0.0001 | 0.0000 | 0.0132 | 0.0002 | 0.0000 | 0.0000 | 0.0000 | 0.0000 | 0.0000 | 0.0000 | 0.0000 | 0.0049 | 0.0000 | 0.0000 | |
| GSP | 0.0000 | 0.0000 | 0.0000 | 0.0000 | 0.0000 | 0.0000 | 0.0000 | 0.0000 | 0.0277 | 0.0072 | 0.0093 | 0.0041 | 0.0000 | 0.0000 | 0.0000 | |
| HAH | 0.0247 | 0.0270 | 0.0897 | 0.0579 | 0.0138 | 0.0310 | 0.0545 | 0.0206 | 0.0000 | 0.0219 | 0.0000 | 0.0000 | 0.0000 | 0.0000 | 0.0000 | |
| L10 | 0.0000 | 0.0000 | 0.0000 | 0.0000 | 0.0000 | 0.0000 | 0.0000 | 0.0000 | 0.0000 | 0.0000 | 0.0000 | 0.0058 | 0.0000 | 0.0194 | 0.0095 | |
| MSN | 0.0210 | 0.0029 | 0.0676 | 0.0031 | 0.0717 | 0.0000 | 0.0000 | 0.0000 | 0.0000 | 0.0000 | 0.2102 | 0.0000 | 0.0000 | 0.0000 | 0.0000 | |
| PAC | 0.0146 | 0.0030 | 0.0120 | 0.0000 | 0.0000 | 0.0000 | 0.0000 | 0.0000 | 0.0000 | 0.0351 | 0.2643 | 0.0000 | 0.0000 | 0.0069 | 0.0849 | |
| PDN | 0.0175 | 0.0224 | 0.0752 | 0.0016 | 0.0000 | 0.0000 | 0.0000 | 0.0076 | 1.0345 | 0.0000 | 0.0000 | 0.0000 | 0.0000 | 0.0000 | 0.0000 | |
| PJT | 0.0000 | 0.0000 | 0.0000 | 0.0000 | 0.0000 | 0.0000 | 0.0000 | 0.0000 | 0.0000 | 0.0667 | 0.1569 | 0.0023 | 0.0000 | 0.0000 | 0.0000 | |
| PVP | 0.0000 | 0.0058 | 0.0000 | 0.0000 | 0.0000 | 0.0677 | 0.0902 | 0.0061 | 0.0060 | 0.0000 | 0.0000 | 0.0000 | 0.0000 | 0.0000 | 0.0000 | |
| PVT | 0.0000 | 0.0000 | 0.0000 | 0.0000 | 0.0000 | 0.0241 | 0.0070 | 0.0000 | 0.0000 | 0.0000 | 0.0000 | 0.0000 | 0.0000 | 0.0000 | 0.0000 | |
| REE | 0.0000 | 0.0030 | 0.0494 | 0.0000 | 0.0000 | 0.0012 | 0.0125 | 0.0000 | 0.0000 | 0.0000 | 0.0000 | 0.0000 | 0.0003 | 0.0000 | 0.0000 | |
| SRF | 0.0000 | 0.0000 | 0.0000 | 0.0000 | 0.0000 | 0.0000 | 0.0000 | 0.0000 | 0.0000 | 0.0000 | 0.0000 | 0.0027 | 0.0000 | 0.0098 | 0.0712 | |
| STG | 0.0000 | 0.0084 | 0.0000 | 0.0339 | 0.0022 | 0.0065 | 0.0248 | 0.0183 | 0.0000 | 0.0088 | 0.0000 | 0.0000 | 0.0000 | 0.0000 | 0.0000 | |
| SVI | 0.0034 | 0.0057 | 0.0666 | 0.0000 | 0.0000 | 0.0000 | 0.0000 | 0.0166 | 0.0000 | 0.0000 | 0.0000 | 0.0010 | 0.0000 | 0.0000 | 0.0884 | |
| TCL | 0.0092 | 0.0161 | 0.0084 | 0.0000 | 0.0000 | 0.0000 | 0.0017 | 0.2211 | 0.1068 | 0.0000 | 0.0000 | 0.0000 | 0.0000 | 0.0000 | 0.0300 | |
| TDP | 0.0000 | 0.0000 | 0.0000 | 0.0724 | 0.0000 | 0.0000 | 0.0000 | 0.0000 | 0.0000 | 0.0186 | 0.0160 | 0.0010 | 0.0000 | 0.0129 | 0.0438 | |
| THI | 0.0000 | 0.0000 | 0.0000 | 0.0000 | 0.0000 | 0.0000 | 0.0000 | 0.0000 | 0.0000 | 0.0000 | 0.0000 | 0.0007 | 0.0000 | 0.0000 | 0.0163 | |
| TMS | 0.0108 | 0.0148 | 0.0410 | 0.0827 | 0.0000 | 0.0000 | 0.0239 | 0.0000 | 0.7808 | 0.0233 | 0.0028 | 0.0031 | 0.0000 | 0.0000 | 0.0060 | |
| TV2 | 0.0139 | 0.0027 | 0.0416 | 0.0000 | 0.0000 | 0.0000 | 0.0000 | 0.1296 | 0.0000 | 0.0000 | 0.0000 | 0.0028 | 0.0000 | 0.0113 | 0.0918 | |
| VIP | 0.0000 | 0.0000 | 0.0000 | 0.0228 | 0.0000 | 0.1845 | 0.0000 | 0.0000 | 0.0000 | 0.0211 | 0.0000 | 0.0039 | 0.0017 | 0.0000 | 0.0000 | |
| VOS | 0.0894 | 0.0294 | 0.0139 | 0.0001 | 0.0000 | 0.0000 | 0.0000 | 0.0000 | 0.0000 | 0.0000 | 0.0000 | 0.0000 | 0.0020 | 0.0000 | 0.0000 | |
| VTB | 0.0000 | 0.0000 | 0.0000 | 0.0000 | 0.0000 | 0.0722 | 0.0000 | 0.2343 | 0.0000 | 0.0942 | 0.0000 | 0.0000 | 0.0000 | 0.0000 | 0.0335 | |
| VTO | 0.0000 | 0.0035 | 0.0000 | 0.0000 | 0.0136 | 0.0317 | 0.0563 | 0.0000 | 0.0000 | 0.0148 | 0.0712 | 0.0005 | 0.0016 | 0.0000 | 0.0000 | |
| ARM | 0.0000 | 0.0000 | 0.0000 | 0.0000 | 0.0000 | 0.0000 | 0.0087 | 0.0000 | 0.0000 | 0.0000 | 0.0000 | 0.0012 | 0.0035 | 0.0117 | 0.0918 | |
| BBS | 0.0000 | 0.0000 | 0.0000 | 0.0000 | 0.0015 | 0.0000 | 0.0000 | 0.0000 | 0.0000 | 0.0000 | 0.0000 | 0.0032 | 0.0000 | 0.0148 | 0.0952 | |
| CDN | 0.0060 | 0.0198 | 0.0000 | 0.0090 | 0.0000 | 0.2334 | 0.0289 | 0.0467 | 0.0000 | 0.0316 | 0.0000 | 0.0000 | 0.0038 | 0.0000 | 0.0000 | |
| CIA | 0.0000 | 0.0000 | 0.0000 | 0.0000 | 0.0183 | 0.0692 | 0.0997 | 0.0000 | 0.0000 | 0.0000 | 0.0000 | 0.0397 | 0.0644 | 0.0000 | 0.0403 | |
| CTB | 0.0000 | 0.0000 | 0.0000 | 0.0230 | 0.0000 | 0.0272 | 0.0000 | 0.0000 | 0.0000 | 0.0000 | 0.0000 | 0.0005 | 0.0009 | 0.0021 | 0.0000 | |
| CTT | 0.0046 | 0.0000 | 0.0000 | 0.0302 | 0.0000 | 0.0000 | 0.0000 | 0.0000 | 0.0000 | 0.0000 | 0.0000 | 0.0047 | 0.0000 | 0.0620 | 0.0815 | |
| DL1 | 0.0000 | 0.0000 | 0.0000 | 0.0000 | 0.0154 | 0.0000 | 0.0249 | 0.0000 | 0.0000 | 0.0000 | 0.0000 | 0.0044 | 0.0000 | 0.0000 | 0.0000 | |
| IPA | 0.0727 | 0.0474 | 0.0890 | 0.0000 | 0.0950 | 0.1807 | 0.2101 | 0.0000 | 0.0000 | 0.0000 | 0.0000 | 0.0000 | 0.0143 | 0.0000 | 0.0000 | |
| PBP | 0.0000 | 0.0000 | 0.0000 | 0.0076 | 0.0000 | 0.0000 | 0.0000 | 0.0000 | 0.0000 | 0.0180 | 0.4372 | 0.0028 | 0.0000 | 0.0000 | 0.0746 | |
| PMP | 0.0000 | 0.0000 | 0.0000 | 0.0398 | 0.0000 | 0.0000 | 0.0000 | 0.0000 | 0.0000 | 0.0288 | 0.0000 | 0.0026 | 0.0000 | 0.0168 | 0.0849 | |
| PMS | 0.0029 | 0.0040 | 0.0078 | 0.0000 | 0.0000 | 0.0000 | 0.0000 | 0.0000 | 0.0000 | 0.0110 | 0.0000 | 0.0028 | 0.0000 | 0.0000 | 0.0952 | |
| PRC | 0.0000 | 0.0000 | 0.0000 | 0.0000 | 0.0322 | 0.0000 | 0.0000 | 0.0000 | 0.0000 | 0.0203 | 0.0000 | 0.0027 | 0.0000 | 0.0000 | 0.0000 | |
| PSC | 0.0000 | 0.0000 | 0.0000 | 0.0000 | 0.0000 | 0.0000 | 0.0000 | 0.0000 | 0.0244 | 0.0214 | 0.0799 | 0.0024 | 0.0000 | 0.0000 | 0.0000 | |
| PTS | 0.0000 | 0.0000 | 0.0000 | 0.0000 | 0.0022 | 0.0000 | 0.0000 | 0.0000 | 0.0000 | 0.0000 | 0.1088 | 0.0003 | 0.0009 | 0.0000 | 0.0000 | |
| SDC | 0.0000 | 0.0000 | 0.0000 | 0.0000 | 0.0090 | 0.0455 | 0.0351 | 0.0000 | 0.0000 | 0.0000 | 0.0000 | 0.0000 | 0.0237 | 0.0000 | 0.0541 | |
| SDG | 0.0000 | 0.0000 | 0.0000 | 0.0000 | 0.0000 | 0.0000 | 0.0000 | 0.0000 | 0.0000 | 0.0802 | 0.0000 | 0.0023 | 0.0000 | 0.0000 | 0.0781 | |
| TPP | 0.0000 | 0.0000 | 0.0000 | 0.0402 | 0.0000 | 0.0000 | 0.0000 | 0.0000 | 0.0000 | 0.0201 | 0.0000 | 0.0000 | 0.0000 | 0.0112 | 0.0266 | |
| VBC | 0.0122 | 0.0016 | 0.0067 | 0.0013 | 0.0000 | 0.0000 | 0.0000 | 0.0000 | 0.0000 | 0.0000 | 0.0000 | 0.0031 | 0.0000 | 0.0092 | 0.0918 | |
| VCM | 0.0000 | 0.0000 | 0.0000 | 0.0000 | 0.0000 | 0.0837 | 0.0000 | 0.1618 | 0.0000 | 0.0000 | 0.0000 | 0.0000 | 0.0212 | 0.0000 | 0.0746 | |
| VSM | 0.0156 | 0.0157 | 0.0010 | 0.0340 | 0.0000 | 0.0000 | 0.0309 | 0.0923 | 0.1091 | 0.0000 | 0.0000 | 0.0019 | 0.0000 | 0.0000 | 0.0815 | |

**Table S19. Weighted negative distance for all indicators in 2021 based on EDAS.**

| Company | Indicator | | | | | | | | | | | | | | |  |
| --- | --- | --- | --- | --- | --- | --- | --- | --- | --- | --- | --- | --- | --- | --- | --- | --- |
|  | I1 | I2 | I3 | I4 | I5 | I6 | I7 | I8 | I9 | I10 | I11 | I12 | I13 | I14 | I15 | |
| CAV | 0.0000 | 0.0011 | 0.0000 | 0.0129 | 0.0065 | 0.0385 | 0.0572 | 0.0262 | 0.0654 | 0.0193 | 0.0224 | 0.0000 | 0.0078 | 0.0000 | 0.0000 | |
| GEX | 0.0107 | 0.0089 | 0.0189 | 0.0000 | 0.0035 | 0.0278 | 0.0201 | 0.0325 | 0.0655 | 0.0150 | 0.0293 | 0.0003 | 0.0027 | 0.0000 | 0.0523 | |
| GMD | 0.0077 | 0.0000 | 0.0089 | 0.0000 | 0.0000 | 0.0699 | 0.0248 | 0.0185 | 0.0372 | 0.0224 | 0.0214 | 0.0104 | 0.0000 | 0.0082 | 0.0351 | |
| GSP | 0.0059 | 0.0019 | 0.0224 | 0.0061 | 0.0061 | 0.0011 | 0.0015 | 0.0161 | 0.0000 | 0.0000 | 0.0000 | 0.0000 | 0.0064 | 0.0063 | 0.0695 | |
| HAH | 0.0000 | 0.0000 | 0.0000 | 0.0000 | 0.0000 | 0.0000 | 0.0000 | 0.0000 | 0.0394 | 0.0000 | 0.0062 | 0.0109 | 0.0030 | 0.0081 | 0.0832 | |
| L10 | 0.0131 | 0.0116 | 0.0193 | 0.0237 | 0.0058 | 0.0179 | 0.0028 | 0.0311 | 0.0646 | 0.0223 | 0.0589 | 0.0000 | 0.0086 | 0.0000 | 0.0000 | |
| MSN | 0.0000 | 0.0000 | 0.0000 | 0.0000 | 0.0000 | 0.0309 | 0.0133 | 0.0307 | 0.0633 | 0.0067 | 0.0000 | 0.0051 | 0.0026 | 0.0031 | 0.1038 | |
| PAC | 0.0000 | 0.0000 | 0.0000 | 0.0094 | 0.0025 | 0.0378 | 0.0588 | 0.0239 | 0.0649 | 0.0000 | 0.0000 | 0.0000 | 0.0073 | 0.0000 | 0.0000 | |
| PDN | 0.0000 | 0.0000 | 0.0000 | 0.0000 | 0.0060 | 0.0240 | 0.0027 | 0.0000 | 0.0000 | 0.0166 | 0.0178 | 0.0076 | 0.0013 | 0.0075 | 0.0146 | |
| PJT | 0.0087 | 0.0041 | 0.0245 | 0.0082 | 0.0075 | 0.0645 | 0.0445 | 0.0335 | 0.0528 | 0.0000 | 0.0000 | 0.0000 | 0.0034 | 0.0080 | 0.0935 | |
| PVP | 0.0008 | 0.0000 | 0.0069 | 0.0369 | 0.0033 | 0.0000 | 0.0000 | 0.0000 | 0.0000 | 0.0221 | 0.0595 | 0.0030 | 0.0070 | 0.0090 | 0.0763 | |
| PVT | 0.0060 | 0.0008 | 0.0082 | 0.0109 | 0.0046 | 0.0000 | 0.0000 | 0.0170 | 0.0138 | 0.0127 | 0.0295 | 0.0009 | 0.0040 | 0.0079 | 0.0935 | |
| REE | 0.0007 | 0.0000 | 0.0000 | 0.0087 | 0.0015 | 0.0000 | 0.0000 | 0.0274 | 0.0643 | 0.0232 | 0.0575 | 0.0125 | 0.0000 | 0.0090 | 0.1552 | |
| SRF | 0.0141 | 0.0109 | 0.0293 | 0.0528 | 0.0006 | 0.0408 | 0.0221 | 0.0347 | 0.0661 | 0.0349 | 0.0745 | 0.0000 | 0.0024 | 0.0000 | 0.0000 | |
| STG | 0.0003 | 0.0000 | 0.0030 | 0.0000 | 0.0000 | 0.0000 | 0.0000 | 0.0000 | 0.0378 | 0.0000 | 0.0302 | 0.0011 | 0.0025 | 0.0084 | 0.0008 | |
| SVI | 0.0000 | 0.0000 | 0.0000 | 0.0022 | 0.0105 | 0.0011 | 0.0040 | 0.0000 | 0.0590 | 0.0232 | 0.0503 | 0.0000 | 0.0064 | 0.0032 | 0.0000 | |
| TCL | 0.0000 | 0.0000 | 0.0000 | 0.0024 | 0.0040 | 0.0072 | 0.0000 | 0.0000 | 0.0000 | 0.0213 | 0.0338 | 0.0012 | 0.0016 | 0.0071 | 0.0000 | |
| TDP | 0.0009 | 0.0053 | 0.0156 | 0.0000 | 0.0092 | 0.0469 | 0.0431 | 0.0347 | 0.0665 | 0.0000 | 0.0000 | 0.0000 | 0.0059 | 0.0000 | 0.0000 | |
| THI | 0.0132 | 0.0085 | 0.0127 | 0.0284 | 0.0090 | 0.0156 | 0.0245 | 0.0339 | 0.0663 | 0.0246 | 0.0263 | 0.0000 | 0.0026 | 0.0017 | 0.0000 | |
| TMS | 0.0000 | 0.0000 | 0.0000 | 0.0000 | 0.0040 | 0.0141 | 0.0000 | 0.0019 | 0.0000 | 0.0000 | 0.0000 | 0.0000 | 0.0060 | 0.0067 | 0.0000 | |
| TV2 | 0.0000 | 0.0000 | 0.0000 | 0.0028 | 0.0051 | 0.0362 | 0.0015 | 0.0000 | 0.0531 | 0.0392 | 0.0681 | 0.0000 | 0.0040 | 0.0000 | 0.0000 | |
| VIP | 0.0217 | 0.0130 | 0.0341 | 0.0000 | 0.0170 | 0.0000 | 0.0008 | 0.0351 | 0.0613 | 0.0000 | 0.0208 | 0.0000 | 0.0000 | 0.0098 | 0.0832 | |
| VOS | 0.0000 | 0.0000 | 0.0000 | 0.0000 | 0.0522 | 0.0126 | 0.0136 | 0.0236 | 0.0559 | 0.0341 | 0.0250 | 0.0085 | 0.0000 | 0.0041 | 0.1003 | |
| VTB | 0.0193 | 0.0107 | 0.0313 | 0.0438 | 0.0130 | 0.0000 | 0.0075 | 0.0000 | 0.0658 | 0.0000 | 0.0192 | 0.0049 | 0.0021 | 0.0085 | 0.0000 | |
| VTO | 0.0033 | 0.0000 | 0.0161 | 0.0326 | 0.0000 | 0.0000 | 0.0000 | 0.0182 | 0.0594 | 0.0000 | 0.0000 | 0.0000 | 0.0000 | 0.0083 | 0.0420 | |
| ARM | 0.0141 | 0.0117 | 0.0283 | 0.0046 | 0.0039 | 0.0256 | 0.0000 | 0.0343 | 0.0577 | 0.0072 | 0.0490 | 0.0000 | 0.0000 | 0.0000 | 0.0000 | |
| BBS | 0.0070 | 0.0082 | 0.0169 | 0.0047 | 0.0000 | 0.0508 | 0.0155 | 0.0347 | 0.0587 | 0.0188 | 0.0650 | 0.0000 | 0.0067 | 0.0000 | 0.0000 | |
| CDN | 0.0000 | 0.0000 | 0.0039 | 0.0000 | 0.0049 | 0.0000 | 0.0000 | 0.0000 | 0.0317 | 0.0000 | 0.0149 | 0.0106 | 0.0000 | 0.0103 | 0.0523 | |
| CIA | 0.0475 | 0.0416 | 0.0652 | 0.0762 | 0.0000 | 0.0000 | 0.0000 | 0.0806 | 0.0594 | 0.0299 | 0.0691 | 0.0000 | 0.0000 | 0.0091 | 0.0000 | |
| CTB | 0.0039 | 0.0048 | 0.0098 | 0.0000 | 0.0106 | 0.0000 | 0.0287 | 0.0305 | 0.0664 | 0.0235 | 0.0550 | 0.0000 | 0.0000 | 0.0000 | 0.0317 | |
| CTT | 0.0000 | 0.0101 | 0.0179 | 0.0000 | 0.0080 | 0.0653 | 0.0325 | 0.0351 | 0.0512 | 0.0093 | 0.0184 | 0.0000 | 0.0042 | 0.0000 | 0.0000 | |
| DL1 | 0.0164 | 0.0080 | 0.0299 | 0.0629 | 0.0000 | 0.0164 | 0.0000 | 0.0272 | 0.0615 | 0.0398 | 0.0558 | 0.0000 | 0.0072 | 0.0070 | 0.0454 | |
| IPA | 0.0000 | 0.0000 | 0.0000 | 0.0037 | 0.0000 | 0.0000 | 0.0000 | 0.0158 | 0.0525 | 0.0104 | 0.0333 | 0.0198 | 0.0000 | 0.0087 | 0.1689 | |
| PBP | 0.0072 | 0.0007 | 0.0215 | 0.0000 | 0.0062 | 0.0026 | 0.0325 | 0.0021 | 0.0608 | 0.0000 | 0.0000 | 0.0000 | 0.0029 | 0.0056 | 0.0000 | |
| PMP | 0.0054 | 0.0080 | 0.0168 | 0.0000 | 0.0082 | 0.0485 | 0.0409 | 0.0341 | 0.0626 | 0.0000 | 0.0088 | 0.0000 | 0.0031 | 0.0000 | 0.0000 | |
| PMS | 0.0000 | 0.0000 | 0.0000 | 0.0118 | 0.0025 | 0.0378 | 0.0261 | 0.0066 | 0.0521 | 0.0000 | 0.0213 | 0.0000 | 0.0035 | 0.0014 | 0.0000 | |
| PRC | 0.0152 | 0.0092 | 0.0203 | 0.0133 | 0.0000 | 0.0515 | 0.0177 | 0.0347 | 0.0436 | 0.0000 | 0.0396 | 0.0000 | 0.0018 | 0.0045 | 0.0249 | |
| PSC | 0.0122 | 0.0076 | 0.0230 | 0.0081 | 0.0097 | 0.0775 | 0.0344 | 0.0316 | 0.0000 | 0.0000 | 0.0000 | 0.0000 | 0.0055 | 0.0067 | 0.1038 | |
| PTS | 0.0024 | 0.0045 | 0.0094 | 0.0024 | 0.0000 | 0.0729 | 0.0393 | 0.0331 | 0.0511 | 0.0068 | 0.0000 | 0.0000 | 0.0000 | 0.0034 | 0.0866 | |
| SDC | 0.0156 | 0.0083 | 0.0265 | 0.0220 | 0.0000 | 0.0000 | 0.0000 | 0.0173 | 0.0670 | 0.0172 | 0.0779 | 0.0036 | 0.0000 | 0.0057 | 0.0000 | |
| SDG | 0.0159 | 0.0092 | 0.0109 | 0.0289 | 0.0132 | 0.0347 | 0.0344 | 0.0327 | 0.0593 | 0.0000 | 0.0272 | 0.0000 | 0.0040 | 0.0026 | 0.0000 | |
| TPP | 0.0181 | 0.0128 | 0.0323 | 0.0000 | 0.0096 | 0.0439 | 0.0297 | 0.0371 | 0.0641 | 0.0000 | 0.0326 | 0.0018 | 0.0052 | 0.0000 | 0.0000 | |
| VBC | 0.0000 | 0.0000 | 0.0000 | 0.0000 | 0.0059 | 0.0332 | 0.0138 | 0.0285 | 0.0589 | 0.0103 | 0.0489 | 0.0000 | 0.0072 | 0.0000 | 0.0000 | |
| VCM | 0.0210 | 0.0122 | 0.0316 | 0.0047 | 0.0172 | 0.0000 | 0.0210 | 0.0000 | 0.0084 | 0.0212 | 0.0536 | 0.0028 | 0.0000 | 0.0087 | 0.0000 | |
| VSM | 0.0000 | 0.0000 | 0.0000 | 0.0000 | 0.0033 | 0.0110 | 0.0000 | 0.0000 | 0.0000 | 0.0114 | 0.0358 | 0.0000 | 0.0047 | 0.0037 | 0.0000 | |

**Table S20. The normalized sum of the weighted positive and negative distance from the average, the appraisal score, and rank in 2021 based on EDAS.**

| Company | NSP | NSN | AS | Rank |
| --- | --- | --- | --- | --- |
| CAV | 0.1235 | 0.4624 | 0.2930 | 27 |
| GEX | 0.0453 | 0.3992 | 0.2223 | 39 |
| GMD | 0.0158 | 0.4473 | 0.2315 | 34 |
| GSP | 0.0417 | 0.7004 | 0.3710 | 18 |
| HAH | 0.2944 | 0.6851 | 0.4898 | 7 |
| L10 | 0.0299 | 0.4155 | 0.2227 | 37 |
| MSN | 0.3250 | 0.4578 | 0.3914 | 16 |
| PAC | 0.3632 | 0.5724 | 0.4678 | 9 |
| PDN | 1.0000 | 0.7951 | 0.8975 | 1 |
| PJT | 0.1949 | 0.2620 | 0.2284 | 35 |
| PVP | 0.1517 | 0.5302 | 0.3409 | 19 |
| PVT | 0.0269 | 0.5617 | 0.2943 | 26 |
| REE | 0.0573 | 0.2477 | 0.1525 | 42 |
| SRF | 0.0722 | 0.1991 | 0.1357 | 44 |
| STG | 0.0888 | 0.8241 | 0.4565 | 10 |
| SVI | 0.1567 | 0.6657 | 0.4112 | 13 |
| TCL | 0.3394 | 0.8359 | 0.5876 | 4 |
| TDP | 0.1421 | 0.5232 | 0.3327 | 21 |
| THI | 0.0147 | 0.4414 | 0.2280 | 36 |
| TMS | 0.8535 | 0.9319 | 0.8927 | 2 |
| TV2 | 0.2534 | 0.5610 | 0.4072 | 14 |
| VIP | 0.2020 | 0.3801 | 0.2910 | 28 |
| VOS | 0.1164 | 0.3107 | 0.2135 | 41 |
| VTB | 0.3746 | 0.5276 | 0.4511 | 11 |
| VTO | 0.1668 | 0.6243 | 0.3956 | 15 |
| ARM | 0.1009 | 0.5063 | 0.3036 | 24 |
| BBS | 0.0990 | 0.4006 | 0.2498 | 30 |
| CDN | 0.3272 | 0.7313 | 0.5293 | 6 |
| CIA | 0.2862 | 0.0000 | 0.1431 | 43 |
| CTB | 0.0463 | 0.4465 | 0.2464 | 31 |
| CTT | 0.1580 | 0.4732 | 0.3156 | 23 |
| DL1 | 0.0385 | 0.2112 | 0.1248 | 45 |
| IPA | 0.6120 | 0.3458 | 0.4789 | 8 |
| PBP | 0.4662 | 0.7027 | 0.5845 | 5 |
| PMP | 0.1492 | 0.5062 | 0.3277 | 22 |
| PMS | 0.1067 | 0.6594 | 0.3831 | 17 |
| PRC | 0.0476 | 0.4231 | 0.2353 | 33 |
| PSC | 0.1106 | 0.3317 | 0.2212 | 40 |
| PTS | 0.0968 | 0.3482 | 0.2225 | 38 |
| SDC | 0.1444 | 0.4547 | 0.2996 | 25 |
| SDG | 0.1386 | 0.4295 | 0.2840 | 29 |
| TPP | 0.0847 | 0.4002 | 0.2425 | 32 |
| VBC | 0.1087 | 0.5683 | 0.3385 | 20 |
| VCM | 0.2946 | 0.5768 | 0.4357 | 12 |
| VSM | 0.3296 | 0.8541 | 0.5919 | 3 |

**Table S21. The criteria's average solution in 2022 based on EDAS.**

| Company | Indicator | | | | | | | | | | | | | | |  |
| --- | --- | --- | --- | --- | --- | --- | --- | --- | --- | --- | --- | --- | --- | --- | --- | --- |
|  | I1 | I2 | I3 | I4 | I5 | I6 | I7 | I8 | I9 | I10 | I11 | I12 | I13 | I14 | I15 | |
| CAV | 0.4042 | 0.2150 | 0.2339 | 0.2980 | 0.1284 | 0.0265 | 0.0067 | 0.0539 | 0.0023 | 0.8405 | 0.0754 | 0.2166 | 1.0000 | 0.8471 | 0.1098 | |
| GEX | 0.2454 | 0.1252 | 0.0967 | 0.3144 | 0.1212 | 0.0208 | 0.0163 | 0.0350 | 0.0013 | 0.8965 | 0.0513 | 0.3968 | 0.8802 | 0.9126 | 0.5366 | |
| GMD | 0.3394 | 0.2384 | 0.1598 | 0.3534 | 0.1364 | 0.0062 | 0.0231 | 0.0970 | 0.0403 | 0.9545 | 0.0414 | 0.8345 | 0.6374 | 0.9590 | 0.4390 | |
| GSP | 0.3263 | 0.2010 | 0.1177 | 0.3181 | 0.1351 | 0.0381 | 0.0228 | 0.0519 | 0.0998 | 0.8300 | 0.0631 | 0.1924 | 0.9496 | 0.9542 | 0.6829 | |
| HAH | 0.5120 | 0.4067 | 0.3440 | 0.5293 | 0.1312 | 0.0549 | 0.0583 | 0.1866 | 0.0393 | 0.7656 | 0.0731 | 0.8396 | 0.9287 | 0.9689 | 0.6951 | |
| L10 | 0.2853 | 0.1371 | 0.1185 | 0.2499 | 0.1323 | 0.0389 | 0.0479 | 0.0449 | 0.0042 | 0.9264 | 0.0149 | 0.0957 | 0.9777 | 0.7617 | 0.3537 | |
| MSN | 0.3048 | 0.1548 | 0.1423 | 0.2019 | 0.1133 | 0.0022 | 0.0131 | 0.0316 | 0.0036 | 0.8994 | 0.1612 | 0.5335 | 0.8843 | 0.8056 | 0.4512 | |
| PAC | 0.3752 | 0.2103 | 0.1551 | 0.2997 | 0.1217 | 0.0208 | 0.0006 | 0.0515 | 0.0037 | 0.6311 | 0.2173 | 0.2888 | 0.9790 | 0.8300 | 0.0244 | |
| PDN | 0.4778 | 0.4043 | 0.3439 | 0.3453 | 0.1332 | 0.0407 | 0.0424 | 0.1733 | 1.0000 | 0.8978 | 0.0552 | 0.6542 | 0.8805 | 0.9646 | 0.3415 | |
| PJT | 0.2931 | 0.1814 | 0.1058 | 0.3927 | 0.1205 | 0.0137 | 0.0113 | 0.0386 | 0.0207 | 0.6117 | 0.2725 | 0.1714 | 0.9225 | 0.9645 | 0.5122 | |
| PVP | 0.3418 | 0.2459 | 0.1377 | 0.3457 | 0.1247 | 0.0796 | 0.0842 | 0.1515 | 0.1464 | 0.9345 | 0.0147 | 0.1537 | 0.9720 | 0.9650 | 0.4390 | |
| PVT | 0.3246 | 0.2097 | 0.1437 | 0.3522 | 0.1309 | 0.0611 | 0.0371 | 0.0735 | 0.0658 | 0.8555 | 0.0434 | 0.3633 | 0.9006 | 0.9616 | 0.6098 | |
| REE | 0.3548 | 0.2358 | 0.2533 | 0.5181 | 0.1273 | 0.0628 | 0.0551 | 0.0560 | 0.0043 | 0.8902 | 0.0190 | 0.8733 | 0.8518 | 0.9803 | 0.8659 | |
| SRF | 0.0000 | 0.0000 | 0.0000 | 0.3777 | 0.0000 | 0.0199 | 0.0265 | 0.0000 | 0.0035 | 0.9557 | 0.0056 | 0.0000 | 0.6351 | 0.6872 | 0.0610 | |
| STG | 0.3282 | 0.2435 | 0.1406 | 0.2283 | 0.1304 | 0.0606 | 0.0624 | 0.1525 | 0.0261 | 0.8168 | 0.0364 | 0.3419 | 0.8886 | 0.9786 | 0.3171 | |
| SVI | 0.3711 | 0.2594 | 0.2869 | 0.2731 | 0.1338 | 0.0535 | 0.0299 | 0.2338 | 0.0099 | 0.9319 | 0.0240 | 0.2886 | 0.9651 | 0.9297 | 0.0000 | |
| TCL | 0.4107 | 0.3301 | 0.1780 | 0.3231 | 0.1255 | 0.0513 | 0.0494 | 0.9294 | 0.1999 | 0.8776 | 0.0389 | 0.3401 | 0.8860 | 0.9638 | 0.2683 | |
| TDP | 0.3309 | 0.1610 | 0.1178 | 0.4012 | 0.1210 | 0.0164 | 0.0115 | 0.0295 | 0.0022 | 0.7643 | 0.0569 | 0.2367 | 0.9568 | 0.7005 | 0.1220 | |
| THI | 0.2569 | 0.1391 | 0.1070 | 0.1190 | 0.1185 | 0.0708 | 0.0375 | 0.0288 | 0.0000 | 0.9120 | 0.0394 | 0.2536 | 0.8581 | 0.9550 | 0.4512 | |
| TMS | 0.3725 | 0.2871 | 0.2244 | 0.0863 | 0.1542 | 0.0597 | 0.0597 | 0.1213 | 0.3556 | 0.8351 | 0.0368 | 0.3045 | 0.8929 | 0.9810 | 0.4756 | |
| TV2 | 0.2625 | 0.1365 | 0.1045 | 0.0000 | 0.1127 | 0.0279 | 0.0417 | 0.0998 | 0.0063 | 1.0000 | 0.0000 | 0.2859 | 0.7011 | 0.8731 | 0.0122 | |
| VIP | 0.4012 | 0.3633 | 0.1586 | 0.3823 | 0.6462 | 0.2805 | 0.0767 | 0.2231 | 0.0115 | 0.4698 | 0.0450 | 0.3493 | 0.8684 | 0.9931 | 0.2561 | |
| VOS | 0.5458 | 0.3795 | 0.1639 | 0.5544 | 0.1142 | 0.0540 | 0.0358 | 0.0981 | 0.0191 | 0.9378 | 0.0617 | 0.5860 | 0.9046 | 0.9580 | 0.5366 | |
| VTB | 0.3100 | 0.2242 | 0.1191 | 0.1633 | 0.2130 | 0.1748 | 0.0607 | 1.0000 | 0.0034 | 0.0000 | 0.0514 | 0.5755 | 0.8471 | 0.9872 | 0.4756 | |
| VTO | 0.2831 | 0.1780 | 0.1026 | 0.3665 | 0.1103 | 0.1013 | 0.1013 | 0.0504 | 0.0133 | 0.6364 | 0.0910 | 0.3327 | 0.8186 | 0.9795 | 0.6341 | |
| ARM | 0.2847 | 0.1447 | 0.1041 | 0.2399 | 0.1357 | 0.0279 | 0.0434 | 0.0679 | 0.0162 | 0.7809 | 0.0250 | 0.3109 | 0.6991 | 0.7483 | 0.0122 | |
| BBS | 0.2891 | 0.1468 | 0.1148 | 0.2044 | 0.1234 | 0.0142 | 0.0245 | 0.0305 | 0.0084 | 0.9087 | 0.0090 | 0.1982 | 0.9443 | 0.7677 | 0.0000 | |
| CDN | 0.3759 | 0.3227 | 0.1428 | 0.3091 | 0.1269 | 0.1407 | 0.0470 | 0.3012 | 0.0462 | 0.8176 | 0.0505 | 0.7347 | 0.7019 | 0.9878 | 0.6098 | |
| CIA | 0.4155 | 0.4031 | 0.1603 | 0.9096 | 0.0829 | 1.0000 | 1.0000 | 0.1724 | 0.0192 | 0.8659 | 0.0429 | 0.0819 | 0.5527 | 1.0000 | 0.1341 | |
| CTB | 0.3966 | 0.2065 | 0.1668 | 0.5431 | 0.1313 | 0.0186 | 0.0165 | 0.0580 | 0.0021 | 0.9310 | 0.0195 | 0.2861 | 0.9036 | 0.7792 | 0.1829 | |
| CTT | 0.3652 | 0.1413 | 0.1231 | 0.3022 | 0.1254 | 0.0088 | 0.0207 | 0.0317 | 0.0248 | 0.8952 | 0.0356 | 0.1319 | 0.9270 | 0.0000 | 0.0244 | |
| DL1 | 0.2608 | 0.1425 | 0.0967 | 1.0000 | 0.1126 | 0.0230 | 0.0413 | 0.0279 | 0.0070 | 0.9315 | 0.0833 | 0.8462 | 0.7569 | 0.9538 | 0.7561 | |
| IPA | 0.2514 | 0.1318 | 0.0970 | 0.3071 | 0.0990 | 0.1447 | 0.1611 | 0.0251 | 0.0160 | 0.6141 | 0.0484 | 1.0000 | 0.4083 | 0.9795 | 1.0000 | |
| PBP | 0.3172 | 0.1978 | 0.1182 | 0.2960 | 0.1290 | 0.0310 | 0.0000 | 0.1746 | 0.0105 | 0.7529 | 1.0000 | 0.1632 | 0.9286 | 0.8785 | 0.0000 | |
| PMP | 0.3184 | 0.1581 | 0.1191 | 0.2328 | 0.1311 | 0.0159 | 0.0172 | 0.0331 | 0.0060 | 0.7269 | 0.0426 | 0.2224 | 0.8855 | 0.7290 | 0.0244 | |
| PMS | 0.3504 | 0.2229 | 0.1604 | 0.5603 | 0.1140 | 0.0252 | 0.0248 | 0.0904 | 0.0251 | 0.6430 | 0.0957 | 0.1354 | 0.9620 | 0.8929 | 0.0000 | |
| PRC | 1.0000 | 1.0000 | 1.0000 | 0.3635 | 1.0000 | 0.1425 | 0.1180 | 0.3053 | 0.0342 | 0.5790 | 0.0319 | 0.1514 | 0.8559 | 0.9738 | 0.3049 | |
| PSC | 0.2693 | 0.1482 | 0.1038 | 0.5916 | 0.1086 | 0.0000 | 0.0153 | 0.0426 | 0.1751 | 0.5728 | 0.1853 | 0.1909 | 0.9480 | 0.9374 | 0.5366 | |
| PTS | 0.3066 | 0.1664 | 0.1185 | 0.3757 | 0.1157 | 0.0000 | 0.0140 | 0.0342 | 0.0339 | 0.8428 | 0.1571 | 0.2402 | 0.8648 | 0.9074 | 0.5610 | |
| SDC | 0.2571 | 0.1440 | 0.0966 | 0.2529 | 0.1188 | 0.0659 | 0.0509 | 0.0840 | 0.0004 | 0.9050 | 0.0006 | 0.4620 | 0.4142 | 0.9365 | 0.1341 | |
| SDG | 0.2886 | 0.1685 | 0.1574 | 0.3045 | 0.1449 | 0.0252 | 0.0228 | 0.0464 | 0.0100 | 0.7599 | 0.0330 | 0.2317 | 0.9440 | 0.8994 | 0.0610 | |
| TPP | 0.2601 | 0.1292 | 0.0945 | 0.4868 | 0.1314 | 0.0168 | 0.0151 | 0.0273 | 0.0045 | 0.9006 | 0.0334 | 0.3443 | 0.9439 | 0.7270 | 0.1707 | |
| VBC | 0.3836 | 0.2051 | 0.1512 | 0.2512 | 0.1267 | 0.0239 | 0.0269 | 0.0468 | 0.0090 | 0.8887 | 0.0204 | 0.1991 | 0.9783 | 0.7778 | 0.0000 | |
| VCM | 0.2335 | 0.1192 | 0.0890 | 0.1090 | 0.1078 | 0.0792 | 0.0278 | 0.1400 | 0.0561 | 0.9390 | 0.0136 | 0.3996 | 0.0000 | 0.9656 | 0.1707 | |
| VSM | 0.4088 | 0.2928 | 0.1855 | 0.3681 | 0.1240 | 0.0420 | 0.0594 | 0.1928 | 0.3300 | 0.8579 | 0.0343 | 0.2011 | 0.9576 | 0.9347 | 0.2561 | |
| **Average** | **0.3442** | **0.2280** | **0.1657** | **0.3511** | **0.1539** | **0.0729** | **0.0613** | **0.1321** | **0.0648** | **0.8041** | **0.0789** | **0.3564** | **0.8347** | **0.8808** | **0.3247** | |

**Table S22. Positive distance from average for all indicators in 2022 based on EDAS**

| Company | Indicator | | | | | | | | | | | | | | |  |
| --- | --- | --- | --- | --- | --- | --- | --- | --- | --- | --- | --- | --- | --- | --- | --- | --- |
|  | I1 | I2 | I3 | I4 | I5 | I6 | I7 | I8 | I9 | I10 | I11 | I12 | I13 | I14 | I15 | |
| CAV | 0.1742 | 0.0000 | 0.4116 | 0.0000 | 0.0000 | 0.0000 | 0.0000 | 0.0000 | 0.0000 | 0.0000 | 0.0000 | 0.3924 | 0.0000 | 0.0383 | 0.6619 | |
| GEX | 0.0000 | 0.0000 | 0.0000 | 0.0000 | 0.0000 | 0.0000 | 0.0000 | 0.0000 | 0.0000 | 0.0000 | 0.0000 | 0.0000 | 0.0000 | 0.0000 | 0.0000 | |
| GMD | 0.0000 | 0.0458 | 0.0000 | 0.0065 | 0.0000 | 0.0000 | 0.0000 | 0.0000 | 0.0000 | 0.0000 | 0.0000 | 0.0000 | 0.2364 | 0.0000 | 0.0000 | |
| GSP | 0.0000 | 0.0000 | 0.0000 | 0.0000 | 0.0000 | 0.0000 | 0.0000 | 0.0000 | 0.5396 | 0.0000 | 0.0000 | 0.4602 | 0.0000 | 0.0000 | 0.0000 | |
| HAH | 0.4874 | 0.7842 | 1.0765 | 0.5072 | 0.0000 | 0.0000 | 0.0000 | 0.4127 | 0.0000 | 0.0479 | 0.0000 | 0.0000 | 0.0000 | 0.0000 | 0.0000 | |
| L10 | 0.0000 | 0.0000 | 0.0000 | 0.0000 | 0.0000 | 0.0000 | 0.0000 | 0.0000 | 0.0000 | 0.0000 | 0.0000 | 0.7314 | 0.0000 | 0.1353 | 0.0000 | |
| MSN | 0.0000 | 0.0000 | 0.0000 | 0.0000 | 0.0000 | 0.0000 | 0.0000 | 0.0000 | 0.0000 | 0.0000 | 1.0426 | 0.0000 | 0.0000 | 0.0854 | 0.0000 | |
| PAC | 0.0900 | 0.0000 | 0.0000 | 0.0000 | 0.0000 | 0.0000 | 0.0000 | 0.0000 | 0.0000 | 0.2151 | 1.7528 | 0.1897 | 0.0000 | 0.0577 | 0.9249 | |
| PDN | 0.3881 | 0.7732 | 1.0754 | 0.0000 | 0.0000 | 0.0000 | 0.0000 | 0.3118 | 14.4250 | 0.0000 | 0.0000 | 0.0000 | 0.0000 | 0.0000 | 0.0000 | |
| PJT | 0.0000 | 0.0000 | 0.0000 | 0.1183 | 0.0000 | 0.0000 | 0.0000 | 0.0000 | 0.0000 | 0.2393 | 2.4529 | 0.5192 | 0.0000 | 0.0000 | 0.0000 | |
| PVP | 0.0000 | 0.0786 | 0.0000 | 0.0000 | 0.0000 | 0.0918 | 0.3736 | 0.1467 | 1.2579 | 0.0000 | 0.0000 | 0.5687 | 0.0000 | 0.0000 | 0.0000 | |
| PVT | 0.0000 | 0.0000 | 0.0000 | 0.0029 | 0.0000 | 0.0000 | 0.0000 | 0.0000 | 0.0150 | 0.0000 | 0.0000 | 0.0000 | 0.0000 | 0.0000 | 0.0000 | |
| REE | 0.0308 | 0.0342 | 0.5287 | 0.4756 | 0.0000 | 0.0000 | 0.0000 | 0.0000 | 0.0000 | 0.0000 | 0.0000 | 0.0000 | 0.0000 | 0.0000 | 0.0000 | |
| SRF | 0.0000 | 0.0000 | 0.0000 | 0.0756 | 0.0000 | 0.0000 | 0.0000 | 0.0000 | 0.0000 | 0.0000 | 0.0000 | 1.0000 | 0.2391 | 0.2198 | 0.8122 | |
| STG | 0.0000 | 0.0683 | 0.0000 | 0.0000 | 0.0000 | 0.0000 | 0.0182 | 0.1543 | 0.0000 | 0.0000 | 0.0000 | 0.0408 | 0.0000 | 0.0000 | 0.0234 | |
| SVI | 0.0781 | 0.1378 | 0.7318 | 0.0000 | 0.0000 | 0.0000 | 0.0000 | 0.7700 | 0.0000 | 0.0000 | 0.0000 | 0.1902 | 0.0000 | 0.0000 | 1.0000 | |
| TCL | 0.1930 | 0.4478 | 0.0746 | 0.0000 | 0.0000 | 0.0000 | 0.0000 | 6.0352 | 2.0834 | 0.0000 | 0.0000 | 0.0457 | 0.0000 | 0.0000 | 0.1736 | |
| TDP | 0.0000 | 0.0000 | 0.0000 | 0.1426 | 0.0000 | 0.0000 | 0.0000 | 0.0000 | 0.0000 | 0.0495 | 0.0000 | 0.3360 | 0.0000 | 0.2048 | 0.6244 | |
| THI | 0.0000 | 0.0000 | 0.0000 | 0.0000 | 0.0000 | 0.0000 | 0.0000 | 0.0000 | 0.0000 | 0.0000 | 0.0000 | 0.2886 | 0.0000 | 0.0000 | 0.0000 | |
| TMS | 0.0822 | 0.2593 | 0.3545 | 0.0000 | 0.0021 | 0.0000 | 0.0000 | 0.0000 | 4.4855 | 0.0000 | 0.0000 | 0.1458 | 0.0000 | 0.0000 | 0.0000 | |
| TV2 | 0.0000 | 0.0000 | 0.0000 | 0.0000 | 0.0000 | 0.0000 | 0.0000 | 0.0000 | 0.0000 | 0.0000 | 0.0000 | 0.1979 | 0.1601 | 0.0088 | 0.9624 | |
| VIP | 0.1654 | 0.5938 | 0.0000 | 0.0888 | 3.1989 | 2.8455 | 0.2515 | 0.6888 | 0.0000 | 0.4158 | 0.0000 | 0.0201 | 0.0000 | 0.0000 | 0.2112 | |
| VOS | 0.5856 | 0.6645 | 0.0000 | 0.5787 | 0.0000 | 0.0000 | 0.0000 | 0.0000 | 0.0000 | 0.0000 | 0.0000 | 0.0000 | 0.0000 | 0.0000 | 0.0000 | |
| VTB | 0.0000 | 0.0000 | 0.0000 | 0.0000 | 0.3839 | 1.3959 | 0.0000 | 6.5700 | 0.0000 | 1.0000 | 0.0000 | 0.0000 | 0.0000 | 0.0000 | 0.0000 | |
| VTO | 0.0000 | 0.0000 | 0.0000 | 0.0437 | 0.0000 | 0.3890 | 0.6525 | 0.0000 | 0.0000 | 0.2085 | 0.1531 | 0.0667 | 0.0193 | 0.0000 | 0.0000 | |
| ARM | 0.0000 | 0.0000 | 0.0000 | 0.0000 | 0.0000 | 0.0000 | 0.0000 | 0.0000 | 0.0000 | 0.0289 | 0.0000 | 0.1278 | 0.1625 | 0.1505 | 0.9624 | |
| BBS | 0.0000 | 0.0000 | 0.0000 | 0.0000 | 0.0000 | 0.0000 | 0.0000 | 0.0000 | 0.0000 | 0.0000 | 0.0000 | 0.4440 | 0.0000 | 0.1285 | 1.0000 | |
| CDN | 0.0919 | 0.4156 | 0.0000 | 0.0000 | 0.0000 | 0.9288 | 0.0000 | 1.2802 | 0.0000 | 0.0000 | 0.0000 | 0.0000 | 0.1592 | 0.0000 | 0.0000 | |
| CIA | 0.2071 | 0.7681 | 0.0000 | 1.5903 | 0.0000 | 12.7080 | 15.3135 | 0.3053 | 0.0000 | 0.0000 | 0.0000 | 0.7702 | 0.3379 | 0.0000 | 0.5868 | |
| CTB | 0.1521 | 0.0000 | 0.0066 | 0.5467 | 0.0000 | 0.0000 | 0.0000 | 0.0000 | 0.0000 | 0.0000 | 0.0000 | 0.1975 | 0.0000 | 0.1154 | 0.4366 | |
| CTT | 0.0610 | 0.0000 | 0.0000 | 0.0000 | 0.0000 | 0.0000 | 0.0000 | 0.0000 | 0.0000 | 0.0000 | 0.0000 | 0.6300 | 0.0000 | 1.0000 | 0.9249 | |
| DL1 | 0.0000 | 0.0000 | 0.0000 | 1.8478 | 0.0000 | 0.0000 | 0.0000 | 0.0000 | 0.0000 | 0.0000 | 0.0552 | 0.0000 | 0.0932 | 0.0000 | 0.0000 | |
| IPA | 0.0000 | 0.0000 | 0.0000 | 0.0000 | 0.0000 | 0.9834 | 1.6285 | 0.0000 | 0.0000 | 0.2363 | 0.0000 | 0.0000 | 0.5108 | 0.0000 | 0.0000 | |
| PBP | 0.0000 | 0.0000 | 0.0000 | 0.0000 | 0.0000 | 0.0000 | 0.0000 | 0.3214 | 0.0000 | 0.0636 | 11.6692 | 0.5420 | 0.0000 | 0.0026 | 1.0000 | |
| PMP | 0.0000 | 0.0000 | 0.0000 | 0.0000 | 0.0000 | 0.0000 | 0.0000 | 0.0000 | 0.0000 | 0.0960 | 0.0000 | 0.3761 | 0.0000 | 0.1724 | 0.9249 | |
| PMS | 0.0179 | 0.0000 | 0.0000 | 0.5956 | 0.0000 | 0.0000 | 0.0000 | 0.0000 | 0.0000 | 0.2003 | 0.2124 | 0.6202 | 0.0000 | 0.0000 | 1.0000 | |
| PRC | 1.9050 | 3.3864 | 5.0356 | 0.0352 | 5.4980 | 0.9531 | 0.9250 | 1.3114 | 0.0000 | 0.2799 | 0.0000 | 0.5753 | 0.0000 | 0.0000 | 0.0609 | |
| PSC | 0.0000 | 0.0000 | 0.0000 | 0.6849 | 0.0000 | 0.0000 | 0.0000 | 0.0000 | 1.7016 | 0.2877 | 1.3477 | 0.4645 | 0.0000 | 0.0000 | 0.0000 | |
| PTS | 0.0000 | 0.0000 | 0.0000 | 0.0700 | 0.0000 | 0.0000 | 0.0000 | 0.0000 | 0.0000 | 0.0000 | 0.9904 | 0.3261 | 0.0000 | 0.0000 | 0.0000 | |
| SDC | 0.0000 | 0.0000 | 0.0000 | 0.0000 | 0.0000 | 0.0000 | 0.0000 | 0.0000 | 0.0000 | 0.0000 | 0.0000 | 0.0000 | 0.5038 | 0.0000 | 0.5868 | |
| SDG | 0.0000 | 0.0000 | 0.0000 | 0.0000 | 0.0000 | 0.0000 | 0.0000 | 0.0000 | 0.0000 | 0.0549 | 0.0000 | 0.3499 | 0.0000 | 0.0000 | 0.8122 | |
| TPP | 0.0000 | 0.0000 | 0.0000 | 0.3863 | 0.0000 | 0.0000 | 0.0000 | 0.0000 | 0.0000 | 0.0000 | 0.0000 | 0.0340 | 0.0000 | 0.1746 | 0.4741 | |
| VBC | 0.1143 | 0.0000 | 0.0000 | 0.0000 | 0.0000 | 0.0000 | 0.0000 | 0.0000 | 0.0000 | 0.0000 | 0.0000 | 0.4413 | 0.0000 | 0.1169 | 1.0000 | |
| VCM | 0.0000 | 0.0000 | 0.0000 | 0.0000 | 0.0000 | 0.0857 | 0.0000 | 0.0596 | 0.0000 | 0.0000 | 0.0000 | 0.0000 | 1.0000 | 0.0000 | 0.4741 | |
| VSM | 0.1876 | 0.2844 | 0.1199 | 0.0484 | 0.0000 | 0.0000 | 0.0000 | 0.4597 | 4.0897 | 0.0000 | 0.0000 | 0.4358 | 0.0000 | 0.0000 | 0.2112 | |

**Table S23. Negative distance from average for all indicators in 2022 based on EDAS**

| Company | Indicator | | | | | | | | | | | | | | |  |
| --- | --- | --- | --- | --- | --- | --- | --- | --- | --- | --- | --- | --- | --- | --- | --- | --- |
|  | I1 | I2 | I3 | I4 | I5 | I6 | I7 | I8 | I9 | I10 | I11 | I12 | I13 | I14 | I15 | |
| CAV | 0.0000 | 0.0571 | 0.0000 | 0.1514 | 0.1659 | 0.6361 | 0.8907 | 0.5920 | 0.9641 | 0.0453 | 0.0448 | 0.0000 | 0.1980 | 0.0000 | 0.0000 | |
| GEX | 0.2871 | 0.4507 | 0.4162 | 0.1048 | 0.2122 | 0.7149 | 0.7349 | 0.7348 | 0.9801 | 0.1149 | 0.3499 | 0.1132 | 0.0545 | 0.0361 | 0.6528 | |
| GMD | 0.0140 | 0.0000 | 0.0358 | 0.0000 | 0.1137 | 0.9151 | 0.6238 | 0.2654 | 0.3777 | 0.1870 | 0.4749 | 1.3411 | 0.0000 | 0.0888 | 0.3523 | |
| GSP | 0.0520 | 0.1182 | 0.2898 | 0.0941 | 0.1223 | 0.4784 | 0.6280 | 0.6069 | 0.0000 | 0.0322 | 0.2005 | 0.0000 | 0.1377 | 0.0832 | 1.1035 | |
| HAH | 0.0000 | 0.0000 | 0.0000 | 0.0000 | 0.1473 | 0.2479 | 0.0493 | 0.0000 | 0.3940 | 0.0000 | 0.0741 | 1.3554 | 0.1126 | 0.1000 | 1.1411 | |
| L10 | 0.1713 | 0.3986 | 0.2846 | 0.2883 | 0.1403 | 0.4662 | 0.2180 | 0.6600 | 0.9347 | 0.1521 | 0.8114 | 0.0000 | 0.1712 | 0.0000 | 0.0893 | |
| MSN | 0.1146 | 0.3208 | 0.1409 | 0.4251 | 0.2640 | 0.9697 | 0.7871 | 0.7605 | 0.9447 | 0.1185 | 0.0000 | 0.4969 | 0.0594 | 0.0000 | 0.3898 | |
| PAC | 0.0000 | 0.0777 | 0.0637 | 0.1464 | 0.2090 | 0.7149 | 0.9903 | 0.6099 | 0.9436 | 0.0000 | 0.0000 | 0.0000 | 0.1729 | 0.0000 | 0.0000 | |
| PDN | 0.0000 | 0.0000 | 0.0000 | 0.0168 | 0.1347 | 0.4420 | 0.3086 | 0.0000 | 0.0000 | 0.1165 | 0.3006 | 0.8355 | 0.0548 | 0.0951 | 0.0518 | |
| PJT | 0.1486 | 0.2044 | 0.3613 | 0.0000 | 0.2172 | 0.8120 | 0.8164 | 0.7076 | 0.6801 | 0.0000 | 0.0000 | 0.0000 | 0.1051 | 0.0949 | 0.5776 | |
| PVP | 0.0071 | 0.0000 | 0.1692 | 0.0154 | 0.1897 | 0.0000 | 0.0000 | 0.0000 | 0.0000 | 0.1622 | 0.8142 | 0.0000 | 0.1645 | 0.0956 | 0.3523 | |
| PVT | 0.0570 | 0.0803 | 0.1325 | 0.0000 | 0.1494 | 0.1630 | 0.3944 | 0.4433 | 0.0000 | 0.0639 | 0.4499 | 0.0192 | 0.0789 | 0.0916 | 0.8781 | |
| REE | 0.0000 | 0.0000 | 0.0000 | 0.0000 | 0.1730 | 0.1387 | 0.1012 | 0.5761 | 0.9331 | 0.1071 | 0.7592 | 1.4502 | 0.0205 | 0.1129 | 1.6669 | |
| SRF | 1.0000 | 1.0000 | 1.0000 | 0.0000 | 1.0000 | 0.7271 | 0.5672 | 1.0000 | 0.9463 | 0.1886 | 0.9286 | 0.0000 | 0.0000 | 0.0000 | 0.0000 | |
| STG | 0.0466 | 0.0000 | 0.1513 | 0.3498 | 0.1529 | 0.1690 | 0.0000 | 0.0000 | 0.5974 | 0.0158 | 0.5385 | 0.0000 | 0.0645 | 0.1110 | 0.0000 | |
| SVI | 0.0000 | 0.0000 | 0.0000 | 0.2222 | 0.1308 | 0.2661 | 0.5126 | 0.0000 | 0.8468 | 0.1590 | 0.6964 | 0.0000 | 0.1561 | 0.0555 | 0.0000 | |
| TCL | 0.0000 | 0.0000 | 0.0000 | 0.0798 | 0.1843 | 0.2964 | 0.1946 | 0.0000 | 0.0000 | 0.0914 | 0.5070 | 0.0000 | 0.0615 | 0.0942 | 0.0000 | |
| TDP | 0.0387 | 0.2938 | 0.2888 | 0.0000 | 0.2138 | 0.7756 | 0.8121 | 0.7766 | 0.9660 | 0.0000 | 0.2791 | 0.0000 | 0.1462 | 0.0000 | 0.0000 | |
| THI | 0.2536 | 0.3896 | 0.3541 | 0.6612 | 0.2298 | 0.0295 | 0.3879 | 0.7820 | 1.0000 | 0.1342 | 0.5006 | 0.0000 | 0.0279 | 0.0842 | 0.3898 | |
| TMS | 0.0000 | 0.0000 | 0.0000 | 0.7542 | 0.0000 | 0.1812 | 0.0257 | 0.0815 | 0.0000 | 0.0386 | 0.5335 | 0.0000 | 0.0697 | 0.1137 | 0.4649 | |
| TV2 | 0.2374 | 0.4012 | 0.3693 | 1.0000 | 0.2677 | 0.6179 | 0.3196 | 0.2442 | 0.9023 | 0.2436 | 1.0000 | 0.0000 | 0.0000 | 0.0000 | 0.0000 | |
| VIP | 0.0000 | 0.0000 | 0.0427 | 0.0000 | 0.0000 | 0.0000 | 0.0000 | 0.0000 | 0.8226 | 0.0000 | 0.4299 | 0.0000 | 0.0403 | 0.1274 | 0.0000 | |
| VOS | 0.0000 | 0.0000 | 0.0109 | 0.0000 | 0.2578 | 0.2600 | 0.4162 | 0.2572 | 0.7052 | 0.1662 | 0.2184 | 0.6441 | 0.0837 | 0.0876 | 0.6528 | |
| VTB | 0.0994 | 0.0166 | 0.2811 | 0.5351 | 0.0000 | 0.0000 | 0.0096 | 0.0000 | 0.9482 | 0.0000 | 0.3491 | 0.6145 | 0.0148 | 0.1208 | 0.4649 | |
| VTO | 0.1777 | 0.2192 | 0.3806 | 0.0000 | 0.2832 | 0.0000 | 0.0000 | 0.6185 | 0.7954 | 0.0000 | 0.0000 | 0.0000 | 0.0000 | 0.1120 | 0.9533 | |
| ARM | 0.1729 | 0.3652 | 0.3720 | 0.3169 | 0.1179 | 0.6179 | 0.2917 | 0.4861 | 0.7497 | 0.0000 | 0.6835 | 0.0000 | 0.0000 | 0.0000 | 0.0000 | |
| BBS | 0.1601 | 0.3562 | 0.3071 | 0.4178 | 0.1979 | 0.8059 | 0.6000 | 0.7692 | 0.8708 | 0.1301 | 0.8857 | 0.0000 | 0.1312 | 0.0000 | 0.0000 | |
| CDN | 0.0000 | 0.0000 | 0.1382 | 0.1199 | 0.1752 | 0.0000 | 0.2337 | 0.0000 | 0.2880 | 0.0167 | 0.3599 | 1.0612 | 0.0000 | 0.1214 | 0.8781 | |
| CIA | 0.0000 | 0.0000 | 0.0323 | 0.0000 | 0.4616 | 0.0000 | 0.0000 | 0.0000 | 0.7043 | 0.0769 | 0.4570 | 0.0000 | 0.0000 | 0.1353 | 0.0000 | |
| CTB | 0.0000 | 0.0944 | 0.0000 | 0.0000 | 0.1466 | 0.7452 | 0.7311 | 0.5607 | 0.9671 | 0.1577 | 0.7528 | 0.0000 | 0.0825 | 0.0000 | 0.0000 | |
| CTT | 0.0000 | 0.3800 | 0.2567 | 0.1394 | 0.1854 | 0.8787 | 0.6628 | 0.7602 | 0.6168 | 0.1133 | 0.5485 | 0.0000 | 0.1105 | 0.0000 | 0.0000 | |
| DL1 | 0.2424 | 0.3748 | 0.4166 | 0.0000 | 0.2684 | 0.6846 | 0.3269 | 0.7884 | 0.8922 | 0.1585 | 0.0000 | 1.3740 | 0.0000 | 0.0828 | 1.3289 | |
| IPA | 0.2698 | 0.4218 | 0.4144 | 0.1254 | 0.3570 | 0.0000 | 0.0000 | 0.8099 | 0.7525 | 0.0000 | 0.3863 | 1.8056 | 0.0000 | 0.1120 | 2.0801 | |
| PBP | 0.0785 | 0.1324 | 0.2866 | 0.1570 | 0.1614 | 0.5754 | 1.0000 | 0.0000 | 0.8374 | 0.0000 | 0.0000 | 0.0000 | 0.1124 | 0.0000 | 0.0000 | |
| PMP | 0.0751 | 0.3067 | 0.2815 | 0.3370 | 0.1481 | 0.7816 | 0.7202 | 0.7497 | 0.9073 | 0.0000 | 0.4606 | 0.0000 | 0.0608 | 0.0000 | 0.0000 | |
| PMS | 0.0000 | 0.0224 | 0.0317 | 0.0000 | 0.2589 | 0.6543 | 0.5962 | 0.3155 | 0.6123 | 0.0000 | 0.0000 | 0.0000 | 0.1524 | 0.0136 | 0.0000 | |
| PRC | 0.0000 | 0.0000 | 0.0000 | 0.0000 | 0.0000 | 0.0000 | 0.0000 | 0.0000 | 0.4727 | 0.0000 | 0.5963 | 0.0000 | 0.0254 | 0.1056 | 0.0000 | |
| PSC | 0.2177 | 0.3498 | 0.3734 | 0.0000 | 0.2940 | 1.0000 | 0.7510 | 0.6774 | 0.0000 | 0.0000 | 0.0000 | 0.0000 | 0.1357 | 0.0643 | 0.6528 | |
| PTS | 0.1094 | 0.2700 | 0.2848 | 0.0000 | 0.2482 | 1.0000 | 0.7718 | 0.7412 | 0.4778 | 0.0482 | 0.0000 | 0.0000 | 0.0360 | 0.0301 | 0.7279 | |
| SDC | 0.2531 | 0.3684 | 0.4169 | 0.2799 | 0.2283 | 0.0962 | 0.1694 | 0.3645 | 0.9934 | 0.1255 | 0.9929 | 0.2960 | 0.0000 | 0.0632 | 0.0000 | |
| SDG | 0.1615 | 0.2610 | 0.0498 | 0.1329 | 0.0585 | 0.6543 | 0.6275 | 0.6485 | 0.8456 | 0.0000 | 0.5820 | 0.0000 | 0.1309 | 0.0211 | 0.0000 | |
| TPP | 0.2445 | 0.4334 | 0.4298 | 0.0000 | 0.1459 | 0.7695 | 0.7533 | 0.7934 | 0.9313 | 0.1200 | 0.5763 | 0.0000 | 0.1308 | 0.0000 | 0.0000 | |
| VBC | 0.0000 | 0.1002 | 0.0875 | 0.2848 | 0.1770 | 0.6725 | 0.5615 | 0.6459 | 0.8614 | 0.1053 | 0.7414 | 0.0000 | 0.1720 | 0.0000 | 0.0000 | |
| VCM | 0.3216 | 0.4771 | 0.4631 | 0.6896 | 0.2993 | 0.0000 | 0.5461 | 0.0000 | 0.1350 | 0.1677 | 0.8271 | 0.1210 | 0.0000 | 0.0963 | 0.0000 | |
| VSM | 0.0000 | 0.0000 | 0.0000 | 0.0000 | 0.1943 | 0.4238 | 0.0316 | 0.0000 | 0.0000 | 0.0668 | 0.5656 | 0.0000 | 0.1472 | 0.0611 | 0.0000 | |

**Table S24. Weighted positive distance for all indicators in 2022 based on EDAS.**

| Company | Indicator | | | | | | | | | | | | | | |  |
| --- | --- | --- | --- | --- | --- | --- | --- | --- | --- | --- | --- | --- | --- | --- | --- | --- |
|  | I1 | I2 | I3 | I4 | I5 | I6 | I7 | I8 | I9 | I10 | I11 | I12 | I13 | I14 | I15 | |
| CAV | 0.0071 | 0.0000 | 0.0190 | 0.0000 | 0.0000 | 0.0000 | 0.0000 | 0.0000 | 0.0000 | 0.0000 | 0.0000 | 0.0379 | 0.0000 | 0.0022 | 0.0688 | |
| GEX | 0.0000 | 0.0000 | 0.0000 | 0.0000 | 0.0000 | 0.0000 | 0.0000 | 0.0000 | 0.0000 | 0.0000 | 0.0000 | 0.0000 | 0.0000 | 0.0000 | 0.0000 | |
| GMD | 0.0000 | 0.0020 | 0.0000 | 0.0004 | 0.0000 | 0.0000 | 0.0000 | 0.0000 | 0.0000 | 0.0000 | 0.0000 | 0.0000 | 0.0195 | 0.0000 | 0.0000 | |
| GSP | 0.0000 | 0.0000 | 0.0000 | 0.0000 | 0.0000 | 0.0000 | 0.0000 | 0.0000 | 0.0345 | 0.0000 | 0.0000 | 0.0444 | 0.0000 | 0.0000 | 0.0000 | |
| HAH | 0.0200 | 0.0341 | 0.0496 | 0.0350 | 0.0000 | 0.0000 | 0.0000 | 0.0316 | 0.0000 | 0.0041 | 0.0000 | 0.0000 | 0.0000 | 0.0000 | 0.0000 | |
| L10 | 0.0000 | 0.0000 | 0.0000 | 0.0000 | 0.0000 | 0.0000 | 0.0000 | 0.0000 | 0.0000 | 0.0000 | 0.0000 | 0.0706 | 0.0000 | 0.0079 | 0.0000 | |
| MSN | 0.0000 | 0.0000 | 0.0000 | 0.0000 | 0.0000 | 0.0000 | 0.0000 | 0.0000 | 0.0000 | 0.0000 | 0.0703 | 0.0000 | 0.0000 | 0.0050 | 0.0000 | |
| PAC | 0.0037 | 0.0000 | 0.0000 | 0.0000 | 0.0000 | 0.0000 | 0.0000 | 0.0000 | 0.0000 | 0.0184 | 0.1182 | 0.0183 | 0.0000 | 0.0034 | 0.0961 | |
| PDN | 0.0159 | 0.0336 | 0.0496 | 0.0000 | 0.0000 | 0.0000 | 0.0000 | 0.0239 | 0.9235 | 0.0000 | 0.0000 | 0.0000 | 0.0000 | 0.0000 | 0.0000 | |
| PJT | 0.0000 | 0.0000 | 0.0000 | 0.0082 | 0.0000 | 0.0000 | 0.0000 | 0.0000 | 0.0000 | 0.0205 | 0.1654 | 0.0501 | 0.0000 | 0.0000 | 0.0000 | |
| PVP | 0.0000 | 0.0034 | 0.0000 | 0.0000 | 0.0000 | 0.0051 | 0.0206 | 0.0112 | 0.0805 | 0.0000 | 0.0000 | 0.0549 | 0.0000 | 0.0000 | 0.0000 | |
| PVT | 0.0000 | 0.0000 | 0.0000 | 0.0002 | 0.0000 | 0.0000 | 0.0000 | 0.0000 | 0.0010 | 0.0000 | 0.0000 | 0.0000 | 0.0000 | 0.0000 | 0.0000 | |
| REE | 0.0013 | 0.0015 | 0.0244 | 0.0328 | 0.0000 | 0.0000 | 0.0000 | 0.0000 | 0.0000 | 0.0000 | 0.0000 | 0.0000 | 0.0000 | 0.0000 | 0.0000 | |
| SRF | 0.0000 | 0.0000 | 0.0000 | 0.0052 | 0.0000 | 0.0000 | 0.0000 | 0.0000 | 0.0000 | 0.0000 | 0.0000 | 0.0965 | 0.0197 | 0.0128 | 0.0844 | |
| STG | 0.0000 | 0.0030 | 0.0000 | 0.0000 | 0.0000 | 0.0000 | 0.0010 | 0.0118 | 0.0000 | 0.0000 | 0.0000 | 0.0039 | 0.0000 | 0.0000 | 0.0024 | |
| SVI | 0.0032 | 0.0060 | 0.0337 | 0.0000 | 0.0000 | 0.0000 | 0.0000 | 0.0590 | 0.0000 | 0.0000 | 0.0000 | 0.0184 | 0.0000 | 0.0000 | 0.1040 | |
| TCL | 0.0079 | 0.0195 | 0.0034 | 0.0000 | 0.0000 | 0.0000 | 0.0000 | 0.4624 | 0.1334 | 0.0000 | 0.0000 | 0.0044 | 0.0000 | 0.0000 | 0.0180 | |
| TDP | 0.0000 | 0.0000 | 0.0000 | 0.0098 | 0.0000 | 0.0000 | 0.0000 | 0.0000 | 0.0000 | 0.0042 | 0.0000 | 0.0324 | 0.0000 | 0.0120 | 0.0649 | |
| THI | 0.0000 | 0.0000 | 0.0000 | 0.0000 | 0.0000 | 0.0000 | 0.0000 | 0.0000 | 0.0000 | 0.0000 | 0.0000 | 0.0279 | 0.0000 | 0.0000 | 0.0000 | |
| TMS | 0.0034 | 0.0113 | 0.0163 | 0.0000 | 0.0001 | 0.0000 | 0.0000 | 0.0000 | 0.2872 | 0.0000 | 0.0000 | 0.0141 | 0.0000 | 0.0000 | 0.0000 | |
| TV2 | 0.0000 | 0.0000 | 0.0000 | 0.0000 | 0.0000 | 0.0000 | 0.0000 | 0.0000 | 0.0000 | 0.0000 | 0.0000 | 0.0191 | 0.0132 | 0.0005 | 0.1000 | |
| VIP | 0.0068 | 0.0258 | 0.0000 | 0.0061 | 0.1739 | 0.1592 | 0.0139 | 0.0528 | 0.0000 | 0.0356 | 0.0000 | 0.0019 | 0.0000 | 0.0000 | 0.0220 | |
| VOS | 0.0240 | 0.0289 | 0.0000 | 0.0399 | 0.0000 | 0.0000 | 0.0000 | 0.0000 | 0.0000 | 0.0000 | 0.0000 | 0.0000 | 0.0000 | 0.0000 | 0.0000 | |
| VTB | 0.0000 | 0.0000 | 0.0000 | 0.0000 | 0.0209 | 0.0781 | 0.0000 | 0.5033 | 0.0000 | 0.0857 | 0.0000 | 0.0000 | 0.0000 | 0.0000 | 0.0000 | |
| VTO | 0.0000 | 0.0000 | 0.0000 | 0.0030 | 0.0000 | 0.0218 | 0.0359 | 0.0000 | 0.0000 | 0.0179 | 0.0103 | 0.0064 | 0.0016 | 0.0000 | 0.0000 | |
| ARM | 0.0000 | 0.0000 | 0.0000 | 0.0000 | 0.0000 | 0.0000 | 0.0000 | 0.0000 | 0.0000 | 0.0025 | 0.0000 | 0.0123 | 0.0134 | 0.0088 | 0.1000 | |
| BBS | 0.0000 | 0.0000 | 0.0000 | 0.0000 | 0.0000 | 0.0000 | 0.0000 | 0.0000 | 0.0000 | 0.0000 | 0.0000 | 0.0428 | 0.0000 | 0.0075 | 0.1040 | |
| CDN | 0.0038 | 0.0181 | 0.0000 | 0.0000 | 0.0000 | 0.0520 | 0.0000 | 0.0981 | 0.0000 | 0.0000 | 0.0000 | 0.0000 | 0.0131 | 0.0000 | 0.0000 | |
| CIA | 0.0085 | 0.0334 | 0.0000 | 0.1096 | 0.0000 | 0.7108 | 0.8435 | 0.0234 | 0.0000 | 0.0000 | 0.0000 | 0.0743 | 0.0279 | 0.0000 | 0.0610 | |
| CTB | 0.0062 | 0.0000 | 0.0003 | 0.0377 | 0.0000 | 0.0000 | 0.0000 | 0.0000 | 0.0000 | 0.0000 | 0.0000 | 0.0191 | 0.0000 | 0.0067 | 0.0454 | |
| CTT | 0.0025 | 0.0000 | 0.0000 | 0.0000 | 0.0000 | 0.0000 | 0.0000 | 0.0000 | 0.0000 | 0.0000 | 0.0000 | 0.0608 | 0.0000 | 0.0584 | 0.0961 | |
| DL1 | 0.0000 | 0.0000 | 0.0000 | 0.1274 | 0.0000 | 0.0000 | 0.0000 | 0.0000 | 0.0000 | 0.0000 | 0.0037 | 0.0000 | 0.0077 | 0.0000 | 0.0000 | |
| IPA | 0.0000 | 0.0000 | 0.0000 | 0.0000 | 0.0000 | 0.0550 | 0.0897 | 0.0000 | 0.0000 | 0.0202 | 0.0000 | 0.0000 | 0.0422 | 0.0000 | 0.0000 | |
| PBP | 0.0000 | 0.0000 | 0.0000 | 0.0000 | 0.0000 | 0.0000 | 0.0000 | 0.0246 | 0.0000 | 0.0055 | 0.7868 | 0.0523 | 0.0000 | 0.0002 | 0.1040 | |
| PMP | 0.0000 | 0.0000 | 0.0000 | 0.0000 | 0.0000 | 0.0000 | 0.0000 | 0.0000 | 0.0000 | 0.0082 | 0.0000 | 0.0363 | 0.0000 | 0.0101 | 0.0961 | |
| PMS | 0.0007 | 0.0000 | 0.0000 | 0.0411 | 0.0000 | 0.0000 | 0.0000 | 0.0000 | 0.0000 | 0.0172 | 0.0143 | 0.0599 | 0.0000 | 0.0000 | 0.1040 | |
| PRC | 0.0780 | 0.1471 | 0.2321 | 0.0024 | 0.2989 | 0.0533 | 0.0509 | 0.1005 | 0.0000 | 0.0240 | 0.0000 | 0.0555 | 0.0000 | 0.0000 | 0.0063 | |
| PSC | 0.0000 | 0.0000 | 0.0000 | 0.0472 | 0.0000 | 0.0000 | 0.0000 | 0.0000 | 0.1089 | 0.0246 | 0.0909 | 0.0448 | 0.0000 | 0.0000 | 0.0000 | |
| PTS | 0.0000 | 0.0000 | 0.0000 | 0.0048 | 0.0000 | 0.0000 | 0.0000 | 0.0000 | 0.0000 | 0.0000 | 0.0668 | 0.0315 | 0.0000 | 0.0000 | 0.0000 | |
| SDC | 0.0000 | 0.0000 | 0.0000 | 0.0000 | 0.0000 | 0.0000 | 0.0000 | 0.0000 | 0.0000 | 0.0000 | 0.0000 | 0.0000 | 0.0416 | 0.0000 | 0.0610 | |
| SDG | 0.0000 | 0.0000 | 0.0000 | 0.0000 | 0.0000 | 0.0000 | 0.0000 | 0.0000 | 0.0000 | 0.0047 | 0.0000 | 0.0338 | 0.0000 | 0.0000 | 0.0844 | |
| TPP | 0.0000 | 0.0000 | 0.0000 | 0.0266 | 0.0000 | 0.0000 | 0.0000 | 0.0000 | 0.0000 | 0.0000 | 0.0000 | 0.0033 | 0.0000 | 0.0102 | 0.0493 | |
| VBC | 0.0047 | 0.0000 | 0.0000 | 0.0000 | 0.0000 | 0.0000 | 0.0000 | 0.0000 | 0.0000 | 0.0000 | 0.0000 | 0.0426 | 0.0000 | 0.0068 | 0.1040 | |
| VCM | 0.0000 | 0.0000 | 0.0000 | 0.0000 | 0.0000 | 0.0048 | 0.0000 | 0.0046 | 0.0000 | 0.0000 | 0.0000 | 0.0000 | 0.0826 | 0.0000 | 0.0493 | |
| VSM | 0.0077 | 0.0124 | 0.0055 | 0.0033 | 0.0000 | 0.0000 | 0.0000 | 0.0352 | 0.2618 | 0.0000 | 0.0000 | 0.0421 | 0.0000 | 0.0000 | 0.0220 | |

**Table S25. Weighted negative distance for all indicators in 2022 based on EDAS.**

| Company | Indicator | | | | | | | | | | | | | | |  |
| --- | --- | --- | --- | --- | --- | --- | --- | --- | --- | --- | --- | --- | --- | --- | --- | --- |
|  | I1 | I2 | I3 | I4 | I5 | I6 | I7 | I8 | I9 | I10 | I11 | I12 | I13 | I14 | I15 | |
| CAV | 0.0000 | 0.0025 | 0.0000 | 0.0104 | 0.0090 | 0.0356 | 0.0491 | 0.0454 | 0.0617 | 0.0039 | 0.0030 | 0.0000 | 0.0163 | 0.0000 | 0.0000 | |
| GEX | 0.0118 | 0.0196 | 0.0192 | 0.0072 | 0.0115 | 0.0400 | 0.0405 | 0.0563 | 0.0627 | 0.0098 | 0.0236 | 0.0109 | 0.0045 | 0.0021 | 0.0679 | |
| GMD | 0.0006 | 0.0000 | 0.0016 | 0.0000 | 0.0062 | 0.0512 | 0.0344 | 0.0203 | 0.0242 | 0.0160 | 0.0320 | 0.1294 | 0.0000 | 0.0052 | 0.0366 | |
| GSP | 0.0021 | 0.0051 | 0.0134 | 0.0065 | 0.0066 | 0.0268 | 0.0346 | 0.0465 | 0.0000 | 0.0028 | 0.0135 | 0.0000 | 0.0114 | 0.0049 | 0.1147 | |
| HAH | 0.0000 | 0.0000 | 0.0000 | 0.0000 | 0.0080 | 0.0139 | 0.0027 | 0.0000 | 0.0252 | 0.0000 | 0.0050 | 0.1308 | 0.0093 | 0.0058 | 0.1186 | |
| L10 | 0.0070 | 0.0173 | 0.0131 | 0.0199 | 0.0076 | 0.0261 | 0.0120 | 0.0506 | 0.0598 | 0.0130 | 0.0547 | 0.0000 | 0.0141 | 0.0000 | 0.0093 | |
| MSN | 0.0047 | 0.0139 | 0.0065 | 0.0293 | 0.0144 | 0.0542 | 0.0434 | 0.0583 | 0.0605 | 0.0101 | 0.0000 | 0.0480 | 0.0049 | 0.0000 | 0.0405 | |
| PAC | 0.0000 | 0.0034 | 0.0029 | 0.0101 | 0.0114 | 0.0400 | 0.0545 | 0.0467 | 0.0604 | 0.0000 | 0.0000 | 0.0000 | 0.0143 | 0.0000 | 0.0000 | |
| PDN | 0.0000 | 0.0000 | 0.0000 | 0.0012 | 0.0073 | 0.0247 | 0.0170 | 0.0000 | 0.0000 | 0.0100 | 0.0203 | 0.0806 | 0.0045 | 0.0056 | 0.0054 | |
| PJT | 0.0061 | 0.0089 | 0.0167 | 0.0000 | 0.0118 | 0.0454 | 0.0450 | 0.0542 | 0.0435 | 0.0000 | 0.0000 | 0.0000 | 0.0087 | 0.0055 | 0.0600 | |
| PVP | 0.0003 | 0.0000 | 0.0078 | 0.0011 | 0.0103 | 0.0000 | 0.0000 | 0.0000 | 0.0000 | 0.0139 | 0.0549 | 0.0000 | 0.0136 | 0.0056 | 0.0366 | |
| PVT | 0.0023 | 0.0035 | 0.0061 | 0.0000 | 0.0081 | 0.0091 | 0.0217 | 0.0340 | 0.0000 | 0.0055 | 0.0303 | 0.0019 | 0.0065 | 0.0054 | 0.0913 | |
| REE | 0.0000 | 0.0000 | 0.0000 | 0.0000 | 0.0094 | 0.0078 | 0.0056 | 0.0441 | 0.0597 | 0.0092 | 0.0512 | 0.1400 | 0.0017 | 0.0066 | 0.1733 | |
| SRF | 0.0410 | 0.0434 | 0.0461 | 0.0000 | 0.0544 | 0.0407 | 0.0312 | 0.0766 | 0.0606 | 0.0162 | 0.0626 | 0.0000 | 0.0000 | 0.0000 | 0.0000 | |
| STG | 0.0019 | 0.0000 | 0.0070 | 0.0241 | 0.0083 | 0.0095 | 0.0000 | 0.0000 | 0.0382 | 0.0014 | 0.0363 | 0.0000 | 0.0053 | 0.0065 | 0.0000 | |
| SVI | 0.0000 | 0.0000 | 0.0000 | 0.0153 | 0.0071 | 0.0149 | 0.0282 | 0.0000 | 0.0542 | 0.0136 | 0.0470 | 0.0000 | 0.0129 | 0.0032 | 0.0000 | |
| TCL | 0.0000 | 0.0000 | 0.0000 | 0.0055 | 0.0100 | 0.0166 | 0.0107 | 0.0000 | 0.0000 | 0.0078 | 0.0342 | 0.0000 | 0.0051 | 0.0055 | 0.0000 | |
| TDP | 0.0016 | 0.0128 | 0.0133 | 0.0000 | 0.0116 | 0.0434 | 0.0447 | 0.0595 | 0.0618 | 0.0000 | 0.0188 | 0.0000 | 0.0121 | 0.0000 | 0.0000 | |
| THI | 0.0104 | 0.0169 | 0.0163 | 0.0456 | 0.0125 | 0.0017 | 0.0214 | 0.0599 | 0.0640 | 0.0115 | 0.0338 | 0.0000 | 0.0023 | 0.0049 | 0.0405 | |
| TMS | 0.0000 | 0.0000 | 0.0000 | 0.0520 | 0.0000 | 0.0101 | 0.0014 | 0.0062 | 0.0000 | 0.0033 | 0.0360 | 0.0000 | 0.0058 | 0.0066 | 0.0483 | |
| TV2 | 0.0097 | 0.0174 | 0.0170 | 0.0689 | 0.0146 | 0.0346 | 0.0176 | 0.0187 | 0.0578 | 0.0209 | 0.0674 | 0.0000 | 0.0000 | 0.0000 | 0.0000 | |
| VIP | 0.0000 | 0.0000 | 0.0020 | 0.0000 | 0.0000 | 0.0000 | 0.0000 | 0.0000 | 0.0527 | 0.0000 | 0.0290 | 0.0000 | 0.0033 | 0.0074 | 0.0000 | |
| VOS | 0.0000 | 0.0000 | 0.0005 | 0.0000 | 0.0140 | 0.0145 | 0.0229 | 0.0197 | 0.0451 | 0.0142 | 0.0147 | 0.0622 | 0.0069 | 0.0051 | 0.0679 | |
| VTB | 0.0041 | 0.0007 | 0.0130 | 0.0369 | 0.0000 | 0.0000 | 0.0005 | 0.0000 | 0.0607 | 0.0000 | 0.0235 | 0.0593 | 0.0012 | 0.0071 | 0.0483 | |
| VTO | 0.0073 | 0.0095 | 0.0175 | 0.0000 | 0.0154 | 0.0000 | 0.0000 | 0.0474 | 0.0509 | 0.0000 | 0.0000 | 0.0000 | 0.0000 | 0.0065 | 0.0991 | |
| ARM | 0.0071 | 0.0159 | 0.0171 | 0.0218 | 0.0064 | 0.0346 | 0.0161 | 0.0372 | 0.0480 | 0.0000 | 0.0461 | 0.0000 | 0.0000 | 0.0000 | 0.0000 | |
| BBS | 0.0066 | 0.0155 | 0.0142 | 0.0288 | 0.0108 | 0.0451 | 0.0331 | 0.0589 | 0.0557 | 0.0111 | 0.0597 | 0.0000 | 0.0108 | 0.0000 | 0.0000 | |
| CDN | 0.0000 | 0.0000 | 0.0064 | 0.0083 | 0.0095 | 0.0000 | 0.0129 | 0.0000 | 0.0184 | 0.0014 | 0.0243 | 0.1024 | 0.0000 | 0.0071 | 0.0913 | |
| CIA | 0.0000 | 0.0000 | 0.0015 | 0.0000 | 0.0251 | 0.0000 | 0.0000 | 0.0000 | 0.0451 | 0.0066 | 0.0308 | 0.0000 | 0.0000 | 0.0079 | 0.0000 | |
| CTB | 0.0000 | 0.0041 | 0.0000 | 0.0000 | 0.0080 | 0.0417 | 0.0403 | 0.0430 | 0.0619 | 0.0135 | 0.0508 | 0.0000 | 0.0068 | 0.0000 | 0.0000 | |
| CTT | 0.0000 | 0.0165 | 0.0118 | 0.0096 | 0.0101 | 0.0491 | 0.0365 | 0.0582 | 0.0395 | 0.0097 | 0.0370 | 0.0000 | 0.0091 | 0.0000 | 0.0000 | |
| DL1 | 0.0099 | 0.0163 | 0.0192 | 0.0000 | 0.0146 | 0.0383 | 0.0180 | 0.0604 | 0.0571 | 0.0136 | 0.0000 | 0.1326 | 0.0000 | 0.0048 | 0.1381 | |
| IPA | 0.0111 | 0.0183 | 0.0191 | 0.0086 | 0.0194 | 0.0000 | 0.0000 | 0.0620 | 0.0482 | 0.0000 | 0.0260 | 0.1743 | 0.0000 | 0.0065 | 0.2162 | |
| PBP | 0.0032 | 0.0057 | 0.0132 | 0.0108 | 0.0088 | 0.0322 | 0.0551 | 0.0000 | 0.0536 | 0.0000 | 0.0000 | 0.0000 | 0.0093 | 0.0000 | 0.0000 | |
| PMP | 0.0031 | 0.0133 | 0.0130 | 0.0232 | 0.0081 | 0.0437 | 0.0397 | 0.0574 | 0.0581 | 0.0000 | 0.0311 | 0.0000 | 0.0050 | 0.0000 | 0.0000 | |
| PMS | 0.0000 | 0.0010 | 0.0015 | 0.0000 | 0.0141 | 0.0366 | 0.0328 | 0.0242 | 0.0392 | 0.0000 | 0.0000 | 0.0000 | 0.0126 | 0.0008 | 0.0000 | |
| PRC | 0.0000 | 0.0000 | 0.0000 | 0.0000 | 0.0000 | 0.0000 | 0.0000 | 0.0000 | 0.0303 | 0.0000 | 0.0402 | 0.0000 | 0.0021 | 0.0062 | 0.0000 | |
| PSC | 0.0089 | 0.0152 | 0.0172 | 0.0000 | 0.0160 | 0.0559 | 0.0414 | 0.0519 | 0.0000 | 0.0000 | 0.0000 | 0.0000 | 0.0112 | 0.0038 | 0.0679 | |
| PTS | 0.0045 | 0.0117 | 0.0131 | 0.0000 | 0.0135 | 0.0559 | 0.0425 | 0.0568 | 0.0306 | 0.0041 | 0.0000 | 0.0000 | 0.0030 | 0.0018 | 0.0757 | |
| SDC | 0.0104 | 0.0160 | 0.0192 | 0.0193 | 0.0124 | 0.0054 | 0.0093 | 0.0279 | 0.0636 | 0.0107 | 0.0669 | 0.0286 | 0.0000 | 0.0037 | 0.0000 | |
| SDG | 0.0066 | 0.0113 | 0.0023 | 0.0092 | 0.0032 | 0.0366 | 0.0346 | 0.0497 | 0.0541 | 0.0000 | 0.0392 | 0.0000 | 0.0108 | 0.0012 | 0.0000 | |
| TPP | 0.0100 | 0.0188 | 0.0198 | 0.0000 | 0.0079 | 0.0430 | 0.0415 | 0.0608 | 0.0596 | 0.0103 | 0.0389 | 0.0000 | 0.0108 | 0.0000 | 0.0000 | |
| VBC | 0.0000 | 0.0044 | 0.0040 | 0.0196 | 0.0096 | 0.0376 | 0.0309 | 0.0495 | 0.0551 | 0.0090 | 0.0500 | 0.0000 | 0.0142 | 0.0000 | 0.0000 | |
| VCM | 0.0132 | 0.0207 | 0.0213 | 0.0475 | 0.0163 | 0.0000 | 0.0301 | 0.0000 | 0.0086 | 0.0144 | 0.0558 | 0.0117 | 0.0000 | 0.0056 | 0.0000 | |
| VSM | 0.0000 | 0.0000 | 0.0000 | 0.0000 | 0.0106 | 0.0237 | 0.0017 | 0.0000 | 0.0000 | 0.0057 | 0.0381 | 0.0000 | 0.0122 | 0.0036 | 0.0000 | |

**Table S26. The normalized sum of the weighted positive and negative distance from the average, the appraisal score, and rank in 2022 based on EDAS.**

| Company | NSP | NSN | AS | Rank |
| --- | --- | --- | --- | --- |
| CAV | 0.0714 | 0.6115 | 0.3414 | 16 |
| GEX | 0.0000 | 0.3644 | 0.1822 | 41 |
| GMD | 0.0116 | 0.4134 | 0.2125 | 39 |
| GSP | 0.0417 | 0.5264 | 0.2841 | 32 |
| HAH | 0.0921 | 0.4763 | 0.2842 | 31 |
| L10 | 0.0415 | 0.5005 | 0.2710 | 33 |
| MSN | 0.0398 | 0.3627 | 0.2012 | 40 |
| PAC | 0.1364 | 0.6004 | 0.3684 | 14 |
| PDN | 0.5530 | 0.7105 | 0.6317 | 3 |
| PJT | 0.1290 | 0.4985 | 0.3138 | 24 |
| PVP | 0.0929 | 0.7638 | 0.4284 | 11 |
| PVT | 0.0006 | 0.6300 | 0.3153 | 23 |
| REE | 0.0317 | 0.1661 | 0.0989 | 44 |
| SRF | 0.1156 | 0.2248 | 0.1702 | 42 |
| STG | 0.0117 | 0.7729 | 0.3923 | 13 |
| SVI | 0.1185 | 0.6779 | 0.3982 | 12 |
| TCL | 0.3430 | 0.8435 | 0.5933 | 5 |
| TDP | 0.0652 | 0.5415 | 0.3033 | 27 |
| THI | 0.0147 | 0.4398 | 0.2273 | 38 |
| TMS | 0.1756 | 0.7216 | 0.4486 | 9 |
| TV2 | 0.0702 | 0.4350 | 0.2526 | 37 |
| VIP | 0.2631 | 0.8452 | 0.5542 | 6 |
| VOS | 0.0490 | 0.5280 | 0.2885 | 29 |
| VTB | 0.3635 | 0.5813 | 0.4724 | 8 |
| VTO | 0.0512 | 0.5840 | 0.3176 | 22 |
| ARM | 0.0724 | 0.5896 | 0.3310 | 18 |
| BBS | 0.0815 | 0.4257 | 0.2536 | 36 |
| CDN | 0.0978 | 0.5376 | 0.3177 | 21 |
| CIA | 1.0000 | 0.8082 | 0.9041 | 1 |
| CTB | 0.0610 | 0.5573 | 0.3091 | 25 |
| CTT | 0.1151 | 0.5290 | 0.3221 | 19 |
| DL1 | 0.0733 | 0.1424 | 0.1079 | 43 |
| IPA | 0.1095 | 0.0000 | 0.0547 | 45 |
| PBP | 0.5143 | 0.6853 | 0.5998 | 4 |
| PMP | 0.0797 | 0.5152 | 0.2974 | 28 |
| PMS | 0.1253 | 0.7332 | 0.4293 | 10 |
| PRC | 0.5544 | 0.8709 | 0.7127 | 2 |
| PSC | 0.1672 | 0.5256 | 0.3464 | 15 |
| PTS | 0.0545 | 0.4865 | 0.2705 | 34 |
| SDC | 0.0542 | 0.5188 | 0.2865 | 30 |
| SDG | 0.0650 | 0.5756 | 0.3203 | 20 |
| TPP | 0.0472 | 0.4729 | 0.2601 | 35 |
| VBC | 0.0835 | 0.5343 | 0.3089 | 26 |
| VCM | 0.0746 | 0.5979 | 0.3363 | 17 |
| VSM | 0.2061 | 0.8432 | 0.5247 | 7 |

**Table S27. The weighted normalized decision matrix in 2020 based on TOPSIS.**

| Company | Indicator | | | | | | | | | | | | | | |  |
| --- | --- | --- | --- | --- | --- | --- | --- | --- | --- | --- | --- | --- | --- | --- | --- | --- |
|  | I1 | I2 | I3 | I4 | I5 | I6 | I7 | I8 | I9 | I10 | I11 | I12 | I13 | I14 | I15 | |
| CAV | 0.0121 | 0.0086 | 0.0185 | 0.0051 | -0.0020 | 0.0066 | 0.0077 | 0.0020 | 0.0004 | 0.0058 | 0.0077 | 0.0097 | 0.0019 | 0.0098 | 0.0210 | |
| GEX | 0.0049 | 0.0035 | 0.0045 | 0.0052 | -0.0001 | 0.0067 | 0.0097 | 0.0009 | 0.0003 | 0.0046 | 0.0053 | 0.0092 | 0.0052 | 0.0067 | 0.0136 | |
| GMD | 0.0029 | 0.0039 | 0.0032 | -0.0004 | -0.0017 | 0.0043 | 0.0078 | 0.0016 | 0.0012 | 0.0027 | 0.0067 | 0.0068 | 0.0189 | 0.0014 | 0.0129 | |
| GSP | 0.0068 | 0.0071 | 0.0044 | 0.0034 | 0.0002 | 0.0093 | 0.0082 | 0.0033 | 0.0055 | 0.0086 | 0.0159 | 0.0100 | 0.0035 | 0.0026 | 0.0133 | |
| HAH | 0.0055 | 0.0075 | 0.0074 | 0.0023 | 0.0002 | 0.0119 | 0.0185 | 0.0030 | 0.0011 | 0.0064 | 0.0082 | 0.0086 | 0.0082 | 0.0012 | 0.0100 | |
| L10 | 0.0038 | 0.0017 | 0.0041 | -0.0031 | 0.0009 | 0.0080 | 0.0123 | 0.0014 | 0.0003 | 0.0046 | 0.0031 | 0.0103 | 0.0021 | 0.0119 | 0.0174 | |
| MSN | 0.0017 | 0.0012 | 0.0029 | 0.0314 | -0.0056 | 0.0042 | 0.0042 | 0.0006 | 0.0003 | 0.0059 | 0.0428 | 0.0083 | 0.0057 | 0.0079 | 0.0102 | |
| PAC | 0.0110 | 0.0064 | 0.0080 | -0.0008 | -0.0005 | 0.0059 | 0.0022 | 0.0015 | 0.0002 | 0.0087 | 0.0356 | 0.0091 | 0.0024 | 0.0112 | 0.0229 | |
| PDN | 0.0136 | 0.0173 | 0.0213 | 0.0021 | 0.0006 | 0.0076 | 0.0128 | 0.0044 | 0.0480 | 0.0039 | 0.0076 | 0.0074 | 0.0082 | 0.0019 | 0.0160 | |
| PJT | 0.0062 | 0.0054 | 0.0041 | -0.0032 | -0.0006 | 0.0030 | 0.0030 | 0.0009 | 0.0009 | 0.0117 | 0.0341 | 0.0094 | 0.0062 | 0.0032 | 0.0121 | |
| PVP | 0.0086 | 0.0098 | 0.0067 | 0.0002 | 0.0033 | 0.0114 | 0.0187 | 0.0051 | 0.0077 | 0.0067 | 0.0060 | 0.0091 | 0.0022 | 0.0014 | 0.0102 | |
| PVT | 0.0058 | 0.0064 | 0.0055 | -0.0015 | 0.0004 | 0.0106 | 0.0119 | 0.0030 | 0.0031 | 0.0044 | 0.0082 | 0.0091 | 0.0049 | 0.0020 | 0.0119 | |
| REE | 0.0072 | 0.0086 | 0.0145 | 0.0047 | -0.0009 | 0.0096 | 0.0135 | 0.0020 | 0.0003 | 0.0043 | 0.0046 | 0.0077 | 0.0073 | 0.0014 | 0.0098 | |
| SRF | 0.0040 | 0.0022 | 0.0031 | -0.0039 | -0.0020 | 0.0059 | 0.0079 | 0.0007 | 0.0003 | 0.0029 | 0.0015 | 0.0099 | 0.0060 | 0.0127 | 0.0224 | |
| STG | 0.0033 | 0.0049 | 0.0030 | 0.0032 | -0.0008 | 0.0097 | 0.0164 | 0.0051 | 0.0023 | 0.0056 | 0.0050 | 0.0084 | 0.0106 | 0.0016 | 0.0174 | |
| SVI | 0.0147 | 0.0155 | 0.0283 | -0.0003 | 0.0003 | 0.0083 | 0.0101 | 0.0067 | 0.0006 | 0.0039 | 0.0042 | 0.0089 | 0.0026 | 0.0044 | 0.0224 | |
| TCL | 0.0073 | 0.0105 | 0.0075 | 0.0044 | -0.0008 | 0.0089 | 0.0099 | 0.0209 | 0.0079 | 0.0037 | 0.0063 | 0.0090 | 0.0081 | 0.0020 | 0.0186 | |
| TDP | 0.0063 | 0.0045 | 0.0037 | 0.0046 | 0.0001 | 0.0065 | 0.0035 | 0.0008 | 0.0001 | 0.0081 | 0.0079 | 0.0089 | 0.0046 | 0.0087 | 0.0214 | |
| THI | 0.0060 | 0.0049 | 0.0082 | -0.0018 | -0.0001 | 0.0076 | 0.0106 | 0.0010 | 0.0002 | 0.0043 | 0.0072 | 0.0089 | 0.0059 | 0.0059 | 0.0188 | |
| TMS | 0.0075 | 0.0092 | 0.0117 | 0.0139 | -0.0001 | 0.0071 | 0.0122 | 0.0028 | 0.0346 | 0.0075 | 0.0099 | 0.0098 | 0.0041 | 0.0022 | 0.0155 | |
| TV2 | 0.0129 | 0.0098 | 0.0181 | 0.0002 | 0.0001 | 0.0087 | 0.0123 | 0.0320 | 0.0005 | 0.0022 | 0.0047 | 0.0094 | 0.0054 | 0.0075 | 0.0188 | |
| VIP | 0.0030 | 0.0045 | 0.0020 | -0.0051 | 0.0070 | 0.0154 | 0.0140 | 0.0021 | 0.0003 | 0.0076 | 0.0066 | 0.0091 | 0.0169 | 0.0008 | 0.0079 | |
| VOS | -0.0159 | -0.0067 | -0.0037 | -0.0052 | -0.0341 | 0.0050 | 0.0077 | -0.0002 | 0.0009 | 0.0029 | 0.0063 | 0.0110 | 0.0099 | 0.0092 | 0.0095 | |
| VTB | 0.0049 | 0.0065 | 0.0041 | 0.0081 | 0.0021 | 0.0128 | 0.0101 | 0.0117 | 0.0002 | 0.0121 | 0.0097 | 0.0076 | 0.0056 | 0.0020 | 0.0205 | |
| VTO | 0.0032 | 0.0039 | 0.0018 | -0.0071 | -0.0005 | 0.0084 | 0.0137 | 0.0014 | 0.0005 | 0.0068 | 0.0258 | 0.0090 | 0.0098 | 0.0016 | 0.0124 | |
| ARM | 0.0023 | 0.0010 | 0.0014 | -0.0117 | -0.0039 | 0.0064 | 0.0117 | 0.0007 | 0.0005 | 0.0052 | 0.0048 | 0.0092 | 0.0148 | 0.0218 | 0.0236 | |
| BBS | 0.0033 | 0.0018 | 0.0019 | 0.0025 | 0.0007 | 0.0051 | 0.0082 | 0.0006 | 0.0005 | 0.0034 | 0.0022 | 0.0095 | 0.0074 | 0.0125 | 0.0224 | |
| CDN | 0.0078 | 0.0131 | 0.0054 | 0.0030 | 0.0002 | 0.0275 | 0.0159 | 0.0086 | 0.0022 | 0.0097 | 0.0073 | 0.0069 | 0.0116 | 0.0005 | 0.0105 | |
| CIA | -0.0056 | -0.0093 | -0.0054 | -0.0200 | -0.0360 | 0.0151 | 0.0305 | -0.0046 | 0.0011 | 0.0044 | 0.0036 | 0.0122 | 0.0332 | 0.0013 | 0.0205 | |
| CTB | 0.0078 | 0.0067 | 0.0067 | -0.0063 | 0.0030 | 0.0081 | 0.0080 | 0.0054 | 0.0002 | 0.0024 | 0.0022 | 0.0081 | 0.0134 | 0.0068 | 0.0217 | |
| CTT | 0.0083 | 0.0020 | 0.0038 | -0.0002 | 0.0025 | 0.0042 | 0.0050 | 0.0007 | 0.0007 | 0.0041 | 0.0066 | 0.0100 | 0.0068 | 0.0377 | 0.0224 | |
| DL1 | 0.0017 | 0.0028 | 0.0010 | -0.0019 | 0.0086 | 0.0062 | 0.0124 | 0.0013 | 0.0097 | 0.0024 | 0.0007 | 0.0105 | 0.0021 | 0.0010 | 0.0236 | |
| IPA | 0.0047 | 0.0051 | 0.0052 | 0.0026 | 0.0022 | 0.0114 | 0.0230 | 0.0010 | 0.0003 | 0.0037 | 0.0080 | 0.0065 | 0.0237 | 0.0020 | 0.0081 | |
| PBP | 0.0046 | 0.0049 | 0.0027 | 0.0075 | -0.0010 | 0.0080 | 0.0028 | 0.0020 | 0.0003 | 0.0055 | 0.0208 | 0.0096 | 0.0063 | 0.0029 | 0.0207 | |
| PMP | 0.0050 | 0.0028 | 0.0034 | 0.0040 | 0.0024 | 0.0056 | 0.0054 | 0.0008 | 0.0003 | 0.0070 | 0.0061 | 0.0096 | 0.0065 | 0.0132 | 0.0221 | |
| PMS | 0.0070 | 0.0071 | 0.0078 | -0.0047 | 0.0029 | 0.0061 | 0.0103 | 0.0031 | 0.0013 | 0.0086 | 0.0074 | 0.0097 | 0.0064 | 0.0051 | 0.0231 | |
| PRC | 0.0007 | 0.0006 | 0.0009 | -0.0059 | -0.0042 | 0.0049 | 0.0083 | 0.0005 | 0.0015 | 0.0070 | 0.0049 | 0.0098 | 0.0074 | 0.0042 | 0.0171 | |
| PSC | 0.0049 | 0.0044 | 0.0041 | -0.0070 | 0.0000 | 0.0034 | 0.0056 | 0.0014 | 0.0042 | 0.0093 | 0.0177 | 0.0094 | 0.0044 | 0.0024 | 0.0088 | |
| PTS | 0.0040 | 0.0026 | 0.0035 | -0.0021 | 0.0031 | 0.0031 | 0.0036 | 0.0006 | 0.0007 | 0.0046 | 0.0249 | 0.0092 | 0.0094 | 0.0049 | 0.0107 | |
| SDC | 0.0013 | 0.0014 | 0.0008 | -0.0078 | -0.0005 | 0.0110 | 0.0147 | 0.0010 | 0.0001 | 0.0039 | 0.0007 | 0.0086 | 0.0226 | 0.0037 | 0.0210 | |
| SDG | 0.0066 | 0.0077 | 0.0197 | 0.0102 | -0.0006 | 0.0062 | 0.0063 | 0.0025 | 0.0008 | 0.0447 | 0.0144 | 0.0093 | 0.0056 | 0.0044 | 0.0231 | |
| TPP | 0.0021 | 0.0011 | 0.0012 | 0.0007 | 0.0113 | 0.0056 | 0.0034 | 0.0005 | 0.0002 | 0.0099 | 0.0062 | 0.0087 | 0.0072 | 0.0120 | 0.0188 | |
| VBC | 0.0110 | 0.0063 | 0.0081 | -0.0023 | 0.0003 | 0.0064 | 0.0090 | 0.0015 | 0.0005 | 0.0051 | 0.0036 | 0.0097 | 0.0027 | 0.0106 | 0.0229 | |
| VCM | 0.0058 | 0.0076 | 0.0075 | -0.0213 | 0.0140 | 0.0144 | 0.0075 | 0.0422 | 0.0006 | 0.0013 | 0.0022 | 0.0069 | 0.0407 | 0.0016 | 0.0190 | |
| VSM | 0.0087 | 0.0098 | 0.0072 | 0.0034 | 0.0003 | 0.0080 | 0.0153 | 0.0048 | 0.0074 | 0.0045 | 0.0054 | 0.0097 | 0.0045 | 0.0038 | 0.0205 | |
| **PIS** | **0.0147** | **0.0173** | **0.0283** | **0.0314** | **0.0140** | **0.0275** | **0.0305** | **0.0422** | **0.0480** | **0.0013** | **0.0428** | **0.0065** | **0.0019** | **0.0005** | **0.0079** | |
| **NIS** | **-0.0159** | **-0.0093** | **-0.0054** | **-0.0213** | **-0.0360** | **0.0030** | **0.0022** | **-0.0046** | **0.0001** | **0.0447** | **0.0007** | **0.0122** | **0.0407** | **0.0377** | **0.0236** | |

**Table S28. The normalized decision matrix in 2021 based on TOPSIS.**

| Company | Indicator | | | | | | | | | | | | | | |  |
| --- | --- | --- | --- | --- | --- | --- | --- | --- | --- | --- | --- | --- | --- | --- | --- | --- |
|  | I1 | I2 | I3 | I4 | I5 | I6 | I7 | I8 | I9 | I10 | I11 | I12 | I13 | I14 | I15 | |
| CAV | 0.1526 | 0.0992 | 0.2411 | -0.0039 | -0.0134 | 0.0921 | 0.0261 | 0.0277 | 0.0027 | 0.0763 | 0.0712 | 0.1558 | 0.0215 | 0.2225 | 0.1838 | |
| GEX | 0.0585 | 0.0426 | 0.0512 | 0.2797 | 0.0067 | 0.1032 | 0.0904 | 0.0155 | 0.0026 | 0.0877 | 0.0631 | 0.1455 | 0.0707 | 0.1008 | 0.1119 | |
| GMD | 0.0726 | 0.1078 | 0.0807 | 0.1092 | 0.0316 | 0.0595 | 0.0822 | 0.0426 | 0.0224 | 0.0682 | 0.0723 | 0.1106 | 0.1441 | 0.0289 | 0.1218 | |
| GSP | 0.0809 | 0.0933 | 0.0407 | 0.0258 | -0.0110 | 0.1310 | 0.1226 | 0.0472 | 0.0678 | 0.1459 | 0.1082 | 0.1606 | 0.0354 | 0.0450 | 0.1019 | |
| HAH | 0.2225 | 0.3030 | 0.3712 | 0.3036 | 0.1237 | 0.1643 | 0.2197 | 0.1186 | 0.0209 | 0.1844 | 0.0902 | 0.1090 | 0.0682 | 0.0293 | 0.0939 | |
| L10 | 0.0474 | 0.0230 | 0.0500 | -0.0509 | -0.0088 | 0.1135 | 0.1205 | 0.0181 | 0.0032 | 0.0684 | 0.0286 | 0.1664 | 0.0136 | 0.2693 | 0.1478 | |
| MSN | 0.2055 | 0.1282 | 0.3061 | 0.0656 | 0.5143 | 0.1000 | 0.1022 | 0.0190 | 0.0041 | 0.1092 | 0.3431 | 0.1288 | 0.0719 | 0.0734 | 0.0819 | |
| PAC | 0.1759 | 0.1289 | 0.1421 | 0.0112 | 0.0137 | 0.0929 | 0.0233 | 0.0321 | 0.0030 | 0.2189 | 0.4064 | 0.1466 | 0.0267 | 0.1602 | 0.1917 | |
| PDN | 0.1893 | 0.2699 | 0.3283 | 0.0591 | -0.0099 | 0.1072 | 0.1206 | 0.0934 | 0.7723 | 0.0835 | 0.0766 | 0.1201 | 0.0845 | 0.0348 | 0.1338 | |
| PJT | 0.0678 | 0.0775 | 0.0347 | 0.0163 | -0.0198 | 0.0651 | 0.0482 | 0.0135 | 0.0115 | 0.3020 | 0.2808 | 0.1543 | 0.0636 | 0.0302 | 0.0879 | |
| PVP | 0.1045 | 0.1491 | 0.0864 | -0.1079 | 0.0080 | 0.2024 | 0.2816 | 0.0904 | 0.0526 | 0.0690 | 0.0279 | 0.1360 | 0.0295 | 0.0217 | 0.0979 | |
| PVT | 0.0805 | 0.1014 | 0.0827 | 0.0050 | -0.0005 | 0.1572 | 0.1374 | 0.0455 | 0.0387 | 0.0935 | 0.0629 | 0.1432 | 0.0583 | 0.0316 | 0.0879 | |
| REE | 0.1050 | 0.1284 | 0.2525 | 0.0144 | 0.0201 | 0.1334 | 0.1468 | 0.0253 | 0.0034 | 0.0661 | 0.0302 | 0.1035 | 0.0999 | 0.0223 | 0.0519 | |
| SRF | 0.0429 | 0.0281 | 0.0205 | -0.1772 | 0.0267 | 0.0897 | 0.0869 | 0.0112 | 0.0022 | 0.0353 | 0.0103 | 0.1558 | 0.0737 | 0.1854 | 0.1838 | |
| STG | 0.1066 | 0.1679 | 0.0979 | 0.1992 | 0.0455 | 0.1389 | 0.1682 | 0.1141 | 0.0219 | 0.1501 | 0.0622 | 0.1427 | 0.0729 | 0.0272 | 0.1418 | |
| SVI | 0.1240 | 0.1483 | 0.3029 | 0.0425 | -0.0405 | 0.1310 | 0.1183 | 0.1108 | 0.0071 | 0.0661 | 0.0386 | 0.1500 | 0.0345 | 0.0728 | 0.1937 | |
| TCL | 0.1509 | 0.2238 | 0.1315 | 0.0417 | 0.0036 | 0.1246 | 0.1282 | 0.5081 | 0.1231 | 0.0711 | 0.0579 | 0.1422 | 0.0816 | 0.0384 | 0.1598 | |
| TDP | 0.1039 | 0.0688 | 0.0608 | 0.3665 | -0.0313 | 0.0834 | 0.0505 | 0.0111 | 0.0019 | 0.1758 | 0.1161 | 0.1499 | 0.0402 | 0.2123 | 0.1678 | |
| THI | 0.0471 | 0.0451 | 0.0695 | -0.0709 | -0.0303 | 0.1159 | 0.0828 | 0.0127 | 0.0020 | 0.0623 | 0.0666 | 0.1489 | 0.0719 | 0.0854 | 0.1518 | |
| TMS | 0.1581 | 0.2141 | 0.2274 | 0.4110 | 0.0032 | 0.1175 | 0.1667 | 0.0750 | 0.5948 | 0.1881 | 0.1007 | 0.1570 | 0.0392 | 0.0423 | 0.1458 | |
| TV2 | 0.1728 | 0.1262 | 0.2293 | 0.0400 | -0.0043 | 0.0945 | 0.1226 | 0.3304 | 0.0113 | 0.0240 | 0.0178 | 0.1561 | 0.0584 | 0.1986 | 0.1957 | |
| VIP | 0.0078 | 0.0123 | 0.0065 | 0.1509 | -0.0840 | 0.3239 | 0.1239 | 0.0104 | 0.0055 | 0.1823 | 0.0731 | 0.1600 | 0.1137 | 0.0152 | 0.0939 | |
| VOS | 0.5224 | 0.3204 | 0.1478 | 0.0527 | -0.3220 | 0.1191 | 0.1017 | 0.0328 | 0.0093 | 0.0376 | 0.0682 | 0.1171 | 0.1158 | 0.0648 | 0.0839 | |
| VTB | 0.0186 | 0.0295 | 0.0147 | -0.1381 | -0.0575 | 0.2072 | 0.1123 | 0.5337 | 0.0024 | 0.3739 | 0.0750 | 0.1295 | 0.0767 | 0.0263 | 0.1618 | |
| VTO | 0.0930 | 0.1322 | 0.0594 | -0.0892 | 0.1225 | 0.1651 | 0.2229 | 0.0432 | 0.0069 | 0.1658 | 0.1806 | 0.1481 | 0.1126 | 0.0283 | 0.1178 | |
| ARM | 0.0429 | 0.0223 | 0.0236 | 0.0322 | 0.0041 | 0.1056 | 0.1403 | 0.0119 | 0.0081 | 0.1081 | 0.0401 | 0.1506 | 0.1305 | 0.2022 | 0.1957 | |
| BBS | 0.0760 | 0.0475 | 0.0570 | 0.0317 | 0.0403 | 0.0794 | 0.0984 | 0.0111 | 0.0074 | 0.0777 | 0.0214 | 0.1577 | 0.0321 | 0.2287 | 0.1977 | |
| CDN | 0.1359 | 0.2505 | 0.0953 | 0.0914 | -0.0027 | 0.3747 | 0.1753 | 0.1693 | 0.0263 | 0.2099 | 0.0800 | 0.1100 | 0.1331 | 0.0104 | 0.1119 | |
| CIA | -0.1120 | -0.1949 | -0.0853 | -0.2787 | 0.1539 | 0.2040 | 0.2982 | -0.0780 | 0.0069 | 0.0486 | 0.0166 | 0.2833 | 0.7187 | 0.0213 | 0.1658 | |
| CTB | 0.0901 | 0.0723 | 0.0778 | 0.1518 | -0.0408 | 0.1604 | 0.0755 | 0.0193 | 0.0020 | 0.0652 | 0.0331 | 0.1484 | 0.1054 | 0.1183 | 0.1238 | |
| CTT | 0.1296 | 0.0335 | 0.0541 | 0.1832 | -0.0236 | 0.0643 | 0.0688 | 0.0103 | 0.0126 | 0.1025 | 0.0759 | 0.1628 | 0.0559 | 0.6404 | 0.1897 | |
| DL1 | 0.0323 | 0.0491 | 0.0189 | -0.2208 | 0.1345 | 0.1151 | 0.1683 | 0.0257 | 0.0054 | 0.0225 | 0.0322 | 0.1615 | 0.0274 | 0.0390 | 0.1158 | |
| IPA | 0.4451 | 0.4508 | 0.3689 | 0.0359 | 0.6713 | 0.3199 | 0.4895 | 0.0479 | 0.0117 | 0.0997 | 0.0585 | 0.0783 | 0.2352 | 0.0248 | 0.0439 | |
| PBP | 0.0746 | 0.1021 | 0.0434 | 0.0852 | -0.0112 | 0.1294 | 0.0689 | 0.0744 | 0.0059 | 0.1740 | 0.6085 | 0.1563 | 0.0684 | 0.0515 | 0.1858 | |
| PMP | 0.0832 | 0.0489 | 0.0574 | 0.2247 | -0.0251 | 0.0818 | 0.0543 | 0.0124 | 0.0046 | 0.2026 | 0.0871 | 0.1554 | 0.0672 | 0.2469 | 0.1917 | |
| PMS | 0.1217 | 0.1360 | 0.1298 | 0.0008 | 0.0136 | 0.0929 | 0.0801 | 0.0657 | 0.0120 | 0.1557 | 0.0725 | 0.1560 | 0.0634 | 0.0881 | 0.1977 | |
| PRC | 0.0379 | 0.0404 | 0.0469 | -0.0054 | 0.2478 | 0.0786 | 0.0946 | 0.0111 | 0.0179 | 0.1802 | 0.0512 | 0.1557 | 0.0798 | 0.0615 | 0.1278 | |
| PSC | 0.0518 | 0.0522 | 0.0392 | 0.0171 | -0.0347 | 0.0516 | 0.0657 | 0.0172 | 0.0655 | 0.1831 | 0.1908 | 0.1549 | 0.0438 | 0.0421 | 0.0819 | |
| PTS | 0.0971 | 0.0746 | 0.0790 | 0.0417 | 0.0453 | 0.0564 | 0.0571 | 0.0143 | 0.0127 | 0.1091 | 0.2246 | 0.1475 | 0.1050 | 0.0707 | 0.0919 | |
| SDC | 0.0361 | 0.0469 | 0.0288 | -0.0434 | 0.0912 | 0.1794 | 0.1861 | 0.0450 | 0.0016 | 0.0819 | 0.0063 | 0.1341 | 0.3258 | 0.0504 | 0.1738 | |
| SDG | 0.0344 | 0.0406 | 0.0747 | -0.0735 | -0.0583 | 0.0961 | 0.0657 | 0.0150 | 0.0069 | 0.3374 | 0.0656 | 0.1543 | 0.0579 | 0.0779 | 0.1878 | |
| TPP | 0.0242 | 0.0141 | 0.0116 | 0.2266 | -0.0341 | 0.0865 | 0.0738 | 0.0065 | 0.0036 | 0.1797 | 0.0593 | 0.1405 | 0.0470 | 0.1982 | 0.1578 | |
| VBC | 0.1648 | 0.1188 | 0.1265 | 0.0578 | -0.0091 | 0.0976 | 0.1014 | 0.0232 | 0.0072 | 0.0999 | 0.0403 | 0.1573 | 0.0272 | 0.1801 | 0.1957 | |
| VCM | 0.0109 | 0.0181 | 0.0137 | 0.0317 | -0.0853 | 0.2191 | 0.0888 | 0.3929 | 0.0425 | 0.0714 | 0.0347 | 0.1367 | 0.3016 | 0.0242 | 0.1858 | |
| VSM | 0.1806 | 0.2211 | 0.1097 | 0.1995 | 0.0085 | 0.1207 | 0.1787 | 0.2579 | 0.1248 | 0.0970 | 0.0556 | 0.1531 | 0.0514 | 0.0685 | 0.1897 | |

**Table S29. The weighted normalized decision matrix in 2021 based on TOPSIS.**

| Company | Indicator | | | | | | | | | | | | | | |  |
| --- | --- | --- | --- | --- | --- | --- | --- | --- | --- | --- | --- | --- | --- | --- | --- | --- |
|  | I1 | I2 | I3 | I4 | I5 | I6 | I7 | I8 | I9 | I10 | I11 | I12 | I13 | I14 | I15 | |
| CAV | 0.0073 | 0.0041 | 0.0157 | -0.0003 | -0.0007 | 0.0071 | 0.0015 | 0.0022 | 0.0002 | 0.0072 | 0.0055 | 0.0062 | 0.0014 | 0.0138 | 0.0175 | |
| GEX | 0.0028 | 0.0018 | 0.0033 | 0.0213 | 0.0003 | 0.0080 | 0.0053 | 0.0013 | 0.0002 | 0.0083 | 0.0049 | 0.0058 | 0.0046 | 0.0063 | 0.0107 | |
| GMD | 0.0035 | 0.0045 | 0.0053 | 0.0083 | 0.0017 | 0.0046 | 0.0048 | 0.0034 | 0.0015 | 0.0064 | 0.0056 | 0.0044 | 0.0093 | 0.0018 | 0.0116 | |
| GSP | 0.0038 | 0.0039 | 0.0027 | 0.0020 | -0.0006 | 0.0102 | 0.0072 | 0.0038 | 0.0045 | 0.0137 | 0.0084 | 0.0064 | 0.0023 | 0.0028 | 0.0097 | |
| HAH | 0.0106 | 0.0126 | 0.0242 | 0.0231 | 0.0065 | 0.0127 | 0.0129 | 0.0096 | 0.0014 | 0.0174 | 0.0070 | 0.0043 | 0.0044 | 0.0018 | 0.0089 | |
| L10 | 0.0023 | 0.0010 | 0.0033 | -0.0039 | -0.0005 | 0.0088 | 0.0071 | 0.0015 | 0.0002 | 0.0064 | 0.0022 | 0.0066 | 0.0009 | 0.0167 | 0.0141 | |
| MSN | 0.0098 | 0.0053 | 0.0200 | 0.0050 | 0.0269 | 0.0078 | 0.0060 | 0.0015 | 0.0003 | 0.0103 | 0.0267 | 0.0051 | 0.0046 | 0.0046 | 0.0078 | |
| PAC | 0.0084 | 0.0054 | 0.0093 | 0.0009 | 0.0007 | 0.0072 | 0.0014 | 0.0026 | 0.0002 | 0.0206 | 0.0317 | 0.0058 | 0.0017 | 0.0099 | 0.0183 | |
| PDN | 0.0090 | 0.0112 | 0.0214 | 0.0045 | -0.0005 | 0.0083 | 0.0071 | 0.0075 | 0.0517 | 0.0079 | 0.0060 | 0.0048 | 0.0054 | 0.0022 | 0.0127 | |
| PJT | 0.0032 | 0.0032 | 0.0023 | 0.0012 | -0.0010 | 0.0050 | 0.0028 | 0.0011 | 0.0008 | 0.0284 | 0.0219 | 0.0061 | 0.0041 | 0.0019 | 0.0084 | |
| PVP | 0.0050 | 0.0062 | 0.0056 | -0.0082 | 0.0004 | 0.0157 | 0.0166 | 0.0073 | 0.0035 | 0.0065 | 0.0022 | 0.0054 | 0.0019 | 0.0013 | 0.0093 | |
| PVT | 0.0038 | 0.0042 | 0.0054 | 0.0004 | 0.0000 | 0.0122 | 0.0081 | 0.0037 | 0.0026 | 0.0088 | 0.0049 | 0.0057 | 0.0038 | 0.0020 | 0.0084 | |
| REE | 0.0050 | 0.0053 | 0.0165 | 0.0011 | 0.0011 | 0.0103 | 0.0086 | 0.0020 | 0.0002 | 0.0062 | 0.0023 | 0.0041 | 0.0064 | 0.0014 | 0.0049 | |
| SRF | 0.0020 | 0.0012 | 0.0013 | -0.0135 | 0.0014 | 0.0070 | 0.0051 | 0.0009 | 0.0001 | 0.0033 | 0.0008 | 0.0062 | 0.0047 | 0.0115 | 0.0175 | |
| STG | 0.0051 | 0.0070 | 0.0064 | 0.0152 | 0.0024 | 0.0108 | 0.0099 | 0.0092 | 0.0015 | 0.0141 | 0.0048 | 0.0057 | 0.0047 | 0.0017 | 0.0135 | |
| SVI | 0.0059 | 0.0062 | 0.0198 | 0.0032 | -0.0021 | 0.0102 | 0.0070 | 0.0089 | 0.0005 | 0.0062 | 0.0030 | 0.0060 | 0.0022 | 0.0045 | 0.0184 | |
| TCL | 0.0072 | 0.0093 | 0.0086 | 0.0032 | 0.0002 | 0.0097 | 0.0075 | 0.0409 | 0.0082 | 0.0067 | 0.0045 | 0.0056 | 0.0053 | 0.0024 | 0.0152 | |
| TDP | 0.0049 | 0.0029 | 0.0040 | 0.0279 | -0.0016 | 0.0065 | 0.0030 | 0.0009 | 0.0001 | 0.0166 | 0.0090 | 0.0059 | 0.0026 | 0.0132 | 0.0160 | |
| THI | 0.0022 | 0.0019 | 0.0045 | -0.0054 | -0.0016 | 0.0090 | 0.0049 | 0.0010 | 0.0001 | 0.0059 | 0.0052 | 0.0059 | 0.0046 | 0.0053 | 0.0145 | |
| TMS | 0.0075 | 0.0089 | 0.0148 | 0.0313 | 0.0002 | 0.0091 | 0.0098 | 0.0060 | 0.0398 | 0.0177 | 0.0078 | 0.0062 | 0.0025 | 0.0026 | 0.0139 | |
| TV2 | 0.0082 | 0.0053 | 0.0149 | 0.0031 | -0.0002 | 0.0073 | 0.0072 | 0.0266 | 0.0008 | 0.0023 | 0.0014 | 0.0062 | 0.0038 | 0.0123 | 0.0186 | |
| VIP | 0.0004 | 0.0005 | 0.0004 | 0.0115 | -0.0044 | 0.0251 | 0.0073 | 0.0008 | 0.0004 | 0.0172 | 0.0057 | 0.0063 | 0.0073 | 0.0009 | 0.0089 | |
| VOS | 0.0248 | 0.0133 | 0.0096 | 0.0040 | -0.0168 | 0.0092 | 0.0060 | 0.0026 | 0.0006 | 0.0035 | 0.0053 | 0.0046 | 0.0075 | 0.0040 | 0.0080 | |
| VTB | 0.0009 | 0.0012 | 0.0010 | -0.0105 | -0.0030 | 0.0161 | 0.0066 | 0.0430 | 0.0002 | 0.0352 | 0.0058 | 0.0051 | 0.0049 | 0.0016 | 0.0154 | |
| VTO | 0.0044 | 0.0055 | 0.0039 | -0.0068 | 0.0064 | 0.0128 | 0.0131 | 0.0035 | 0.0005 | 0.0156 | 0.0141 | 0.0059 | 0.0072 | 0.0018 | 0.0112 | |
| ARM | 0.0020 | 0.0009 | 0.0015 | 0.0025 | 0.0002 | 0.0082 | 0.0083 | 0.0010 | 0.0005 | 0.0102 | 0.0031 | 0.0060 | 0.0084 | 0.0125 | 0.0186 | |
| BBS | 0.0036 | 0.0020 | 0.0037 | 0.0024 | 0.0021 | 0.0062 | 0.0058 | 0.0009 | 0.0005 | 0.0073 | 0.0017 | 0.0063 | 0.0021 | 0.0142 | 0.0188 | |
| CDN | 0.0065 | 0.0104 | 0.0062 | 0.0070 | -0.0001 | 0.0290 | 0.0103 | 0.0136 | 0.0018 | 0.0198 | 0.0062 | 0.0044 | 0.0086 | 0.0006 | 0.0107 | |
| CIA | -0.0053 | -0.0081 | -0.0056 | -0.0212 | 0.0080 | 0.0158 | 0.0175 | -0.0063 | 0.0005 | 0.0046 | 0.0013 | 0.0112 | 0.0463 | 0.0013 | 0.0158 | |
| CTB | 0.0043 | 0.0030 | 0.0051 | 0.0116 | -0.0021 | 0.0124 | 0.0044 | 0.0016 | 0.0001 | 0.0061 | 0.0026 | 0.0059 | 0.0068 | 0.0073 | 0.0118 | |
| CTT | 0.0062 | 0.0014 | 0.0035 | 0.0140 | -0.0012 | 0.0050 | 0.0040 | 0.0008 | 0.0008 | 0.0097 | 0.0059 | 0.0065 | 0.0036 | 0.0397 | 0.0181 | |
| DL1 | 0.0015 | 0.0020 | 0.0012 | -0.0168 | 0.0070 | 0.0089 | 0.0099 | 0.0021 | 0.0004 | 0.0021 | 0.0025 | 0.0064 | 0.0018 | 0.0024 | 0.0110 | |
| IPA | 0.0212 | 0.0187 | 0.0241 | 0.0027 | 0.0351 | 0.0248 | 0.0288 | 0.0039 | 0.0008 | 0.0094 | 0.0046 | 0.0031 | 0.0151 | 0.0015 | 0.0042 | |
| PBP | 0.0035 | 0.0042 | 0.0028 | 0.0065 | -0.0006 | 0.0100 | 0.0041 | 0.0060 | 0.0004 | 0.0164 | 0.0474 | 0.0062 | 0.0044 | 0.0032 | 0.0177 | |
| PMP | 0.0040 | 0.0020 | 0.0037 | 0.0171 | -0.0013 | 0.0063 | 0.0032 | 0.0010 | 0.0003 | 0.0191 | 0.0068 | 0.0062 | 0.0043 | 0.0153 | 0.0183 | |
| PMS | 0.0058 | 0.0057 | 0.0085 | 0.0001 | 0.0007 | 0.0072 | 0.0047 | 0.0053 | 0.0008 | 0.0147 | 0.0056 | 0.0062 | 0.0041 | 0.0055 | 0.0188 | |
| PRC | 0.0018 | 0.0017 | 0.0031 | -0.0004 | 0.0129 | 0.0061 | 0.0056 | 0.0009 | 0.0012 | 0.0170 | 0.0040 | 0.0062 | 0.0051 | 0.0038 | 0.0122 | |
| PSC | 0.0025 | 0.0022 | 0.0026 | 0.0013 | -0.0018 | 0.0040 | 0.0039 | 0.0014 | 0.0044 | 0.0172 | 0.0149 | 0.0061 | 0.0028 | 0.0026 | 0.0078 | |
| PTS | 0.0046 | 0.0031 | 0.0052 | 0.0032 | 0.0024 | 0.0044 | 0.0034 | 0.0011 | 0.0008 | 0.0103 | 0.0175 | 0.0058 | 0.0068 | 0.0044 | 0.0087 | |
| SDC | 0.0017 | 0.0020 | 0.0019 | -0.0033 | 0.0048 | 0.0139 | 0.0109 | 0.0036 | 0.0001 | 0.0077 | 0.0005 | 0.0053 | 0.0210 | 0.0031 | 0.0165 | |
| SDG | 0.0016 | 0.0017 | 0.0049 | -0.0056 | -0.0030 | 0.0074 | 0.0039 | 0.0012 | 0.0005 | 0.0318 | 0.0051 | 0.0061 | 0.0037 | 0.0048 | 0.0179 | |
| TPP | 0.0011 | 0.0006 | 0.0008 | 0.0173 | -0.0018 | 0.0067 | 0.0043 | 0.0005 | 0.0002 | 0.0169 | 0.0046 | 0.0056 | 0.0030 | 0.0123 | 0.0150 | |
| VBC | 0.0078 | 0.0049 | 0.0083 | 0.0044 | -0.0005 | 0.0076 | 0.0060 | 0.0019 | 0.0005 | 0.0094 | 0.0031 | 0.0062 | 0.0018 | 0.0112 | 0.0186 | |
| VCM | 0.0005 | 0.0008 | 0.0009 | 0.0024 | -0.0045 | 0.0170 | 0.0052 | 0.0317 | 0.0028 | 0.0067 | 0.0027 | 0.0054 | 0.0194 | 0.0015 | 0.0177 | |
| VSM | 0.0086 | 0.0092 | 0.0072 | 0.0152 | 0.0004 | 0.0094 | 0.0105 | 0.0208 | 0.0084 | 0.0091 | 0.0043 | 0.0061 | 0.0033 | 0.0042 | 0.0181 | |
| **PIS** | **0.0248** | **0.0187** | **0.0242** | **0.0313** | **0.0351** | **0.0290** | **0.0288** | **0.0430** | **0.0517** | **0.0021** | **0.0474** | **0.0031** | **0.0009** | **0.0006** | **0.0042** | |
| **NIS** | **-0.0053** | **-0.0081** | **-0.0056** | **-0.0212** | **-0.0168** | **0.0040** | **0.0014** | **-0.0063** | **0.0001** | **0.0352** | **0.0005** | **0.0112** | **0.0463** | **0.0397** | **0.0188** | |

**Table S30. The distance from each alternative to PIS and NIS, and rank in 2021 based on TOPSIS.**

| Company | S_i*_ | S_i-_ | Score | Rank |
| --- | --- | --- | --- | --- |
| CAV | 0.1027 | 0.0510 | 0.3316 | 22 |
| GEX | 0.0992 | 0.0592 | 0.3737 | 13 |
| GMD | 0.0991 | 0.0530 | 0.3484 | 18 |
| GSP | 0.0977 | 0.0453 | 0.3169 | 32 |
| HAH | 0.0848 | 0.0731 | 0.4628 | 4 |
| L10 | 0.1070 | 0.0442 | 0.2924 | 40 |
| MSN | 0.0840 | 0.0700 | 0.4543 | 6 |
| PAC | 0.0958 | 0.0449 | 0.3192 | 31 |
| PDN | 0.0794 | 0.0799 | 0.5015 | 3 |
| PJT | 0.1019 | 0.0373 | 0.2679 | 43 |
| PVP | 0.0984 | 0.0514 | 0.3431 | 21 |
| PVT | 0.0988 | 0.0483 | 0.3286 | 27 |
| REE | 0.0993 | 0.0542 | 0.3531 | 17 |
| SRF | 0.1117 | 0.0434 | 0.2800 | 42 |
| STG | 0.0927 | 0.0578 | 0.3840 | 10 |
| SVI | 0.0977 | 0.0573 | 0.3697 | 15 |
| TCL | 0.0859 | 0.0719 | 0.4554 | 5 |
| TDP | 0.1001 | 0.0608 | 0.3779 | 12 |
| THI | 0.1059 | 0.0441 | 0.2941 | 39 |
| TMS | 0.0767 | 0.0812 | 0.5142 | 2 |
| TV2 | 0.0942 | 0.0647 | 0.4073 | 8 |
| VIP | 0.1019 | 0.0503 | 0.3307 | 23 |
| VOS | 0.1033 | 0.0610 | 0.3712 | 14 |
| VTB | 0.1044 | 0.0587 | 0.3598 | 16 |
| VTO | 0.0968 | 0.0458 | 0.3211 | 30 |
| ARM | 0.1053 | 0.0444 | 0.2967 | 38 |
| BBS | 0.1051 | 0.0478 | 0.3125 | 35 |
| CDN | 0.0915 | 0.0582 | 0.3889 | 9 |
| CIA | 0.1255 | 0.0457 | 0.2670 | 44 |
| CTB | 0.1011 | 0.0538 | 0.3471 | 19 |
| CTT | 0.1085 | 0.0519 | 0.3234 | 29 |
| DL1 | 0.1077 | 0.0487 | 0.3113 | 36 |
| IPA | 0.0841 | 0.0894 | 0.5155 | 1 |
| PBP | 0.0916 | 0.0487 | 0.3470 | 20 |
| PMP | 0.1030 | 0.0505 | 0.3289 | 25 |
| PMS | 0.1008 | 0.0460 | 0.3133 | 34 |
| PRC | 0.1018 | 0.0472 | 0.3168 | 33 |
| PSC | 0.1002 | 0.0407 | 0.2886 | 41 |
| PTS | 0.0975 | 0.0478 | 0.3289 | 26 |
| SDC | 0.1049 | 0.0472 | 0.3102 | 37 |
| SDG | 0.1112 | 0.0309 | 0.2174 | 45 |
| TPP | 0.1042 | 0.0503 | 0.3254 | 28 |
| VBC | 0.1018 | 0.0500 | 0.3294 | 24 |
| VCM | 0.0981 | 0.0597 | 0.3785 | 11 |
| VSM | 0.0856 | 0.0654 | 0.4331 | 7 |

**Table S31. The normalized decision matrix in 2022 based on TOPSIS.**

| Company | Indicator | | | | | | | | | | | | | | |  |
| --- | --- | --- | --- | --- | --- | --- | --- | --- | --- | --- | --- | --- | --- | --- | --- | --- |
|  | I1 | I2 | I3 | I4 | I5 | I6 | I7 | I8 | I9 | I10 | I11 | I12 | I13 | I14 | I15 | |
| CAV | 0.1497 | 0.0806 | 0.1355 | 0.0253 | 0.0017 | 0.0465 | 0.0131 | 0.0246 | 0.0040 | 0.0940 | 0.0688 | 0.1610 | 0.0181 | 0.1154 | 0.1775 | |
| GEX | 0.0126 | 0.0077 | 0.0087 | 0.0374 | -0.0052 | 0.0418 | 0.0219 | 0.0122 | 0.0031 | 0.0630 | 0.0478 | 0.1433 | 0.0836 | 0.0672 | 0.1085 | |
| GMD | 0.0938 | 0.0996 | 0.0670 | 0.0663 | 0.0095 | 0.0298 | 0.0283 | 0.0530 | 0.0365 | 0.0309 | 0.0392 | 0.1003 | 0.2163 | 0.0331 | 0.1243 | |
| GSP | 0.0825 | 0.0693 | 0.0281 | 0.0402 | 0.0082 | 0.0560 | 0.0280 | 0.0233 | 0.0872 | 0.0998 | 0.0581 | 0.1633 | 0.0456 | 0.0366 | 0.0848 | |
| HAH | 0.2428 | 0.2363 | 0.2373 | 0.1966 | 0.0044 | 0.0698 | 0.0610 | 0.1121 | 0.0356 | 0.1355 | 0.0668 | 0.0998 | 0.0571 | 0.0258 | 0.0828 | |
| L10 | 0.0470 | 0.0174 | 0.0289 | -0.0103 | 0.0055 | 0.0567 | 0.0514 | 0.0187 | 0.0056 | 0.0464 | 0.0160 | 0.1728 | 0.0303 | 0.1782 | 0.1381 | |
| MSN | 0.0639 | 0.0318 | 0.0509 | -0.0459 | -0.0130 | 0.0265 | 0.0190 | 0.0099 | 0.0051 | 0.0614 | 0.1436 | 0.1299 | 0.0813 | 0.1459 | 0.1223 | |
| PAC | 0.1247 | 0.0768 | 0.0627 | 0.0266 | -0.0048 | 0.0418 | 0.0074 | 0.0231 | 0.0051 | 0.2100 | 0.1925 | 0.1539 | 0.0296 | 0.1280 | 0.1913 | |
| PDN | 0.2133 | 0.2343 | 0.2371 | 0.0603 | 0.0063 | 0.0582 | 0.0462 | 0.1033 | 0.8560 | 0.0623 | 0.0512 | 0.1180 | 0.0834 | 0.0290 | 0.1401 | |
| PJT | 0.0537 | 0.0533 | 0.0171 | 0.0954 | -0.0060 | 0.0360 | 0.0173 | 0.0145 | 0.0197 | 0.2208 | 0.2407 | 0.1654 | 0.0605 | 0.0291 | 0.1124 | |
| PVP | 0.0958 | 0.1057 | 0.0465 | 0.0606 | -0.0019 | 0.0901 | 0.0851 | 0.0889 | 0.1270 | 0.0419 | 0.0158 | 0.1671 | 0.0334 | 0.0286 | 0.1243 | |
| PVT | 0.0810 | 0.0763 | 0.0522 | 0.0654 | 0.0041 | 0.0749 | 0.0414 | 0.0376 | 0.0582 | 0.0857 | 0.0409 | 0.1466 | 0.0724 | 0.0312 | 0.0967 | |
| REE | 0.1071 | 0.0975 | 0.1534 | 0.1883 | 0.0006 | 0.0763 | 0.0581 | 0.0260 | 0.0057 | 0.0665 | 0.0196 | 0.0965 | 0.0991 | 0.0174 | 0.0552 | |
| SRF | -0.1993 | -0.0939 | -0.0807 | 0.0843 | -0.1229 | 0.0411 | 0.0315 | -0.0109 | 0.0050 | 0.0302 | 0.0080 | 0.1822 | 0.2175 | 0.2330 | 0.1854 | |
| STG | 0.0841 | 0.1038 | 0.0493 | -0.0263 | 0.0036 | 0.0745 | 0.0649 | 0.0896 | 0.0243 | 0.1072 | 0.0348 | 0.1487 | 0.0790 | 0.0187 | 0.1440 | |
| SVI | 0.1211 | 0.1167 | 0.1845 | 0.0069 | 0.0069 | 0.0687 | 0.0346 | 0.1432 | 0.0105 | 0.0434 | 0.0239 | 0.1539 | 0.0372 | 0.0546 | 0.1953 | |
| TCL | 0.1553 | 0.1740 | 0.0839 | 0.0439 | -0.0011 | 0.0669 | 0.0527 | 0.6016 | 0.1727 | 0.0735 | 0.0370 | 0.1488 | 0.0804 | 0.0296 | 0.1519 | |
| TDP | 0.0864 | 0.0368 | 0.0282 | 0.1017 | -0.0055 | 0.0382 | 0.0175 | 0.0085 | 0.0039 | 0.1363 | 0.0527 | 0.1590 | 0.0417 | 0.2232 | 0.1756 | |
| THI | 0.0226 | 0.0190 | 0.0182 | -0.1073 | -0.0079 | 0.0829 | 0.0417 | 0.0081 | 0.0020 | 0.0544 | 0.0374 | 0.1573 | 0.0957 | 0.0361 | 0.1223 | |
| TMS | 0.1223 | 0.1392 | 0.1268 | -0.1315 | 0.0267 | 0.0738 | 0.0624 | 0.0691 | 0.3057 | 0.0970 | 0.0352 | 0.1523 | 0.0766 | 0.0169 | 0.1184 | |
| TV2 | 0.0274 | 0.0169 | 0.0159 | -0.1954 | -0.0135 | 0.0476 | 0.0456 | 0.0549 | 0.0074 | 0.0057 | 0.0030 | 0.1542 | 0.1815 | 0.0963 | 0.1933 | |
| VIP | 0.1471 | 0.2011 | 0.0659 | 0.0877 | 0.5040 | 0.2552 | 0.0782 | 0.1361 | 0.0118 | 0.2995 | 0.0423 | 0.1479 | 0.0901 | 0.0080 | 0.1539 | |
| VOS | 0.2720 | 0.2142 | 0.0708 | 0.2151 | -0.0120 | 0.0691 | 0.0401 | 0.0538 | 0.0183 | 0.0401 | 0.0568 | 0.1247 | 0.0703 | 0.0338 | 0.1085 | |
| VTB | 0.0684 | 0.0881 | 0.0294 | -0.0745 | 0.0837 | 0.1683 | 0.0633 | 0.6482 | 0.0049 | 0.5598 | 0.0478 | 0.1257 | 0.1017 | 0.0123 | 0.1184 | |
| VTO | 0.0451 | 0.0506 | 0.0142 | 0.0760 | -0.0158 | 0.1079 | 0.1010 | 0.0223 | 0.0133 | 0.2071 | 0.0824 | 0.1496 | 0.1173 | 0.0180 | 0.0927 | |
| ARM | 0.0465 | 0.0236 | 0.0155 | -0.0178 | 0.0088 | 0.0476 | 0.0472 | 0.0338 | 0.0159 | 0.1271 | 0.0248 | 0.1517 | 0.1826 | 0.1881 | 0.1933 | |
| BBS | 0.0504 | 0.0252 | 0.0254 | -0.0440 | -0.0031 | 0.0363 | 0.0296 | 0.0092 | 0.0092 | 0.0562 | 0.0109 | 0.1628 | 0.0486 | 0.1738 | 0.1953 | |
| CDN | 0.1252 | 0.1681 | 0.0513 | 0.0335 | 0.0003 | 0.1403 | 0.0505 | 0.1876 | 0.0414 | 0.1067 | 0.0471 | 0.1101 | 0.1810 | 0.0119 | 0.0967 | |
| CIA | 0.1595 | 0.2333 | 0.0675 | 0.4782 | -0.0425 | 0.8461 | 0.9369 | 0.1027 | 0.0184 | 0.0799 | 0.0404 | 0.1742 | 0.2626 | 0.0029 | 0.1736 | |
| CTB | 0.1431 | 0.0737 | 0.0735 | 0.2068 | 0.0046 | 0.0400 | 0.0222 | 0.0273 | 0.0038 | 0.0439 | 0.0201 | 0.1542 | 0.0708 | 0.1653 | 0.1657 | |
| CTT | 0.1161 | 0.0208 | 0.0331 | 0.0284 | -0.0012 | 0.0320 | 0.0260 | 0.0100 | 0.0232 | 0.0637 | 0.0341 | 0.1693 | 0.0580 | 0.7384 | 0.1913 | |
| DL1 | 0.0259 | 0.0218 | 0.0087 | 0.5452 | -0.0136 | 0.0436 | 0.0452 | 0.0075 | 0.0080 | 0.0436 | 0.0757 | 0.0992 | 0.1510 | 0.0369 | 0.0730 | |
| IPA | 0.0178 | 0.0131 | 0.0090 | 0.0320 | -0.0269 | 0.1436 | 0.1567 | 0.0056 | 0.0157 | 0.2195 | 0.0453 | 0.0841 | 0.3415 | 0.0180 | 0.0335 | |
| PBP | 0.0746 | 0.0667 | 0.0286 | 0.0238 | 0.0023 | 0.0502 | 0.0068 | 0.1041 | 0.0110 | 0.1426 | 0.8750 | 0.1662 | 0.0571 | 0.0923 | 0.1953 | |
| PMP | 0.0756 | 0.0344 | 0.0294 | -0.0230 | 0.0043 | 0.0378 | 0.0228 | 0.0109 | 0.0071 | 0.1570 | 0.0402 | 0.1604 | 0.0807 | 0.2023 | 0.1913 | |
| PMS | 0.1033 | 0.0870 | 0.0676 | 0.2195 | -0.0122 | 0.0454 | 0.0298 | 0.0487 | 0.0235 | 0.2035 | 0.0865 | 0.1689 | 0.0389 | 0.0817 | 0.1953 | |
| PRC | 0.6641 | 0.7180 | 0.8436 | 0.0738 | 0.8472 | 0.1418 | 0.1166 | 0.1903 | 0.0312 | 0.2389 | 0.0308 | 0.1674 | 0.0969 | 0.0222 | 0.1460 | |
| PSC | 0.0332 | 0.0264 | 0.0153 | 0.2427 | -0.0175 | 0.0247 | 0.0210 | 0.0172 | 0.1516 | 0.2424 | 0.1646 | 0.1635 | 0.0465 | 0.0489 | 0.1085 | |
| PTS | 0.0654 | 0.0412 | 0.0288 | 0.0829 | -0.0106 | 0.0247 | 0.0198 | 0.0116 | 0.0309 | 0.0927 | 0.1400 | 0.1587 | 0.0920 | 0.0711 | 0.1045 | |
| SDC | 0.0227 | 0.0230 | 0.0086 | -0.0081 | -0.0076 | 0.0789 | 0.0542 | 0.0444 | 0.0024 | 0.0583 | 0.0035 | 0.1369 | 0.3383 | 0.0496 | 0.1736 | |
| SDG | 0.0499 | 0.0429 | 0.0648 | 0.0301 | 0.0177 | 0.0454 | 0.0281 | 0.0197 | 0.0105 | 0.1387 | 0.0318 | 0.1595 | 0.0487 | 0.0769 | 0.1854 | |
| TPP | 0.0252 | 0.0110 | 0.0066 | 0.1651 | 0.0047 | 0.0385 | 0.0209 | 0.0071 | 0.0058 | 0.0607 | 0.0322 | 0.1484 | 0.0488 | 0.2037 | 0.1677 | |
| VBC | 0.1319 | 0.0726 | 0.0591 | -0.0094 | 0.0000 | 0.0443 | 0.0318 | 0.0199 | 0.0097 | 0.0673 | 0.0208 | 0.1627 | 0.0299 | 0.1663 | 0.1953 | |
| VCM | 0.0023 | 0.0029 | 0.0015 | -0.1147 | -0.0183 | 0.0898 | 0.0327 | 0.0813 | 0.0499 | 0.0395 | 0.0149 | 0.1430 | 0.5647 | 0.0282 | 0.1677 | |
| VSM | 0.1537 | 0.1438 | 0.0908 | 0.0772 | -0.0026 | 0.0592 | 0.0620 | 0.1162 | 0.2838 | 0.0844 | 0.0329 | 0.1625 | 0.0413 | 0.0510 | 0.1539 | |

**Table S31. The weighted normalized decision matrix in 2022 based on TOPSIS.**

| Company | Indicator | | | | | | | | | | | | | | |  |
| --- | --- | --- | --- | --- | --- | --- | --- | --- | --- | --- | --- | --- | --- | --- | --- | --- |
|  | I1 | I2 | I3 | I4 | I5 | I6 | I7 | I8 | I9 | I10 | I11 | I12 | I13 | I14 | I15 | |
| CAV | 0.0061 | 0.0035 | 0.0062 | 0.0017 | 0.0001 | 0.0026 | 0.0007 | 0.0019 | 0.0003 | 0.0081 | 0.0046 | 0.0155 | 0.0015 | 0.0067 | 0.0185 | |
| GEX | 0.0005 | 0.0003 | 0.0004 | 0.0026 | -0.0003 | 0.0023 | 0.0012 | 0.0009 | 0.0002 | 0.0054 | 0.0032 | 0.0138 | 0.0069 | 0.0039 | 0.0113 | |
| GMD | 0.0038 | 0.0043 | 0.0031 | 0.0046 | 0.0005 | 0.0017 | 0.0016 | 0.0041 | 0.0023 | 0.0026 | 0.0026 | 0.0097 | 0.0179 | 0.0019 | 0.0129 | |
| GSP | 0.0034 | 0.0030 | 0.0013 | 0.0028 | 0.0004 | 0.0031 | 0.0015 | 0.0018 | 0.0056 | 0.0086 | 0.0039 | 0.0158 | 0.0038 | 0.0021 | 0.0088 | |
| HAH | 0.0099 | 0.0103 | 0.0109 | 0.0135 | 0.0002 | 0.0039 | 0.0034 | 0.0086 | 0.0023 | 0.0116 | 0.0045 | 0.0096 | 0.0047 | 0.0015 | 0.0086 | |
| L10 | 0.0019 | 0.0008 | 0.0013 | -0.0007 | 0.0003 | 0.0032 | 0.0028 | 0.0014 | 0.0004 | 0.0040 | 0.0011 | 0.0167 | 0.0025 | 0.0104 | 0.0144 | |
| MSN | 0.0026 | 0.0014 | 0.0023 | -0.0032 | -0.0007 | 0.0015 | 0.0010 | 0.0008 | 0.0003 | 0.0053 | 0.0097 | 0.0125 | 0.0067 | 0.0085 | 0.0127 | |
| PAC | 0.0051 | 0.0033 | 0.0029 | 0.0018 | -0.0003 | 0.0023 | 0.0004 | 0.0018 | 0.0003 | 0.0180 | 0.0130 | 0.0149 | 0.0024 | 0.0075 | 0.0199 | |
| PDN | 0.0087 | 0.0102 | 0.0109 | 0.0042 | 0.0003 | 0.0033 | 0.0025 | 0.0079 | 0.0548 | 0.0053 | 0.0035 | 0.0114 | 0.0069 | 0.0017 | 0.0146 | |
| PJT | 0.0022 | 0.0023 | 0.0008 | 0.0066 | -0.0003 | 0.0020 | 0.0010 | 0.0011 | 0.0013 | 0.0189 | 0.0162 | 0.0160 | 0.0050 | 0.0017 | 0.0117 | |
| PVP | 0.0039 | 0.0046 | 0.0021 | 0.0042 | -0.0001 | 0.0050 | 0.0047 | 0.0068 | 0.0081 | 0.0036 | 0.0011 | 0.0161 | 0.0028 | 0.0017 | 0.0129 | |
| PVT | 0.0033 | 0.0033 | 0.0024 | 0.0045 | 0.0002 | 0.0042 | 0.0023 | 0.0029 | 0.0037 | 0.0073 | 0.0028 | 0.0141 | 0.0060 | 0.0018 | 0.0100 | |
| REE | 0.0044 | 0.0042 | 0.0071 | 0.0130 | 0.0000 | 0.0043 | 0.0032 | 0.0020 | 0.0004 | 0.0057 | 0.0013 | 0.0093 | 0.0082 | 0.0010 | 0.0057 | |
| SRF | -0.0082 | -0.0041 | -0.0037 | 0.0058 | -0.0067 | 0.0023 | 0.0017 | -0.0008 | 0.0003 | 0.0026 | 0.0005 | 0.0176 | 0.0180 | 0.0136 | 0.0193 | |
| STG | 0.0034 | 0.0045 | 0.0023 | -0.0018 | 0.0002 | 0.0042 | 0.0036 | 0.0069 | 0.0016 | 0.0092 | 0.0023 | 0.0143 | 0.0065 | 0.0011 | 0.0150 | |
| SVI | 0.0050 | 0.0051 | 0.0085 | 0.0005 | 0.0004 | 0.0038 | 0.0019 | 0.0110 | 0.0007 | 0.0037 | 0.0016 | 0.0149 | 0.0031 | 0.0032 | 0.0203 | |
| TCL | 0.0064 | 0.0076 | 0.0039 | 0.0030 | -0.0001 | 0.0037 | 0.0029 | 0.0461 | 0.0111 | 0.0063 | 0.0025 | 0.0144 | 0.0066 | 0.0017 | 0.0158 | |
| TDP | 0.0035 | 0.0016 | 0.0013 | 0.0070 | -0.0003 | 0.0021 | 0.0010 | 0.0007 | 0.0002 | 0.0117 | 0.0036 | 0.0153 | 0.0034 | 0.0130 | 0.0183 | |
| THI | 0.0009 | 0.0008 | 0.0008 | -0.0074 | -0.0004 | 0.0046 | 0.0023 | 0.0006 | 0.0001 | 0.0047 | 0.0025 | 0.0152 | 0.0079 | 0.0021 | 0.0127 | |
| TMS | 0.0050 | 0.0060 | 0.0058 | -0.0091 | 0.0015 | 0.0041 | 0.0034 | 0.0053 | 0.0196 | 0.0083 | 0.0024 | 0.0147 | 0.0063 | 0.0010 | 0.0123 | |
| TV2 | 0.0011 | 0.0007 | 0.0007 | -0.0135 | -0.0007 | 0.0027 | 0.0025 | 0.0042 | 0.0005 | 0.0005 | 0.0002 | 0.0149 | 0.0150 | 0.0056 | 0.0201 | |
| VIP | 0.0060 | 0.0087 | 0.0030 | 0.0060 | 0.0274 | 0.0143 | 0.0043 | 0.0104 | 0.0008 | 0.0257 | 0.0029 | 0.0143 | 0.0074 | 0.0005 | 0.0160 | |
| VOS | 0.0111 | 0.0093 | 0.0033 | 0.0148 | -0.0007 | 0.0039 | 0.0022 | 0.0041 | 0.0012 | 0.0034 | 0.0038 | 0.0120 | 0.0058 | 0.0020 | 0.0113 | |
| VTB | 0.0028 | 0.0038 | 0.0014 | -0.0051 | 0.0046 | 0.0094 | 0.0035 | 0.0497 | 0.0003 | 0.0480 | 0.0032 | 0.0121 | 0.0084 | 0.0007 | 0.0123 | |
| VTO | 0.0018 | 0.0022 | 0.0007 | 0.0052 | -0.0009 | 0.0060 | 0.0056 | 0.0017 | 0.0009 | 0.0177 | 0.0056 | 0.0144 | 0.0097 | 0.0011 | 0.0096 | |
| ARM | 0.0019 | 0.0010 | 0.0007 | -0.0012 | 0.0005 | 0.0027 | 0.0026 | 0.0026 | 0.0010 | 0.0109 | 0.0017 | 0.0146 | 0.0151 | 0.0110 | 0.0201 | |
| BBS | 0.0021 | 0.0011 | 0.0012 | -0.0030 | -0.0002 | 0.0020 | 0.0016 | 0.0007 | 0.0006 | 0.0048 | 0.0007 | 0.0157 | 0.0040 | 0.0102 | 0.0203 | |
| CDN | 0.0051 | 0.0073 | 0.0024 | 0.0023 | 0.0000 | 0.0078 | 0.0028 | 0.0144 | 0.0027 | 0.0091 | 0.0032 | 0.0106 | 0.0149 | 0.0007 | 0.0100 | |
| CIA | 0.0065 | 0.0101 | 0.0031 | 0.0330 | -0.0023 | 0.0473 | 0.0516 | 0.0079 | 0.0012 | 0.0068 | 0.0027 | 0.0168 | 0.0217 | 0.0002 | 0.0180 | |
| CTB | 0.0059 | 0.0032 | 0.0034 | 0.0143 | 0.0002 | 0.0022 | 0.0012 | 0.0021 | 0.0002 | 0.0038 | 0.0014 | 0.0149 | 0.0058 | 0.0097 | 0.0172 | |
| CTT | 0.0048 | 0.0009 | 0.0015 | 0.0020 | -0.0001 | 0.0018 | 0.0014 | 0.0008 | 0.0015 | 0.0055 | 0.0023 | 0.0163 | 0.0048 | 0.0431 | 0.0199 | |
| DL1 | 0.0011 | 0.0009 | 0.0004 | 0.0376 | -0.0007 | 0.0024 | 0.0025 | 0.0006 | 0.0005 | 0.0037 | 0.0051 | 0.0096 | 0.0125 | 0.0022 | 0.0076 | |
| IPA | 0.0007 | 0.0006 | 0.0004 | 0.0022 | -0.0015 | 0.0080 | 0.0086 | 0.0004 | 0.0010 | 0.0188 | 0.0031 | 0.0081 | 0.0282 | 0.0011 | 0.0035 | |
| PBP | 0.0031 | 0.0029 | 0.0013 | 0.0016 | 0.0001 | 0.0028 | 0.0004 | 0.0080 | 0.0007 | 0.0122 | 0.0590 | 0.0160 | 0.0047 | 0.0054 | 0.0203 | |
| PMP | 0.0031 | 0.0015 | 0.0014 | -0.0016 | 0.0002 | 0.0021 | 0.0013 | 0.0008 | 0.0005 | 0.0134 | 0.0027 | 0.0155 | 0.0067 | 0.0118 | 0.0199 | |
| PMS | 0.0042 | 0.0038 | 0.0031 | 0.0151 | -0.0007 | 0.0025 | 0.0016 | 0.0037 | 0.0015 | 0.0174 | 0.0058 | 0.0163 | 0.0032 | 0.0048 | 0.0203 | |
| PRC | 0.0272 | 0.0312 | 0.0389 | 0.0051 | 0.0461 | 0.0079 | 0.0064 | 0.0146 | 0.0020 | 0.0205 | 0.0021 | 0.0162 | 0.0080 | 0.0013 | 0.0152 | |
| PSC | 0.0014 | 0.0011 | 0.0007 | 0.0167 | -0.0009 | 0.0014 | 0.0012 | 0.0013 | 0.0097 | 0.0208 | 0.0111 | 0.0158 | 0.0038 | 0.0029 | 0.0113 | |
| PTS | 0.0027 | 0.0018 | 0.0013 | 0.0057 | -0.0006 | 0.0014 | 0.0011 | 0.0009 | 0.0020 | 0.0079 | 0.0094 | 0.0153 | 0.0076 | 0.0042 | 0.0109 | |
| SDC | 0.0009 | 0.0010 | 0.0004 | -0.0006 | -0.0004 | 0.0044 | 0.0030 | 0.0034 | 0.0002 | 0.0050 | 0.0002 | 0.0132 | 0.0279 | 0.0029 | 0.0180 | |
| SDG | 0.0020 | 0.0019 | 0.0030 | 0.0021 | 0.0010 | 0.0025 | 0.0015 | 0.0015 | 0.0007 | 0.0119 | 0.0021 | 0.0154 | 0.0040 | 0.0045 | 0.0193 | |
| TPP | 0.0010 | 0.0005 | 0.0003 | 0.0114 | 0.0003 | 0.0022 | 0.0012 | 0.0005 | 0.0004 | 0.0052 | 0.0022 | 0.0143 | 0.0040 | 0.0119 | 0.0174 | |
| VBC | 0.0054 | 0.0032 | 0.0027 | -0.0006 | 0.0000 | 0.0025 | 0.0018 | 0.0015 | 0.0006 | 0.0058 | 0.0014 | 0.0157 | 0.0025 | 0.0097 | 0.0203 | |
| VCM | 0.0001 | 0.0001 | 0.0001 | -0.0079 | -0.0010 | 0.0050 | 0.0018 | 0.0062 | 0.0032 | 0.0034 | 0.0010 | 0.0138 | 0.0466 | 0.0016 | 0.0174 | |
| VSM | 0.0063 | 0.0062 | 0.0042 | 0.0053 | -0.0001 | 0.0033 | 0.0034 | 0.0089 | 0.0182 | 0.0072 | 0.0022 | 0.0157 | 0.0034 | 0.0030 | 0.0160 | |
| **PIS** | **0.0272** | **0.0312** | **0.0389** | **0.0376** | **0.0461** | **0.0473** | **0.0516** | **0.0497** | **0.0548** | **0.0005** | **0.0590** | **0.0081** | **0.0015** | **0.0002** | **0.0035** | |
| **NIS** | **-0.0082** | **-0.0041** | **-0.0037** | **-0.0135** | **-0.0067** | **0.0014** | **0.0004** | **-0.0008** | **0.0001** | **0.0480** | **0.0002** | **0.0176** | **0.0466** | **0.0431** | **0.0203** | |

**Table S32. The distance from each alternative to PIS and NIS, and rank in 2022 based on TOPSIS.**

| Company | S_i*_ | S_i-_ | Score | Rank |
| --- | --- | --- | --- | --- |
| CAV | 0.1373 | 0.0507 | 0.2697 | 21 |
| GEX | 0.1404 | 0.0504 | 0.2642 | 27 |
| GMD | 0.1367 | 0.0548 | 0.2863 | 16 |
| GSP | 0.1358 | 0.0497 | 0.2679 | 23 |
| HAH | 0.1264 | 0.0581 | 0.3148 | 7 |
| L10 | 0.1408 | 0.0511 | 0.2664 | 24 |
| MSN | 0.1391 | 0.0493 | 0.2618 | 30 |
| PAC | 0.1367 | 0.0415 | 0.2331 | 43 |
| PDN | 0.1189 | 0.0805 | 0.4038 | 3 |
| PJT | 0.1348 | 0.0420 | 0.2375 | 40 |
| PVP | 0.1318 | 0.0560 | 0.2981 | 13 |
| PVT | 0.1352 | 0.0513 | 0.2749 | 20 |
| REE | 0.1335 | 0.0574 | 0.3006 | 10 |
| SRF | 0.1495 | 0.0510 | 0.2544 | 35 |
| STG | 0.1363 | 0.0483 | 0.2614 | 31 |
| SVI | 0.1341 | 0.0560 | 0.2945 | 14 |
| TCL | 0.1232 | 0.0726 | 0.3706 | 4 |
| TDP | 0.1398 | 0.0472 | 0.2524 | 37 |
| THI | 0.1424 | 0.0490 | 0.2561 | 33 |
| TMS | 0.1311 | 0.0530 | 0.2881 | 15 |
| TV2 | 0.1458 | 0.0519 | 0.2625 | 29 |
| VIP | 0.1240 | 0.0559 | 0.3108 | 8 |
| VOS | 0.1309 | 0.0623 | 0.3223 | 6 |
| VTB | 0.1361 | 0.0584 | 0.3002 | 11 |
| VTO | 0.1361 | 0.0422 | 0.2365 | 41 |
| ARM | 0.1418 | 0.0438 | 0.2359 | 42 |
| BBS | 0.1431 | 0.0495 | 0.2570 | 32 |
| CDN | 0.1307 | 0.0522 | 0.2856 | 17 |
| CIA | 0.1142 | 0.0981 | 0.4621 | 1 |
| CTB | 0.1365 | 0.0583 | 0.2993 | 12 |
| CTT | 0.1465 | 0.0498 | 0.2538 | 36 |
| DL1 | 0.1346 | 0.0719 | 0.3482 | 5 |
| IPA | 0.1397 | 0.0393 | 0.2196 | 45 |
| PBP | 0.1264 | 0.0494 | 0.2811 | 18 |
| PMP | 0.1423 | 0.0422 | 0.2287 | 44 |
| PMS | 0.1349 | 0.0489 | 0.2662 | 25 |
| PRC | 0.1120 | 0.0955 | 0.4603 | 2 |
| PSC | 0.1324 | 0.0475 | 0.2638 | 28 |
| PTS | 0.1363 | 0.0500 | 0.2685 | 22 |
| SDC | 0.1430 | 0.0490 | 0.2553 | 34 |
| SDG | 0.1397 | 0.0457 | 0.2464 | 39 |
| TPP | 0.1396 | 0.0540 | 0.2788 | 19 |
| VBC | 0.1403 | 0.0508 | 0.2658 | 26 |
| VCM | 0.1481 | 0.0489 | 0.2482 | 38 |
| VSM | 0.1273 | 0.0570 | 0.3095 | 9 |

**Table S33. The variance of evaluation variables based on Statistical Variance Procedure.**

| Year | Indicator | | | | | | | | | | | | | | |  |
| --- | --- | --- | --- | --- | --- | --- | --- | --- | --- | --- | --- | --- | --- | --- | --- | --- |
|  | I1 | I2 | I3 | I4 | I5 | I6 | I7 | I8 | I9 | I10 | I11 | I12 | I13 | I14 | I15 | |
| 2020 | 0.0261 | 0.0317 | 0.0367 | 0.0242 | 0.0275 | 0.0300 | 0.0390 | 0.0305 | 0.0332 | 0.0214 | 0.0503 | 0.0385 | 0.0421 | 0.0322 | 0.1094 | |
| 2021 | 0.0267 | 0.0264 | 0.0531 | 0.0419 | 0.0221 | 0.0467 | 0.0308 | 0.0439 | 0.0342 | 0.0506 | 0.0359 | 0.0186 | 0.0264 | 0.0313 | 0.0853 | |
| 2022 | 0.0173 | 0.0216 | 0.0203 | 0.0336 | 0.0234 | 0.0228 | 0.0215 | 0.0387 | 0.0265 | 0.0306 | 0.0229 | 0.0575 | 0.0358 | 0.0265 | 0.0719 | |
